# An Aluminum-Based Lewis Superacid and Its Weakly Coordinating Anions Derived from an Organotellurium Ligand

**DOI:** 10.1021/jacsau.5c00577

**Published:** 2025-06-23

**Authors:** Daniel Wegener, Niklas Limberg, Moritz Bubenik, Alberto Pérez-Bitrián, Anja Wiesner, Sebastian Riedel

**Affiliations:** Fachbereich Biologie, Chemie, Pharmazie, Institut für Chemie und Biochemie − Anorganische Chemie, 9166 Freie Universität Berlin, Fabeckstraße 34/36, Berlin 14195, Germany

**Keywords:** Lewis superacids, aluminum, fluorine chemistry, weakly coordinating anions, organotellurium

## Abstract

Lewis acids and their related weakly coordinating anions (WCAs) are central species in chemistry, and tuning their properties toward different purposes is still a challenging field of research. In this work, the properties of the Lewis acid Al­(OTe^R^)_3_ have been investigated using the advantages of the OTeF_3_(C_6_F_5_)_2_ ligand (OTe^R^). Its Lewis acidity was evaluated by means of fluoride ion affinity (FIA) calculations, indicating that it is a Lewis superacid. Complementary analysis using the Gutmann–Beckett method by the synthesis of the Al­(OTe^R^)_3_·OPEt_3_ adduct rendered similar results, yet for the heavier Ga species this adduct formation was not possible, as only GaEt­(OTe^R^)_2_·OPEt_3_ was obtained. The reported new Lewis acid was further stabilized as acid–base adducts with tetrahydrofuran and dimethyl carbonate. The isolation of the free Lewis acid proved challenging due to fluoride abstraction from its own ligand, as shown by quantum-chemical calculations. Derived from the Lewis superacid Al­(OTe^R^)_3_ two weakly coordinating anions, the fluoride adduct [FAl­(OTe^R^)_3_]^−^ and the even less coordinating mixed anion [(F_5_TeO)­Al­(OTe^R^)_3_]^−^, were synthesized. Among them, the synthetically useful silver salt Ag­[(F_5_TeO)­Al­(OTe^R^)_3_] stands out, which could be used to generate a strong Brønsted acid by reaction with HCl as well as the [Ph_3_C]^+^ cation via reaction with Ph_3_CCl. Electrostatic potential surface analysis confirmed the more efficient delocalization of the negative charge and enhanced shielding of oxygen atoms in [(F_5_TeO)­Al­(OTe^R^)_3_]^−^ compared to [FAl­(OTe^R^)_3_]^−^ and [Al­(OTeF_5_)_4_]^−^, and therefore its potential as a promising new WCA.

## Graphical abstract

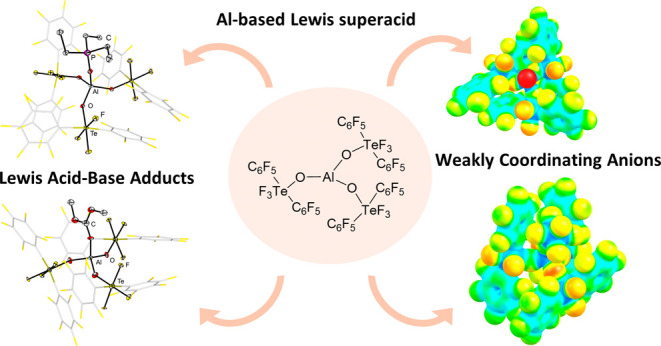

## Introduction

The field of Lewis superacids has significantly increased its popularity in the recent years due to its value in advancing fundamental research and its unequaled performance in chemical transformations.^1–5^ A widely accepted definition of Lewis superacids identifies them as molecular compounds with a higher fluoride ion affinity (FIA) than monomeric SbF_5_ in the gas phase.^6^ However, since the hard fluoride ion is less suitable for evaluating soft Lewis acids, the hydride ion affinity (HIA) was introduced as an alternative scale. Soft Lewis superacids are thus defined as compounds with a higher HIA than B­(C_6_F_5_)_3_ in the gas phase. Together with FIA or HIA, which are classified as global methods, influenced by adduct formation, effective scales also rely on such interaction but investigate the changes of spectroscopic properties. In this regard, the Gutmann–Beckett method is a clear example. In contrast to all these, the global electrophilicity index (GEI) offers an intrinsic measure of Lewis acidity based on electron affinity, providing a more fundamental, interaction-free assessment.^1,7^ Structurally, such superacids are predominantly based on the triels as the central atom, being coordinated by a variety of electron-withdrawing ligands with different donor atoms. Among them, recent examples include B­(C_6_F_4_CF_3_)_3_,^8^ B*o*Cb_3_ (*o*Cb = *o*-carborane, C_2_B_10_H_11_),^3^ Al­(C_6_F_5_)_3_,^9^ Al­(OC­(CF_3_)_3_)_3_,^6^ Al­(OTeF_5_)_3_,^10,11^ Al­(OC­(C_6_F_5_)_3_)_3_,^12^ Al­(N­(C_6_F_5_)_2_)_3_,^13^ Ga­(C_2_F_5_)_3_ ^14^ and Ga­(OTeF_5_)_3_ ^15^ (selected examples are shown in Figure 1).

**Figure 1:**
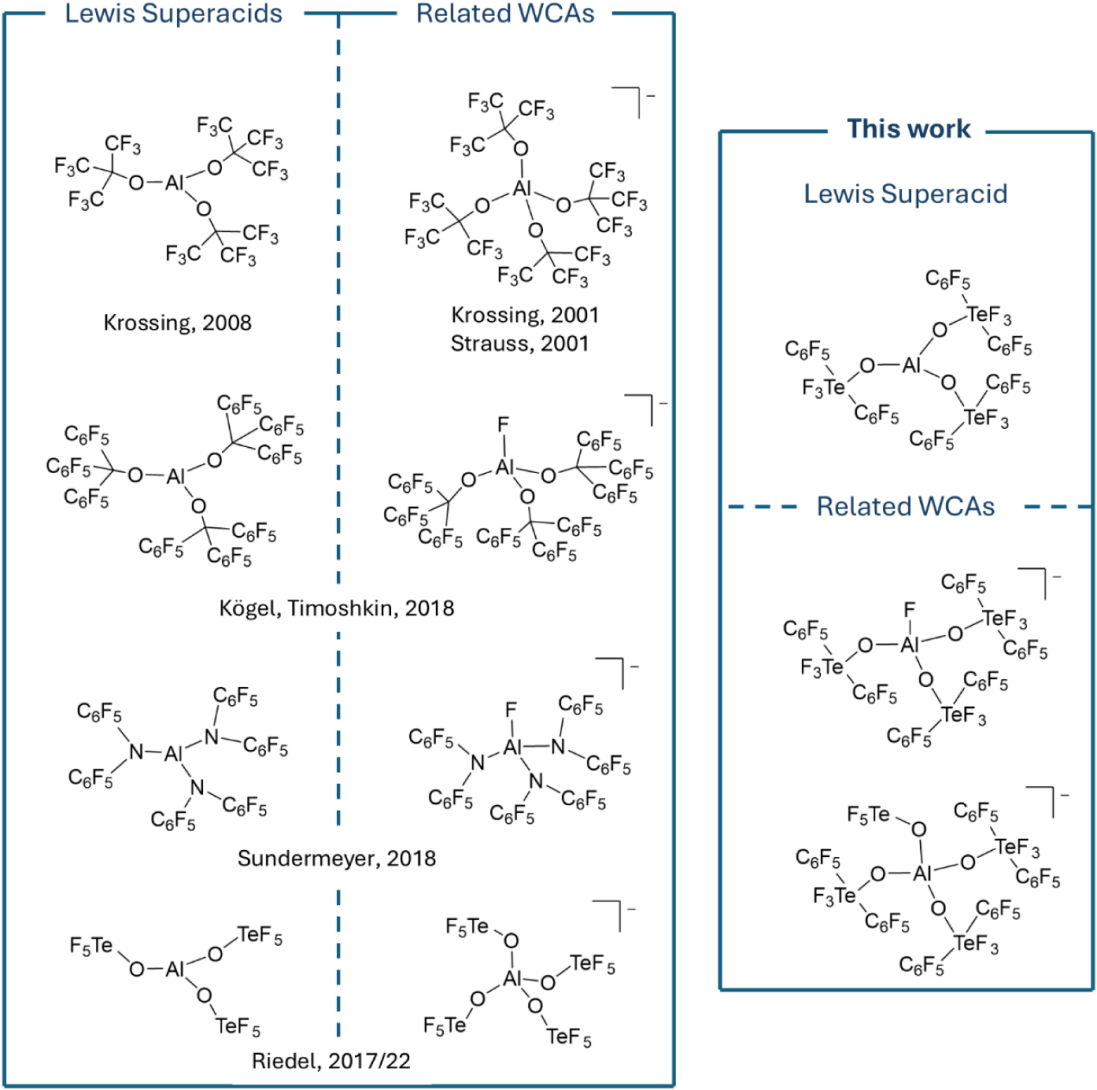
Selected modern aluminum-based Lewis superacids and their related weakly coordinating anions (WCAs).

The case of aluminum-based Lewis superacids is particularly challenging since some of them suffer from intrinsic instability, preventing their isolation as pure substances. The superacid Al­(OC­(CF_3_)_3_)_3_ was first synthesized in 2008,^6^ yet has never been isolated as a neat substance due to its limited thermal stability. Instead, it is typically stabilized as an adduct with weak bases such as fluorobenzene,^6^ trimethylsilyl fluoride^16^ or dimethyl sulfide.^17^ An O-donor ligand with highly electron withdrawing properties as well as stability against oxidation is the teflate group (OTeF_5_),^18,19^ which therefore usually enables the formation of especially strong Lewis acids. In this context, the teflate-based Lewis superacid Al­(OTeF_5_)_3_ exhibits a very high Lewis acidity. However, it is lowered by its tendency to dimerize despite the considerable steric bulk of the teflate group. Similar to Al­(OC­(CF_3_)_3_)_3_, Al­(OTeF_5_)_3_ also shows some decomposition under ambient temperatures for prolonged periods of time.^10,11^ Several solvent adducts of Al­(OTeF_5_)_3_ including SO_2_ClF or fluorobenzene have been synthesized, though these weakly bound adducts are also labile under standard conditions.^11^ These limitations highlight that a more sterically demanding teflate analogue that retains the unique electronic characteristics of the original group while providing greater steric hindrance may enhance stability and suppress oligomerization of a Lewis acid.

Closely related to Lewis acids are weakly coordinating anions (WCAs), which are formally formed by the addition of an anionic group to a Lewis acid (Figure 1). Alongside [B­(C_6_F_5_)_4_]^−^, which is arguably the most commonly used WCA, carboranes and aluminum-based WCAs are also often utilized in the stabilization of highly reactive cations.^20–23^ For instance, the aluminum-based anion [Al­(OC­(CF_3_)_3_)_4_]^−^ ^100^ has been shown to stabilize a plethora of reactive cations including extremely electrophilic [CCl_3_]^+^ and [CBr_3_]^+^ ^24^ or [P_9_]^+^,^25^ or the highly oxidizing [Ag­(X_2_)*_n_*]^+^ (X = Cl, Br, I),^26,27^ [NO]^+^ ^28^ and [NO_2_]^+^ ^29^ cations. However, small cations tend to preferentially coordinate the most basic regions of the WCAs, often leading to decomposition.

In fact, [Al­(OC­(CF_3_)_3_)_4_]^−^ decomposes when exposed to small silylium ions such as [Me_3_Si]^+^, resulting in the formation of Me_3_Si–F–Al­(OC­(CF_3_)_3_)_3_.^16^ Related to this example, [Al­(OTeF_5_)_4_]^−^ fails to stabilize the highly reactive [Et_3_Si]^+^ cation as well, resulting in the decomposition of the anion and formation of Et_3_SiOTeF_5_, indicating insufficient shielding of the basic oxygen atoms of the teflate groups.^11^ In addition, molecular structures derived from single-crystal X-ray diffraction based on WCAs such as [FAl­(OC­(CF_3_)_3_)_3_]^−^ and [Al­(OC­(CF_3_)_3_)_4_]^−^ sometimes suffer from disorder, complicating the structural investigation.^30^ Recent progress in addressing the aforementioned problems can be found in the fluoride-bridged derivative [((F_3_C)_3_CO)_3_Al–F–Al­(OC­(CF_3_)_3_)_3_]^−^ or the perfluoro-1-adamantoxy-based WCA [Al­(OC_10_F_15_)_4_]^−^.^30,31^

A few years ago, we developed the tellurium-based ligand OTeF_3_(C_6_F_5_)_2_ ^32^ – from now on abbreviated as OTe^R^ – which provides strong electron-withdrawing properties coupled with significant steric protection of the acidic center to which it is bonded, as demonstrated by the boron Lewis acid B­(OTe^R^)_3_.^33^ This unique combination of properties inspired us to explore the possibility of forming an Al-based Lewis superacid and its corresponding WCAs with improved properties by using this ligand system. Herein, we present the synthesis of Lewis acid–base adducts of the new Lewis superacid Al­(OTe^R^)_3_, the evaluation of its Lewis acidity and its application in the synthesis of WCAs, along with the first examples of their use to stabilize unusual cations.

## Results and Discussion

### The Lewis Superacid Al­(OTe^R^)_3_

The free Lewis acid Al­(OTe^R^)_3_ (**1**) was targeted through the reaction of AlEt_3_ with three equivalents of HOTe^R^ (**2**), whereby **1** should be generated upon release of ethane (Figure 2). In fact, if this reaction was performed in weakly coordinating solvents such as toluene or dichloromethane, the formation of several different undefined species with Al–F bonds was observed in the ^19^F and ^27^Al NMR spectra. Unfortunately, isolation of **1** as a neat substance has not been successful at ambient or low (−40 °C) temperature thus far.

**Figure 2:**
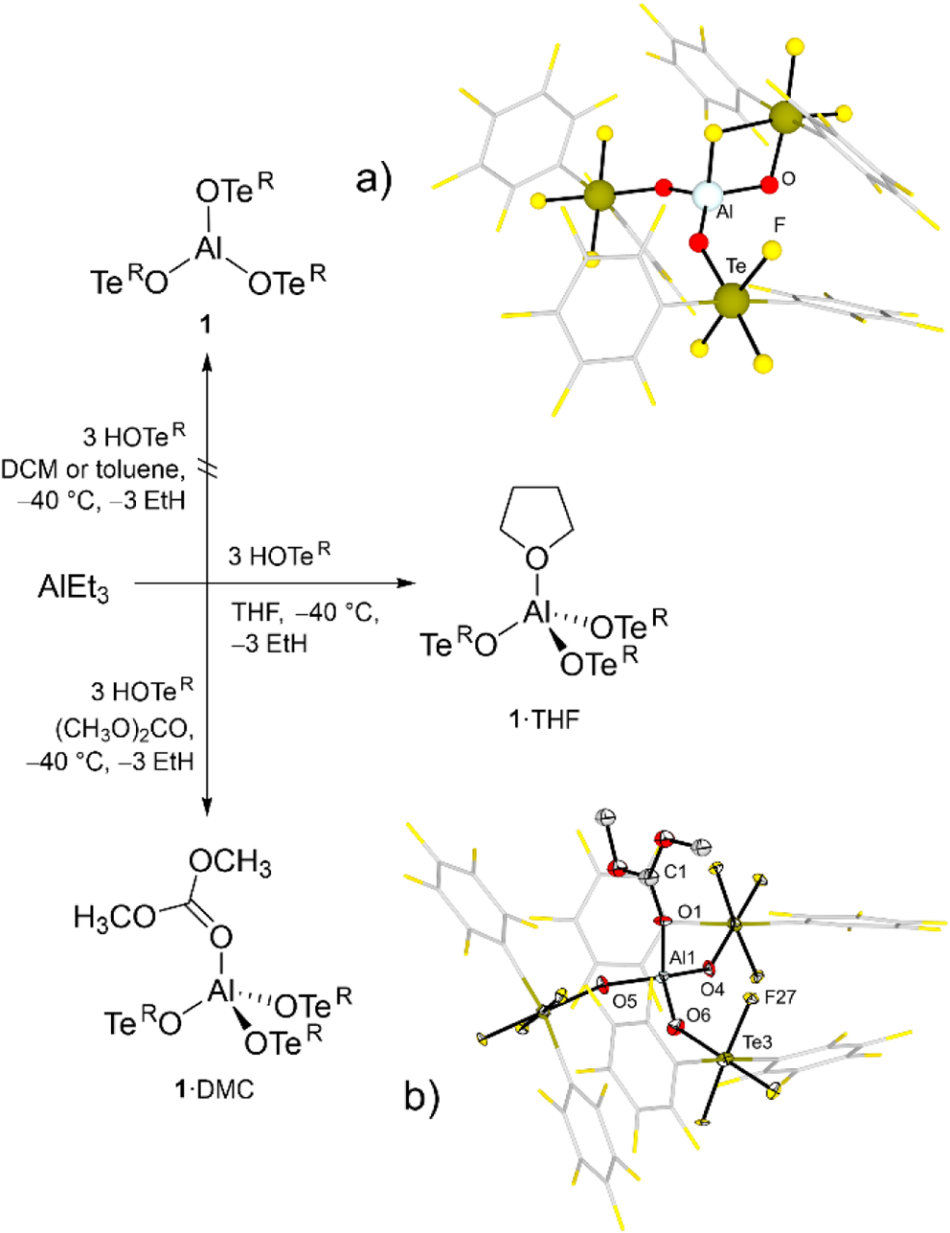
Attempted synthesis of adduct-free Al­(OTe^R^)_3_ (**1**) and formation of the adducts **1**·THF and **1**·DMC. (a) Optimized structure of **1** at the BP86/def-SV­(P) level. (b) Molecular structure in the solid state of **1**·DMC. Displacement ellipsoids set at 50% probability. Selected bond lengths [pm]: Al1–O1 179.9(7), Al1–O4 173.9(7), Al1–O5 172.7(7), Al1–O6 169.8(7).

Intrigued by this observation, DFT calculations have been performed to optimize the structure of **1** showing the Al–F bond formation. The induced high acidity at the Al center accounts for the self-decomposition of the free Lewis acid, since an Al–F bond formation (186.5 pm) is observed, together with a Te–F bond elongation (221.7 pm) (Figure 2a). The Al–F distance is significantly shorter than the contacts found in the calculated structure of Al­(OC­(C_6_F_5_)_3_)_3_ (213.1 and 217.3 pm)^6^ or Al­(OC­(CF_3_)_3_)_3_ (214.3 and 215.5 pm), which suffers from self-decomposition as well.^12^ This indicates that the aluminum center is so Lewis acidic that it abstracts one of the fluorides from the ligand, hence its computed structure can be interpreted as an intermediate for its decomposition. Additionally, the fragmented ligand may not be stable and therefore undergo reductive elimination to form a Te­(IV) species, as shown for PhTeF_5_ and PhTeF_4_CF_3_ upon fluoride abstraction.^34^

To prevent the self-coordination of the ligand to the aluminum center, the reaction described above was carried out in the presence of an O-donor solvent in order to block the Lewis acidic site (Figure 2). Our solvents of choice were tetrahydrofuran (THF) and dimethylcarbonate (DMC), since several other solvents such as MeCN, Et_2_O, Me_2_S, TMSF, SO_2_ClF or C_6_H_5_F only gave unsatisfying results. Here, the adduct formation between the base and AlEt_3_ presumably occurs first, followed by protonation of the ethyl groups leading to the formation of the corresponding adduct of the Lewis acid, namely Al­(OTe^R^)_3_·THF (**1**·THF) and Al­(OTe^R^)_3_·DMC (**1**·DMC).

The ^19^F NMR spectra of these species show the typical pattern for an OTe^R^ ligand with the triplet of quintets deshielded by around 8 ppm compared to the starting material HOTe^R^.^32^ The ^27^Al NMR spectra show a singlet at around 44 ppm, which is in the typical region for four-coordinated Al species.^10^ These adducts could be isolated as solids and stored under an inert atmosphere for, at least, months. **1**·DMC could be additionally crystallized by slow diffusion of *n*-pentane into a CH_2_Cl_2_ solution of the adduct. The molecular structure in the solid state reveals a distorted tetrahedral coordination around the Al center with Al–O_OTeR_ bond lengths of 169.9(7) to 173.9(7) pm and an Al–O_DMC_ bond length of 179.9(7) pm (see Figure 2b), which is similar to that found in Al­(OC­(CF_3_)_3_)_3_·DMC (180.9(9) pm).^30^

To determine the Lewis acidity of the target Lewis acid, we performed FIA calculations at the BP86/def-SV­(P) and additionally at the PW6B95-(D4)/def2-QZVPP//PBEh-3c/def2-mSVP level, using the Me_3_SiF/Me_3_Si^+^ system as an anchor point,^35,36^ and compared them to a set of different aluminum-based Lewis acids. These calculations demonstrate that neat monomeric **1** exhibits a relatively high FIA value of 531 kJ mol^–1^ (554 kJ mol^–1^ at the PW6B95-(D4)/def2-QZVPP//PBEh-3c/def2-mSVP level), surpassing the benchmark for Lewis superacidity set by SbF_5_ (493 kJ mol^–1^), and falling within the same range as Al­(OC­(CF_3_)_3_)_3_ (543 kJ mol^–1^) and Al­(OC­(C_6_F_5_)_3_)_3_ (525 kJ mol^–1^).^11,36^ Compared to the closely related Al­(OTeF_5_)_3_ (591 kJ mol^–1^),^11^ the FIA of **1** is lower, yet it remains approximately 90% of its value.

Experimentally, the Lewis acidity was assessed by the Gutmann–Beckett method, which is based on the change in the chemical shift of the ^31^P NMR signal of Et_3_PO upon adduct formation with a Lewis acid.^37,38^ In case of **1**, this adduct can be formed in two ways (Figure 3). The first method involves the addition of Et_3_PO to a solution of **1**·THF in DCM, leading to the replacement of THF by Et_3_PO.

**Figure 3:**
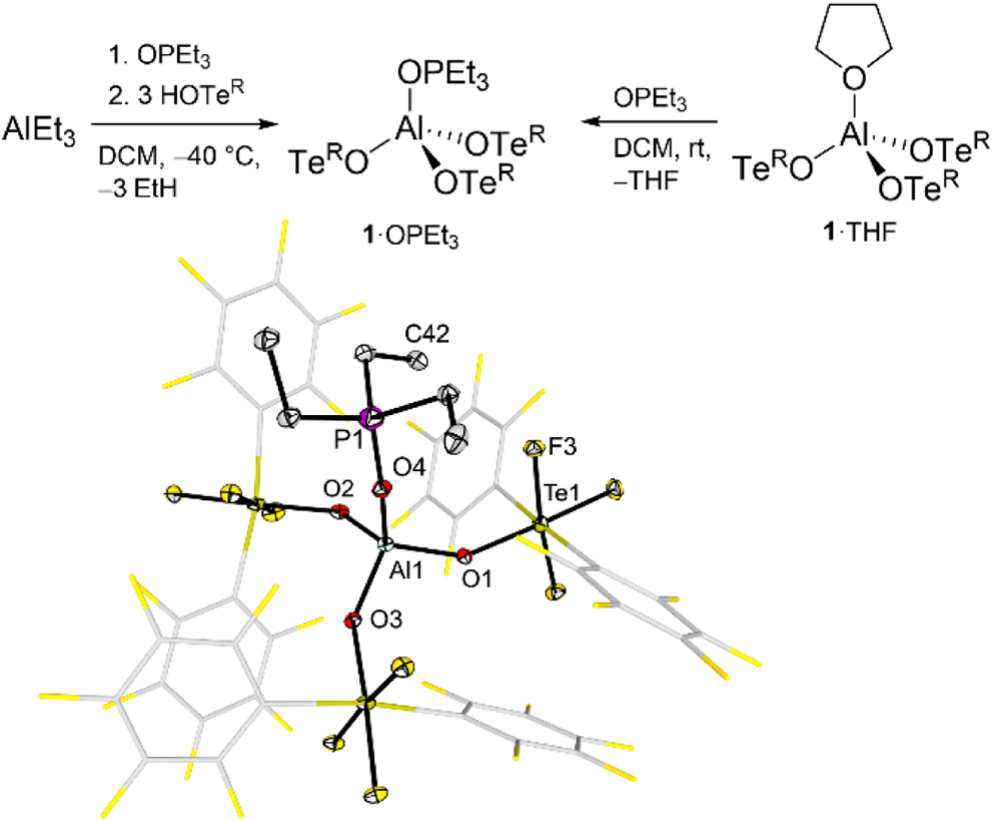
Synthetic approaches for the formation of the **1**·OPEt_3_ adduct and its molecular structure in the solid state. Displacement ellipsoids set at 50% probability. Selected bond lengths [pm]: Al1–O1 173.8(3), Al1–O2 175.2(3), Al1–O3 173.7(3), Al1–O4 174.5(3).

The second method consists of the addition of Et_3_PO to an AlEt_3_ solution initially, whereby the AlEt_3_·OPEt_3_ adduct^39^ is formed to prevent self-decomposition, followed by reaction with three equivalents of **2**. The target compound Al­(OTe^R^)_3_·OPEt_3_ (**1**·OPEt_3_) was formed selectively upon release of ethane and could be isolated quantitatively. In the ^19^F NMR spectrum, a shift of the signals is observed compared to acid **2** and the signals of the starting material **2** vanish. The ^27^Al NMR spectrum shows a signal at 39 ppm, being in the region for four-coordinated Al species as already seen in the case of **1**·THF and **1**·DMC. The ^31^P NMR spectrum of **1**·OPEt_3_ displays a signal at 77.8 ppm, resulting in a Δδ_P_ value of 27.8 ppm, which is similar to the shift observed for Al­(OC­(CF_3_)_3_)_3_ (28.0 ppm).^17^ Interestingly, it surpasses the shift caused by Al­(OC­(C_6_F_5_)_3_)_3_ (23.9 ppm),^12^ whereas Al­(OTeF_5_)_3_ induces the highest Δδ_P_ (33.9 ppm),^11^ displaying the highest Lewis acidity among these examples.

Single crystals of **1**·OPEt_3_ suitable for X-ray diffraction were grown by layering a solution of **1**·OPEt_3_ in DCM with *n*-pentane. The molecular structure in the solid state reveals a distorted tetrahedrally coordinated aluminum center with Al–O_OTeR_ bond lengths of 173.7(3) to 175.2(3) pm and an Al–O_OPEt3_ bond length of 174.5(3) pm (see Figure 3), being similar to those found in Al­(OTeF_5_)_3_·OPEt_3_ (173.3(3) to 174.1(3) pm and *d*(Al–O_OPEt3_) = 171.3(3)).^11^

The Gutmann–Beckett experiment was additionally carried out with the more sterically demanding Ph_3_PO to better assess the limit of steric crowding at the Al center. If the adduct AlEt_3_·OPPh_3_ formed by reaction of Ph_3_PO with AlEt_3_ ^40^ is reacted with three equivalents of **2**, only two of the three ethyl groups are substituted, resulting in the formation of AlEt­(OTe^R^)_2_·OPPh_3_ (**3**, Figure 4a). This finding can be attributed to the steric demand of the OTe^R^ ligand, which prevents the substitution of the third ethyl group by an OTe^R^ ligand. In fact, the ethyl group cannot even be protonated by **2** with an increase in the reaction temperature to 80 °C. In contrast to the former observation (Figure 3), **1**·THF does not react with the bulkier OPPh_3_. Single crystals suitable for X-ray diffraction were grown by slow diffusion of *n*-pentane into a DCM solution of **3**. The molecular structure reveals the distorted tetrahedrally coordinated Al center with Al–O bond lengths in the range of 175.5(4) pm to 177.6(4) pm (Figure 4b). The Al–C bond length of 194.4(5) pm is similar to those described in (Et)_2_Al­(μ–OR_F_)_2_Al­(Et)­(OR_F_) (OR_F_ = OC­(CF_3_)_3_) (*d*(Al–C) = 192.6(3) to 194.7(3) pm).^41^

**Figure 4:**
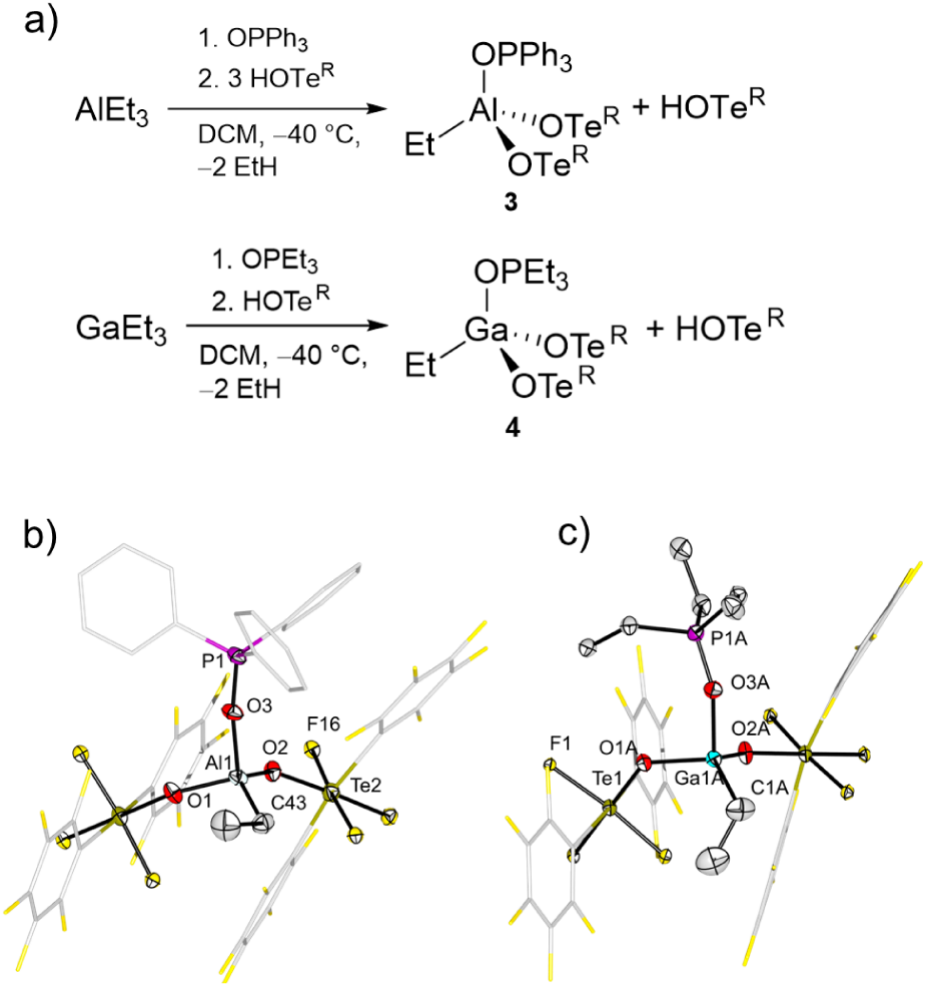
(a) Reaction of AlEt_3_ with Ph_3_PO and acid **2** and GaEt_3_ with Et_3_PO and acid **2**. (b) Molecular structure in the solid state of AlEt­(OTe^R^)_2_·OPPh_3_ (**3**). Displacement ellipsoids set at 50% probability. Selected bond lengths [pm]: Al1–O1 175.5(4), Al1–O2 177.6(4), Al1–O3 177.5(4), Al1–C43 194.4(5). (c) Molecular structure in the solid state of GaEt­(OTe^R^)_2_·OPEt_3_ (**4**). Displacement ellipsoids set at 50% probability. Only one set of disordered atoms is shown. Selected bond lengths [pm]: Ga1A–O1A 186.9(2), Ga1A–O2A 186.1(3), Ga1A–O3A 188.8(2), Ga1A–C1A 195.6(4).

To further evaluate the steric profile of OTe^R^ at the Al center, we used the method recently reported by Finze and Radius,^42^ utilizing the SambVca 2.1 tool.^43^ Using this approach, the buried volume of the fluoride adduct was determined to be %V_bur_ = 69.1%. This indicates a much greater steric hindrance at the aluminum center than that caused by the OTeF_5_ group in Al­(OTeF_5_)_3_ (%V_bur_ = 51.5%) or the OC­(CF_3_)_3_ ligands in Al­(OC­(CF_3_)_3_)_3_ (%V_bur_ = 56.8%). On the other hand, the obtained value is similar to that of Al­(N­(C_6_F_5_)_2_)_3_ (%V_bur_ = 68.1%) but less than that of Al­(OC­(C_6_F_5_)_3_)_3_ (%V_bur_ = 77.5%).

The high steric demand of the ligand prompted us to change the Al atom for the heavier analogue Ga, aiming at a larger coordination environment where the three OTe^R^ ligands and the phosphine oxide might fit, and therefore compare their Lewis acidities. Similarly to the synthesis of **1**·OPEt_3_, GaEt_3_·OPEt_3_ can be formed by reaction of Et_3_PO with GaEt_3_ and then reacted with three equivalents of **2**. However, only two ethyl groups are cleaved already in the case of the less steric Et_3_PO and GaEt­(OTe^R^)_2_·OPEt_3_ (**4**) is obtained (Figure 4a). Single crystals suitable for X-ray diffraction were grown by slow diffusion of *n*-pentane into a solution of **4** in DCM. The Ga center exhibits a distorted tetrahedral coordination with Ga–O bond lengths of 186.1(3) to 188.8(2) pm and a Ga–C bond length of 195.6(4) pm (see Figure 4c), compared to 194.3(3) pm in [Ga­(OTeF_5_)_3_Et]^−^.^44^ The remaining ethyl group cannot be substituted, even with increased temperature or extended reaction time. This is not surprising, since a similar situation was reported in the literature for the related [Ga­(OTeF_5_)_3_Et]^−^ anion, where substituting the last ethyl group with a teflate ligand is also unfeasible, indicating that not only steric factors play a role in the case of the softer Ga center. Ultimately, Ga­(OTeF_5_)_3_ was only successfully obtained through the reaction of GaCl_3_ with ClOTeF_5_.^15^

### Weakly Coordinating Anions

Weakly coordinating anions are formally created by the addition of an anionic group to a Lewis acid, as for instance in the case of the weakly coordinating [Al­(OTeF_5_)_4_]^−^ anion, which can be formed by addition of an OTeF_5_-containing compound like [NEt_4_]­[OTeF_5_] to the Lewis acid Al­(OTeF_5_)_3_.^11,45^ Inspired by this idea, the synthesis of the homoleptic WCA [Al­(OTe^R^)_4_]^−^ was attempted by substitution of THF in **1**·THF with an [OTe^R^]^−^ anion. Instead of the homoleptic WCA, the fluoride adduct [FAl­(OTe^R^)_3_]^−^ was obtained, most probably due to a fluoride ion abstraction from the [OTe^R^]^−^ anion by the Lewis acidic Al center (Scheme 1). The formation of the fluoride adduct is evident in the ^19^F NMR spectrum, which displays not only signals for the tellurium-bound fluorine atoms (δ = 11.7 ppm and δ = −22.5 ppm) and for fluorine atoms in C_6_F_5_ groups (δ = −131 ppm, −150 ppm and −162 ppm), but also a signal for a fluoride connected to the aluminum center (δ = −176 ppm), with an integral ratio of 3:6:12:6:12:1. This result indicates that four ligands may not fit around the Al center due to steric overcrowding, and points toward the formation of the fluoride adduct as a more favorable situation arising from the high Lewis acidity of the aluminum center (see above). A similar issue was found for the ligand OC­(C_5_F_10_)­(C_6_F_5_) (abbreviated OR), where synthetic attempts to the homoleptic [Al­(OR)_4_]^−^ led to the formation of [FAl­(OR)_3_]^−^ and the epoxide C_12_F_14_O.^46^ Due to the reluctance of the fourth ligand to coordinate to the aluminum center and the apparent stability of the fluoride adduct [FAl­(OTe^R^)_3_]^−^, our objective shifted to the selective synthesis of this species for its use as a WCA. In fact, related species such as [FAl­(OC­(CF_3_)_3_)_3_]^−^, the bulky [FAl­(OC­(C_6_F_5_)_3_)_3_]^−^ or the metal amide [FAl­(N­(C_6_F_5_)_2_)_3_]^−^ are described in the literature to behave as suitable WCAs.^12,13,30^

**Scheme 1:**
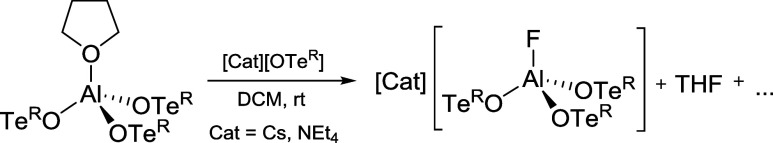
Reaction of **1**·THF with [OTe^R^]^−^ to Form the [FAl­(OTe^R^)_3_]^−^ Anion

Our synthetic approach relied on the same method previously applied in the synthesis of Lewis acid–base adducts of **1**, i.e., coordination of a fluoride to AlEt_3_ in the first step and subsequent protonation with HOTe^R^ (**2**). Based on a known literature procedure, the salt M­[FAlEt_3_] was prepared by reacting AlEt_3_ with MF (M = K, Cs).^47^ In a second step, the ethyl groups were protonated by **2** resulting in the formation of K­[FAl­(OTe^R^)_3_] (**5**) and Cs­[FAl­(OTe^R^)_3_] (**6**), respectively, which were isolated as colorless solids in quantitative yield (Figure 5). The formation of these compounds can easily be verified by NMR spectroscopy, as the resonance corresponding to the fluorine atom attached to aluminum displays a chemical shift of −178 ppm in the ^19^F NMR spectrum, being comparable to those found in [Li­(THF)_2_]­[FAl­(OC­(C_6_F_5_)_3_)_3_] (−177.3 ppm)^12^ and [Li­(DMC)_2_]­[FAl­(OC­(CF_3_)_3_)_3_] (−186.2 ppm).^30^

**Figure 5:**
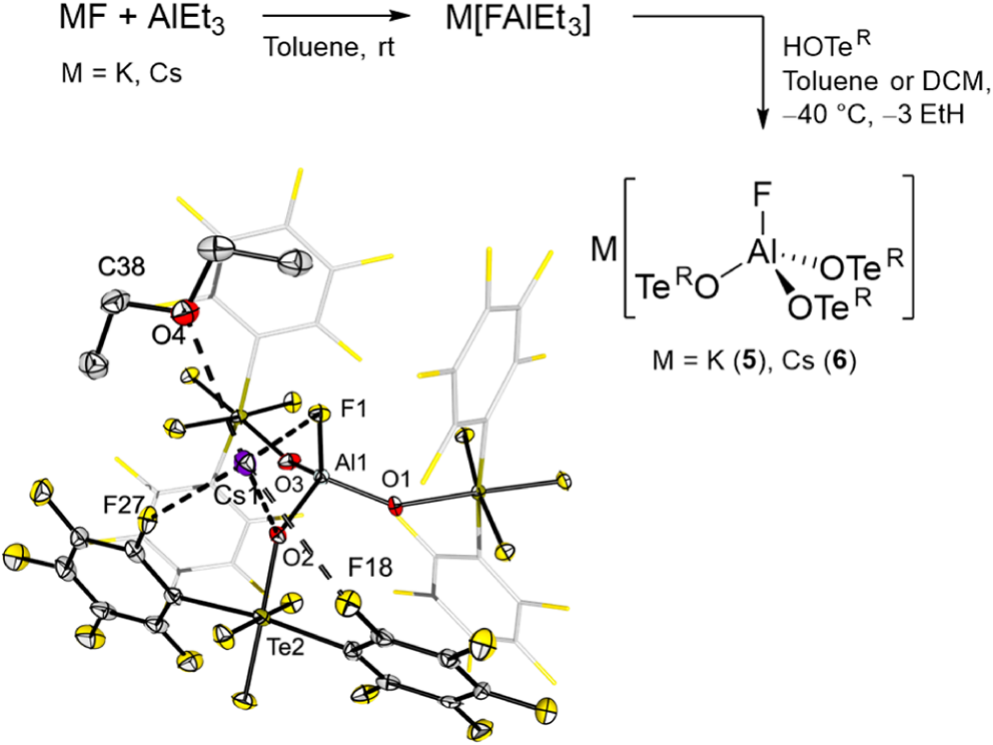
Synthesis of aluminates M­[FAl­(OTe^R^)_3_] (M = K (**5**), Cs (**6**)), and molecular structure in the solid state of **6·Et_2_O**. Displacement ellipsoids set at 50% probability. Selected bond lengths [pm]: Al1–O1 173.1(4), Al1–O2 175.3(4), Al1–O3 173.6(4), Al1–F1 167.7(3) Cs1···F1 312.1(3), Cs1···F27 330.1(3), Cs1···F18 336.9(3), Cs1···O2 318.2(4), Cs1···O4 325.0(4).

Both the potassium and the cesium salts are air-stable compounds with a remarkable high thermal stability up to 300 °C, as determined by thermogravimetric analysis for compound **5** (see Figure S41). They are poorly soluble in apolar solvents like CH_2_Cl_2_ or toluene but highly soluble in coordinating solvents like acetonitrile, THF or diethyl ether. Single crystals suitable for X-ray diffraction could be obtained for both salts by slow evaporation of a diethyl ether solution in air. Due to the high disorder of the ligands in the solid-state structure of the potassium salt (**5**, see Figure S43), only the structure of the cesium salt will be discussed here (Figure 5).

In the molecular structure, the aluminum center of **6** is tetrahedrally coordinated by three OTe^R^ ligands (Al–O bond lengths of 173.1(4) to 175.3(4) pm) and a fluoride with an Al–F bond length of 167.7(3) pm. The structure reveals coordination of a diethyl ether molecule to the cesium cation (*d* = 325.0(4) pm), which also interacts with the fluorine atom attached to the aluminum center (*d*(Cs1–F1) = 312.1(3) pm). Additionally, the crystal structure shows a metal–oxygen contact between cesium and the oxygen atom of one of the ligands (318.2(4) pm) along with several cesium–fluorine interactions involving fluorine atoms bonded to carbon or tellurium (Cs···F between 312.1(3) and 380.7(3) pm).

The ability of K­[FAl­(OTe^R^)_3_] (**5**) to undergo a metathesis reaction driven by the precipitation of poorly soluble potassium halides was then demonstrated by reaction with [NEt_3_Me]Cl (Figure 6). This reaction occurs with preservation of the anion and cleanly produces [NEt_3_Me]­[FAl­(OTe^R^)_3_] (**7**), which could be crystallized out of a CH_2_Cl_2_ solution. The solid-state structure shows that the cation–anion interaction is not a prominent feature as in the structure of **6**. Nevertheless, despite the apparent lack of interaction of the fluorido ligand with the cation, the Al–F distance is virtually the same compared to that found in the structure of the cesium salt **6** (166.9(8) pm vs 167.7(3) pm). The Al–O bond lengths (172.9(8) to 175.4(7) pm) are similar to those found in compound **6**.

**Figure 6:**
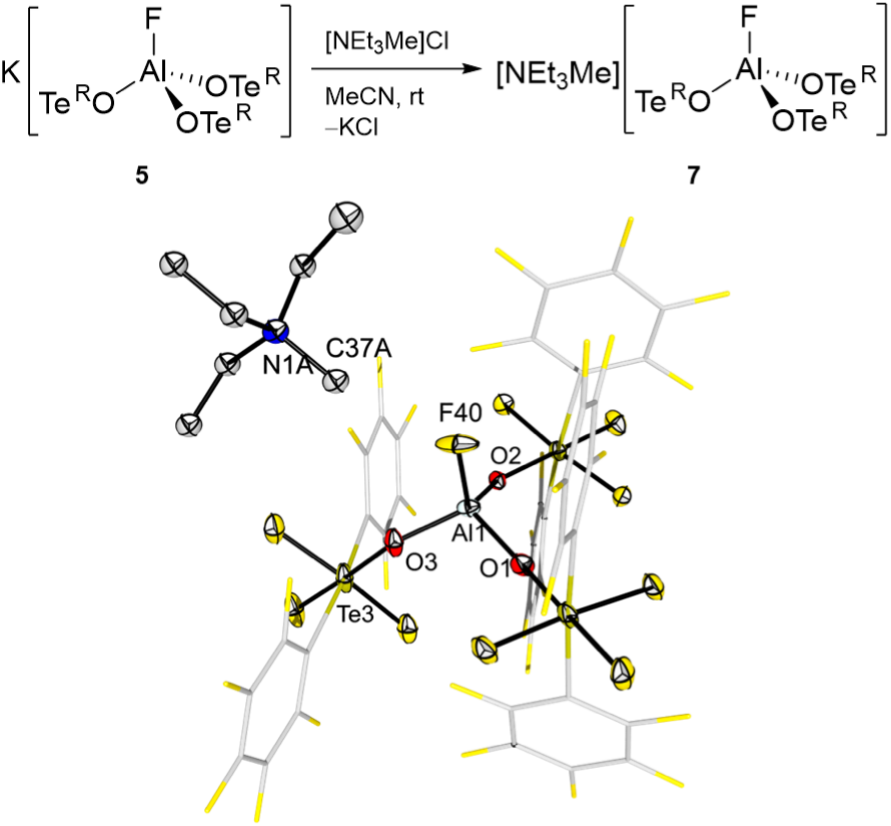
Metathesis reaction of the potassium salt **5** with [NEt_3_Me]Cl and molecular structure in the solid state of [NEt_3_Me]­[FAl­(OTe^R^)_3_] (**7**). Displacement ellipsoids set at 50% probability. Selected bond lengths [pm]: Al1–O1 172.9(8), Al1–O2 175.4(7), Al1–O3 172.9(8), Al1–F40 166.9(8).

The high accumulation of negative charge in the fluorido ligand bound to the Al center makes it the most exposed site of the WCA, rendering it potentially less effective at stabilizing reactive cations. Its high susceptibility to ion pairing is evident, as shown in the solid-state structure of **6**. On the other hand, in special cases, ion pairing may be beneficial due to e.g., better crystallization behavior of the salt.^30^ Nevertheless, to improve the weakly coordinating ability of the anion, we considered the substitution of the fluoride by a teflate ligand. In this regard, OTeF_5_ is not as sterically demanding as OTe^R^, but is able to delocalize the negative charge even better than a single fluoride and is known to perform well in the formation of WCAs.^19,21^ Gratifyingly, it was possible to add a teflate instead of a fluorido ligand to the Al center by using a source of free [OTeF_5_]^−^. As a first approach, the ammonium salt [NEt_4_]­[(F_5_TeO)­Al­(OTe^R^)_3_] (**8**) was targeted, which could be synthesized in two different ways (Figure 7). The first route consists in adding AlEt_3_ to a mixture of [NEt_4_]­[OTeF_5_] and three equivalents of HOTe^R^ (**2**) at −40 °C and slowly warming the mixture to room temperature. Presumably, [OTeF_5_]^−^ coordinates first to AlEt_3_, with the ethyl groups being protonated by **2** in a subsequent step. The second method involves the reaction of the adduct **1**·THF with the salt [NEt_4_]­[OTeF_5_] in DCM, whereby THF is replaced by an OTeF_5_ group to yield **8**. Most importantly, this second method enabled also the generation of the corresponding silver salt Ag­[(F_5_TeO)­Al­(OTe^R^)_3_] (**9**) by employing AgOTeF_5_ instead of [NEt_4_]­[OTeF_5_] (Figure 7). The presence of an Ag^+^ cation renders this compound synthetically useful, since silver salts of WCAs generally serve as potent reagents in halide abstraction reactions to generate reactive cations.^20–22^ In the ^19^F NMR spectra of both salts, signals corresponding to two distinct ligands bound to the Al center are visible. At around −9 and 20 ppm, the characteristic triplet of quintets and doublet of quintets for the Te-bound fluorine atoms in the OTe^R^ ligands are observed. Adjacent to these, the characteristic AB_4_ spin system of the fluorine atoms of an OTeF_5_ group at around −37 ppm and −48 ppm evidence the presence of this ligand.^19^ The integrals of the signals of the different fluorine atoms of −OTe^R^ and −OTeF_5_ lead to a ligand ratio of 3:1, confirming the formation of the heteroleptic anion [(F_5_TeO)­Al­(OTe^R^)_3_]^−^.

**Figure 7:**
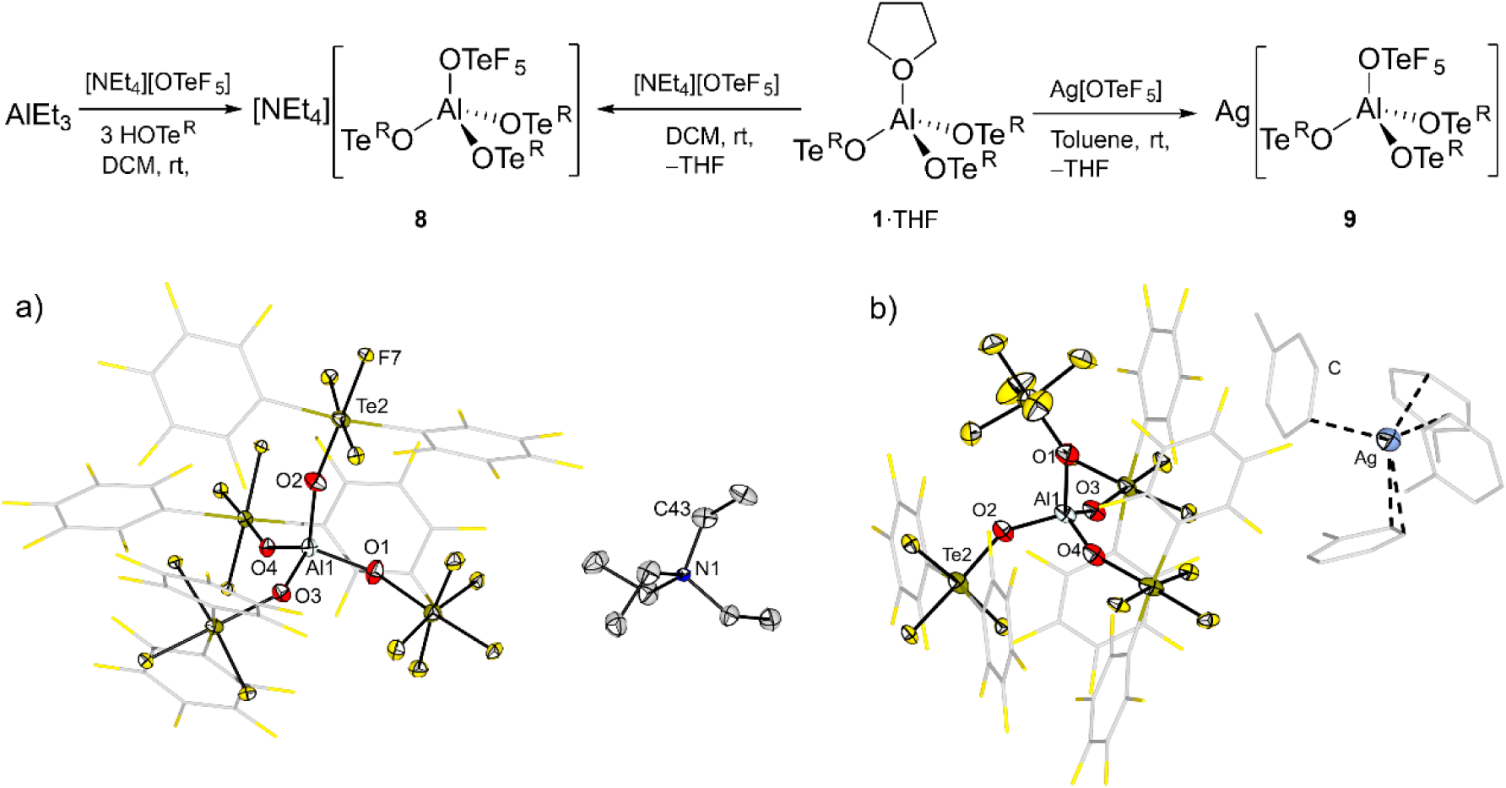
Synthesis of the tetraethylammonium (**8**) and silver salts (**9**) of the weakly coordinating anion [(F_5_TeO)­Al­(OTe^R^)_3_]^−^. (a) Molecular structure in the solid state of [NEt_4_]­[(F_5_TeO)­Al­(OTe^R^)_3_] (**8**). Displacement ellipsoids set at 50% probability. Selected bond lengths [pm]: Al1–O1 176.6(3), Al1–O2 172.4(3), Al1–O3 173.4(3), Al1–O4 173.7(3). (b) Molecular structure in the solid state of Ag­[(F_5_TeO)­Al­(OTe^R^)_3_] (**9**). Displacement ellipsoids set at 50% probability. Selected bond lengths [pm]: Al1–O1 177.0(5), Al1–O2 173.0(5), Al1–O3 172.1(5), Al1–O4 173.2(5).

Both salts of this new WCA were successfully crystallized. The ammonium salt **8** crystallizes in the monoclinic space group C2/*c*, and the solid-state structure reveals a distorted tetrahedral coordination around the Al center (Figure 7a). Notably, the Al1–O1 bond corresponding to the OTeF_5_ group is the longest, with 176.6(3) pm, which is slightly longer compared to the Al–O bonds of the OTe^R^ ligands (172.4(3) to 173.7(3) pm). In the case of the silver salt **9** (Figure 7b), the solid-state structure shows that the anion and cation are well separated, with the anion adopting virtually the same structure as in the case of the ammonium salt **8**. The silver cation is coordinated by four toluene molecules, three of them with a η^1^ hapticity, while the fourth one is η^2^. This underlines the weakly coordinating ability of the anion, since the Ag^+^ cation interacts preferably with the weakly basic solvent toluene, leading to a solid-state structure like this one (Figure 7b), which would be otherwise unattainable.^48^

As an example of the synthetic possibilities of silver salt **9**, we subsequently employed it for the preparation of a potentially strong Brønsted acid, since these species also play a crucial role in the generation of reactive cations.^49–51^ Utilizing the reactivity of silver salt **9**, its reaction with HCl in toluene or mesitylene should lead to such a species. Indeed, upon addition of gaseous HCl to a solution of **9** in toluene, an immediate precipitation of a white solid occurred indicating the formation of AgCl, accompanied by a color change of the solution to red due to the protonation of the aromatic solvent (Scheme 2). The reaction mixture was quenched by addition of Et_2_O, which resulted in an immediate loss of color. The ^19^F NMR spectrum of the reaction mixture exhibited no significant changes, indicating the stability of the anion during the process. However, in the ^1^H NMR spectrum, in addition to signals for the ethyl groups of the diethyl ether molecule, a signal at around 16 ppm was observed, which corresponds to [H­(Et_2_O)_2_]+^52^ and therefore shows the capability of the formed Brønsted acid to protonate Et_2_O (Scheme 2).

**Scheme 2:**
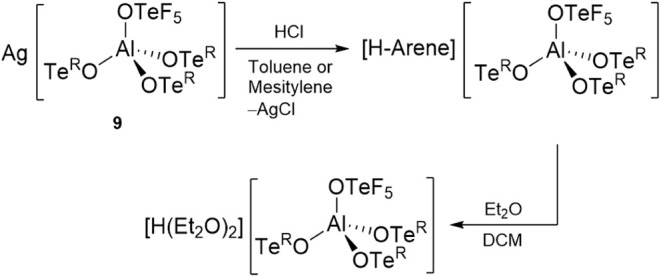
Reactivity of the Silver Salt **9** with HCl in an Aromatic Solvent to Form a Brønsted Acid and Subsequent Protonation of Et_2_O

To investigate the acid strength of the Brønsted acid in *ortho*-difluorobenzene, mesityl oxide was added to the acidic reaction mixture. In this approach, the chemical shift difference (Δδ) between the resonances of C_α_ and C_β_ in the ^13^C NMR spectrum of mesityl oxide upon protonation is determined.^53^ For the new acid, a value of 81.0 ppm was observed, which is slightly lower than that obtained for H­[Al­(OTeF_5_)_4_] (87.9 ppm), yet significantly higher than those obtained for HOTeF_5_ (46.6 ppm) and CF_3_SO_3_H (70.3 ppm) in *ortho*-difluorobenzene.^10^

It was also possible to react the silver salt **9** with Ph_3_CCl and stabilize the synthetically valuable carbocation [Ph_3_C]^+^ upon formation of AgCl (Figure 8). The solid-state structure of [Ph_3_C]­[(F_5_TeO)­Al­(OTe^R^)_3_] (**10**) shows the anion and cation well separated. Interestingly, in contrast to [Ph_3_C]­[Al­(OTeF_5_)_4_],^10^ there is no contact between the central carbenium ion and a fluorine atom of the anion, underlining the weaker coordination of the new mixed anion.

**Figure 8:**
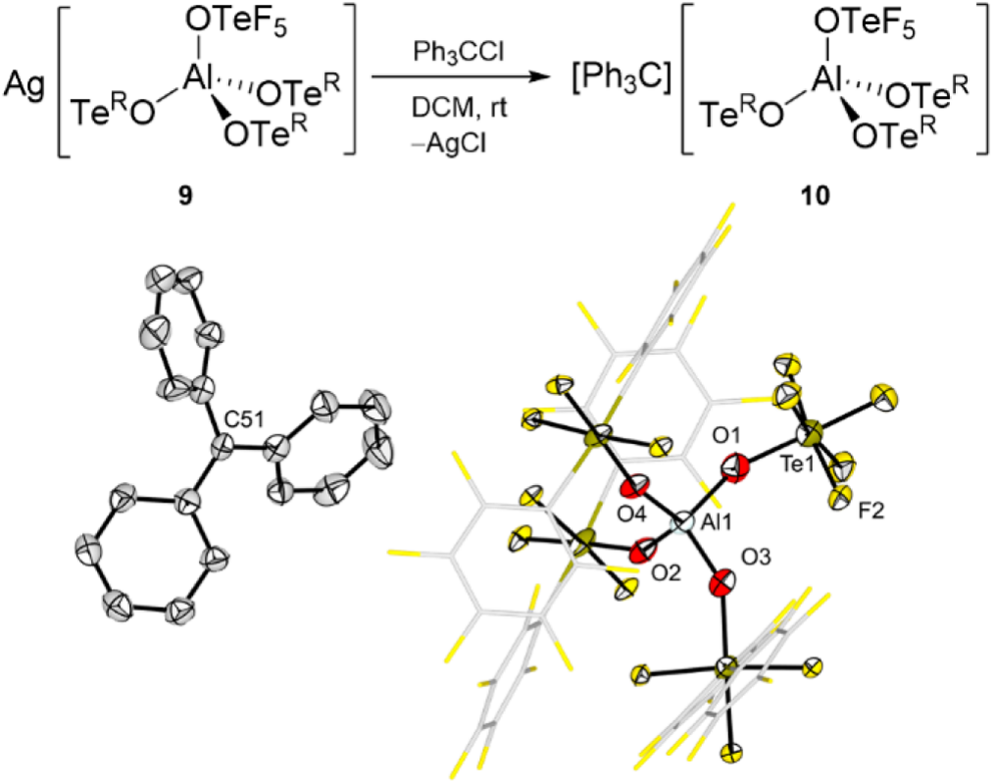
Metathesis reaction of the silver salt **9** with Ph_3_CCl and molecular structure in the solid state of [Ph_3_C]­[(F_5_TeO)­Al­(OTe^R^)_3_] (**10**). Displacement ellipsoids set at 50% probability. Selected bond lengths [pm]: Al1–O1 176.1(7), Al1–O2 173.8(7), Al1–O3 173.7(6), Al1–O4 172.2(6).

Finally, to evaluate the coordination ability of the WCAs discussed in this paper, their electrostatic potential surfaces were calculated (BP86-D3­(BJ)/def-SV­(P)). Figure 9 shows the projection of the electrostatic potential onto a 0.025 e^–^ Bohr^–3^ isodensity surface. Herein, a red color indicates a high accumulation of negative charge. In case of the [FAl­(OTe^R^)_3_]^−^ anion, the F atom attached to Al carries a significant negative charge, which is in agreement with its described tendency to ion pairing. The mixed [(F_5_TeO)­Al­(OTe^R^)_3_]^−^ anion should be less coordinating, since the negative charge is delocalized over 44 fluorine atoms. Also, the oxygen atoms, which might be prone to attacks by electrophiles, are significantly better shielded compared to those in [Al­(OTeF_5_)_4_]^−^ and [FAl­(OTe^R^)_3_]^−^, making [(F_5_TeO)­Al­(OTe^R^)_3_]^−^ potentially more resistant against ligand abstraction.

**Figure 9:**
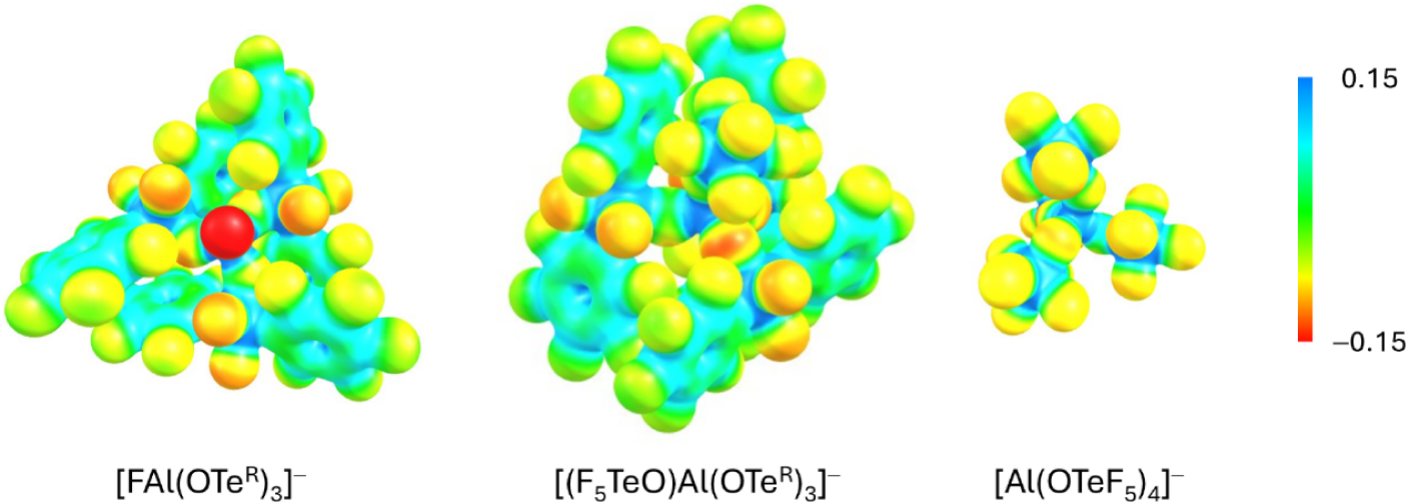
Comparison of the electrostatic potential calculated at the BP86-D3­(BJ)/def-SV­(P) level, mapped onto a 0.025 e^–^ Bohr^–3^ isodensity surface, of the discussed WCAs, with previously known [Al­(OTeF_5_)_4_]^−^.

## Conclusion

In summary, by making use of the sterically demanding OTeF_3_(C_6_F_5_)_2_ ligand (OTe^R^), we explored the properties of the Lewis acid Al­(OTe^R^)_3_ (**1**). As evidenced by the Gutmann–Beckett analysis and FIA calculations, **1** can in principle be classified as a Lewis superacid with a remarkable acidity comparable to that of Al­(OC­(CF_3_)_3_)_3_ or Al­(OC­(C_6_F_5_)_3_)_3_. While stabilization as acid–base adducts with THF and dimethyl carbonate was achieved, yielding compounds **1**·THF and **1**·DMC, attempts to isolate the free Lewis acid **1** were hindered by its intrinsic reactivity. Computational studies further revealed the tendency of **1** toward fluoride abstraction from its own ligands. In fact, the synthesis of **1**·THF and **1**·DMC was only successful starting from AlEt_3_ and the corresponding base, and subsequent protonation with HOTe^R^ to prevent fluoride abstraction from the superacidic Al center.

Nevertheless, taking the high Lewis acidity of **1** as an advantage, different weakly coordinating anions were synthesized. Potassium (**5**) and cesium (**6**) salts of the fluoride adduct [FAl­(OTe^R^)_3_]^−^ could be isolated in pure form and exhibit remarkable thermal stability up to 300 °C. The potassium salt **5** was employed in a metathesis reaction with [NEt_3_Me]Cl to yield [NEt_3_Me]­[FAl­(OTe^R^)_3_] (**7**). To improve the delocalization of the negative charge in the WCA, salts of the even more weakly coordinating mixed anion [(F_5_TeO)­Al­(OTe^R^)_3_]^−^ with cations [NEt_4_]^+^ (**8**) and Ag^+^ (**9**) were obtained via reactions of **1**·THF with the corresponding teflate salts [NEt_4_]­[OTeF_5_] and AgOTeF_5_. The silver salt **9** proved to be synthetically valuable due to the precipitation of silver halides and formation of a strong Brønsted acid as well as the [Ph_3_C]^+^ cation. The weakly coordinating nature of the mixed anion was demonstrated by the preferred coordination of toluene to Ag^+^ in the crystal structure of **9·4 toluene**. In agreement with this, the electrostatic potential surface analysis confirmed superior charge delocalization and enhanced oxygen shielding of [(F_5_TeO)­Al­(OTe^R^)_3_]^−^ compared to [FAl­(OTe^R^)_3_]^−^ and [Al­(OTeF_5_)_4_]^−^. These properties are combined with superior crystallization behavior due to a reduced tendency to structural disorder, making solid-state structure analysis easier. These findings do not only expand the family of Lewis superacids but also provide new insights into the design of weakly coordinating anions, which are now available for potential applications in the stabilization of reactive cations.

## Supplementary Material



## Notes

Detailed experimental procedures, characterization data, computational details (PDF)

## †

Institut für Chemie, Humboldt-Universität zu Berlin, Brook-Taylor-Straße 2, 12489 Berlin, Germany
