# Supplementary material for: An Aluminum-Based Lewis Superacid and Its Weakly Coordinating Anions Derived from an Organotellurium Ligand
Source: JACS Au. 2025 Jun 23;5(7):3565–74. doi: 10.1021/jacsau.5c00577 (PMC12308415; doi:10.1021/jacsau.5c00577)
Supplement: Supplementary file 1 [file au5c00577_si_001.pdf]

Supporting Information

for

An Aluminum-based Lewis Superacid and its Weakly Coordinating Anions Derived from an Organotellurium Ligand

Daniel Wegener,[a] Niklas Limberg,[a] Moritz Bubenik,[a] Alberto Pérez-Bitrián,[a,b] Anja Wiesner,[a] Sebastian Riedel*[a]

[a] Fachbereich Biologie, Chemie, Pharmazie
Institut für Chemie und Biochemie – Anorganische Chemie
Freie Universität Berlin
Fabeckstraße 34/36, 14195 Berlin (Germany)

E-Mail: s.riedel@fu-berlin.de

[b] Current address:
Institut für Chemie
Humboldt-Universität zu Berlin
Brook-Taylor-Straße 2, 12489 Berlin (Germany)

Table of contents

1. Experimental section S2
2. NMR spectra S13
3. Thermogravimetric analysis S34
4. Crystal data S35
5. Powder diffraction data S39
6. Quantum-chemical calculations S40
7. References S57

1 Experimental section

General procedures and materials

Unless otherwise mentioned, all experiments were performed under exclusion of moisture and oxygen using standard Schlenk techniques.[1] Solids were handled in a MBRAUN UNIlab plus glovebox under an argon atmosphere (O_2 < 0.5 ppm, H_2O < 0.5 ppm). Solvents were dried using a MBraun SPS-800 solvent system (CH_2Cl_2, MeCN, *n*-pentane), or with potassium (THF) before use and stored over 3 or 4 Å molecular sieves. K[$FAlEt_3$],[2] and $HOTeF_3(C_6F_5)_2$ ($HOTe^R$),[3] were prepared according to literature procedures. All other reagents were purchased from standard commercial suppliers and used as received. NMR spectra were recorded on a JEOL 400 MHz ECS or a JEOL 400 MHz ECZ spectrometer. All reported chemical shifts (δ in ppm) are referenced to the Ξ values given in the IUPAC recommendations of 2008 using the ^2H signal of the deuterated solvent as internal reference.[4] Multiplicity is indicated as follows: s = singlet, t = triplet, quart = quartet, quint = quintet, sept = septet, dd = doublet of doublets, dt = doublet of triplets, dquint = doublet of quintets, tquint = triplet of quintets, m = multiplet. IR spectra were measured on a Bruker ALPHA FTIR spectrometer with a diamond ATR inside a glovebox under an argon atmosphere at room temperature. The ESI-TOF mass spectrometry measurements were performed on an Agilent 6210 ESI-TOF, Agilent Technologies, Santa Clara, CA, USA. Solvent flow rate was adjusted to 4 µL/min, spray voltage set to 4 kV. Drying gas flow rate was set to 15 psi (1 bar). Elemental analyses (CHNS) were carried out using a VARIO EL elemental analyzer. Crystal data were collected with MoKα radiation on a Bruker D8 Venture diffractometer with a CMOS area detector. Single crystals were picked at −40 °C under nitrogen atmosphere and mounted on a 0.15 mm Mitegen micromount using perfluoroether oil. The structures were solved with the ShelXT[5] structure solution program using intrinsic phasing and refined with the ShelXL[6] refinement package using least squares minimizations by using OLEX2.[7] For visualization the program Diamond V4.6.4 was used.[8] CCDC 2448090 until 2448098 and 2454862 contain the supplementary crystallographic data for this paper. These data are provided free of charge by The Cambridge Crystallographic Data Centre. Crystal data and other details of the structure analyses are summarized in Table S1. Suitable crystals for X-ray diffraction studies were obtained as indicated in the corresponding experimental entry (*vide infra*). Powder X-ray diffractograms were measured using Cu_{Ka} radiation via a 360° Phi scan with an exposure time of 120 s. The data were integrated using APEX5

and analyzed using DIFFRAC.EVA. Thermogravimetric analyses (TGA) were performed using a STA 449 F3 JUPITER device at a heating rate of 10 °C min^{-1} under an inert atmosphere. Spectra were processed using NETZSCH Proteus.

Synthesis of $Al(OTe^R)_3$·THF (**1**·THF)

THF (90 µL, 1.10 mmol, 1 eq) was added to a solution of $AlEt_3$ (122 mg, 1.07 mmol, 1 eq) in toluene (5 mL) to prepare a stock solution. A portion of this solution (0.50 mL) was added dropwise to a suspension of $HOTe^R$ (170 mg, 0.32 mmol, 3 eq) in toluene (5 mL) at –45 °C. The reaction mixture was allowed to reach room temperature overnight and after no more gas evolution was observed, the solvent was removed under reduced pressure. The solid was washed with *n*-pentane (3 × 3 mL) to afford a colorless product, which was identified as compound **1**·THF (175 mg, 0.10 mmol, 96%).

^{1}H NMR (400 MHz, CD_2Cl_2, 22 °C): δ = 4.54 (m, 4H, CH_2OCH_2), 2.30 (m, 4H, CH_2CH_2) ppm.

^{19}F NMR (377 MHz, CD_2Cl_2, 22 °C): δ = 9.8 (tquint, 1F, $^4J(^{19}F,^{19}F_o)$ = 19 Hz, $^2J(^{19}F,^{19}F)$ = 65 Hz, $^1J(^{125}Te,^{19}F)$ = 2950 Hz), −21.0 (dquint, 2F, $^4J(^{19}F,^{19}F_o)$ = 21 Hz, $^1J(^{125}Te,^{19}F)$ = 2850 Hz), −131.1 (m, 4F, $^3J(^{19}F_o,^{19}F_m)$ = 20 Hz, *o*-F), −146.4 (m, 2F, $^4J(^{19}F_o,^{19}F_p)$ = 6 Hz, *p*-F), −159.7 (m, 4F, $^3J(^{19}F_p,^{19}F_m)$ = 19 Hz, *m*-F) ppm.

^{27}Al NMR (128 MHz, CD_2Cl_2, 22 °C): δ = 44.1 (br s) ppm.

IR (ATR, 25°C): $\tilde{\nu}$ = 3486 (w), 2930 (w), 1639 (m), 1516 (s), 1484 (s, C_6F_5-Ring), 1396 (m), 1289 (m), 1089 (s, C–F), 979 (s, C–F), 906 (Al–O), 810 (m, C_6F_5-Ring), 725 (w), 641 (s, Te–F), 604 (s), 492 (m) cm^{-1}.

Elemental analysis calcd (%): C 28.2 H 0.47; found: C 28.8 H 0.6

Synthesis of $Al(OTe^R)_3$·DMC (**1**·DMC)

Dimethylcarbonate (100 µL, 1.10 mmol, 1 eq) was added to a solution of $AlEt_3$ (128 mg, 1.12 mmol, 1 eq) in DCM (7 mL) to prepare a stock solution. A portion of this solution (0.50 mL) was added dropwise to a suspension of $HOTe^R$ (130 mg, 0.24 mmol, 3 eq) in DCM (5 mL) at –45 °C. The reaction mixture was allowed to reach room temperature over 4 h and stirred additionally 1 h at room temperature. The solvent was removed under reduced pressure to afford a colourless product, which was identified as compound **1**·DMC (120 mg, 0.07 mmol, 86%).

Single crystals suitable for X-ray diffraction were obtained by slow diffusion of *n*-pentane into a CH_2Cl_2 solution of **2** at –24 °C.

^{1}H NMR (400 MHz, CD_2Cl_2, 22 °C): δ = 4.33 (m, OCH_3) ppm.

^{19}F NMR (377 MHz, CD_2Cl_2, 22 °C): δ = 8.1 (tquint, 1F, $^4J(^{19}F,^{19}F_o)$ = 19 Hz, $^2J(^{19}F,^{19}F)$ = 72 Hz, $^1J(^{125}Te,^{19}F)$ = 2950 Hz), −22.4 (dquint, 2F, $^4J(^{19}F,^{19}F_o)$ = 20 Hz, $^1J(^{125}Te,^{19}F)$ = 2840 Hz), −130.7 (m, 4F, $^3J(^{19}F_o,^{19}F_m)$ = 19 Hz, *o*-F), −146.8 (m, 2F, $^4J(^{19}F_o,^{19}F_p)$ = 5 Hz, *p*-F), −160.1 (m, 4F, $^3J(^{19}F_p,^{19}F_m)$ = 20 Hz, *m*-F) ppm.

^{27}Al NMR (128 MHz, CD_2Cl_2, 22 °C): δ = 44.5 (br s) ppm.

Synthesis of $Al(OTe^R)_3 \cdot OPEt_3$ (**1**·$OPEt_3$)

Method 1:

Et_3PO (108 mg, 0.81 mmol, 1 eq) was added to a solution of $AlEt_3$ (92 mg, 0.81 mmol, 1 eq) in DCM (8 mL) to prepare a stock solution and cooled to –45 °C. A portion of this solution (1 mL) was added to a solution of $HOTe^R$ (162 mg, 0.30 mmol, 3 eq) in DCM (5 mL) at –45 °C. The reaction mixture was warmed to room temperature overnight and afterwards the solvent was removed under reduced pressure. The obtained product was washed with *n*-pentane (3 × 3 mL) and identified as **1**·$OPEt_3$ (160 mg, 0.09 mmol, 90%).

Single crystals suitable for X-ray diffraction were obtained by slow diffusion of *n*-pentane into a CH_2Cl_2 solution of **1**·$OPEt_3$.

Method 2:

In a Young NMR tube, **1**·THF (40 mg, 0.02 mmol, 1 eq) was dissolved in DCM (0.5 mL) and a solution of $OPEt_3$ (3 mg, 0.02 mmol, 1 eq) in DCM (0.3 mL) was added. After shaking the reaction mixture for 10 min, all volatiles were removed under reduced pressure and the product was redissolved in DCM to be identified as **1**·$OPEt_3$.

^{1}H NMR (400 MHz, CD_2Cl_2, 22 °C): δ = 1.96 (dquart, 6H, $^2J(^{31}P,^1H)$ = 12.8 Hz, $^3J(^1H,^1H)$ = 8.1 Hz, CH_2), 1.09 (dt, 9H, $^3J(^{31}P,^1H)$ = 18.6 Hz, $^3J(^1H,^1H)$ = 7.0 Hz, CH_3) ppm.

^{19}F NMR (377 MHz, CD_2Cl_2, 22 °C): δ = 10.8 (tquint, 1F, $^4J(^{19}F,^{19}F_o)$ = 20 Hz, $^2J(^{19}F,^{19}F)$ = 72 Hz, $^1J(^{125}Te,^{19}F)$ = 2920 Hz), −22.3 (dquint, 2F, $^4J(^{19}F,^{19}F_o)$ = 22 Hz, $^1J(^{125}Te,^{19}F)$ = 2822 Hz), −130.8 (m, 4F, $^3J(^{19}F_o,^{19}F_m)$ = 18 Hz, *o*-F), −147.7 (m, 2F, $^4J(^{19}F_o,^{19}F_p)$ = 6 Hz, *p*-F), −160.8 (m, 4F, $^3J(^{19}F_p,^{19}F_m)$ = 19 Hz, *m*-F) ppm.

^{27}Al NMR (128 MHz, CD_2Cl_2, 22 °C): δ = 39.0 (br s) ppm.

^{31}P{^{1}H} NMR (104 MHz, CD_2Cl_2, 22 °C): δ = 77.8 (s) ppm.

IR (ATR, 25°C): $\tilde{\nu}$ = 2930 (w), 1637 (m), 1516 (s), 1486 (s, C_6F_5-Ring), 1394 (m), 1287 (m), 1089 (s, C–F), 981 (s, C–F), 871 (Al–O), 781 (m, C_6F_5-Ring), 723 (w), 634 (s, Te–F), 594 (s), 494 (m) cm^{-1}.

Synthesis of $AlEt[OTe^R]_2 \cdot OPPh_3$ (**3**)

Ph_3PO (120 mg, 0.43 mmol, 1 eq) was added to a solution of $AlEt_3$ (49 mg, 0.43 mmol, 1 eq) in DCM (4 mL) to prepare a stock solution and cooled to –45 °C. A portion of this solution (1 mL) was added to a solution of $HOTe^R$ (114 mg, 0.21 mmol, 2 eq) in CH_2Cl_2 (5 mL) at –45 °C. The reaction mixture was warmed to room temperature overnight and the solvent was removed under reduced pressure. The obtained product was washed with *n*-pentane (3 × 3 mL) and identified as **3** (140 mg, 0.10 mmol, 95%).

Single crystals suitable for X-ray diffraction were obtained by slow diffusion of *n*-pentane into a CH_2Cl_2 solution of **3**.

^{1}H NMR (400 MHz, CD_2Cl_2, 22 °C): δ = 7.78-7.72 (m, 1H, Ar-*H*), 7.6-7.5 (m, 4H, Ar-*H*), 0.50 (t, 3H, $^3J(^1H,^1H)$ = 8.0 Hz, CH_3), –0.48 (q, 2H, CH_2) ppm.

^{19}F NMR (377 MHz, CD_2Cl_2, 22 °C): δ = 13.7 (tquint, 1F, $^4J(^{19}F,^{19}F_o)$ = 20 Hz, $^2J(^{19}F,^{19}F)$ = 79 Hz, $^1J(^{125}Te,^{19}F)$ = 2930 Hz), −21.5 (dquint, 2F, $^4J(^{19}F,^{19}F_o)$ = 21 Hz, $^1J(^{125}Te,^{19}F)$ = 2780 Hz), −128.9 (m, 4F, $^3J(^{19}F_o,^{19}F_m)$ = 18 Hz, *o*-F), −147.3 (m, 2F, $^4J(^{19}F_o,^{19}F_p)$ = 7 Hz, *p*-F), −159.5 (m, 4F, $^3J(^{19}F_p,^{19}F_m)$ = 19 Hz, *m*-F) ppm.

^{27}Al NMR (128 MHz, CD_2Cl_2, 22 °C): δ = 57.0 (br s) ppm.

^{31}P{^{1}H} NMR (104 MHz, CD_2Cl_2, 22 °C): δ = 43.8 (s) ppm.

IR (ATR, 25°C): $\tilde{\nu}$ = 2932 (w), 2870 (w), 1638 (m), 1516 (s), 1490 (s, C_6F_5-Ring), 1440 (m), 1393 (m), 1290 (m), 1170 (m), 1125 (m), 1090 (s, C–F), 981 (s, C–F), 884 (Al–O), 807 (m, C_6F_5-Ring), 726 (w), 691 (m), 631 (s, Te–F), 597 (m), 537 (s), 492 (m) cm^{-1}.

Synthesis of $GaEt[OTe^R]_2 \cdot OPEt_3$ (**4**)

Et_3PO (70 mg, 0.52 mmol, 1 eq) was added to a solution of $GaEt_3$ (81 mg, 0.52 mmol, 1 eq) in DCM (5 mL) to prepare a stock solution and cooled to 0 °C. Part of this solution (1 mL) was added to a solution of $HOTe^R$ (167 mg, 0.31 mmol, 3 eq) in DCM (5 mL) at

the same temperature. The reaction mixture was warmed to room temperature over a period of 2 h and then heated to 80 °C for half an hour. Afterwards the reaction mixture was characterized via NMR spectroscopy.

Single crystals suitable for X-ray diffraction were obtained by slow diffusion of *n*-pentane into a CH_2Cl_2 solution of **5**.

^{19}F NMR (377 MHz, CD_2Cl_2, 22 °C): δ = 12.9 (tquint, 1F, $^4J(^{19}F,^{19}F_o)$ = 19 Hz, $^2J(^{19}F,^{19}F)$ = 77 Hz, $^1J(^{125}Te,^{19}F)$ = 2820 Hz), −23.1 (dquint, 2F, $^4J(^{19}F,^{19}F_o)$ = 20 Hz, $^1J(^{125}Te,^{19}F)$ = 2740 Hz), −130.8 (m, 4F, $^3J(^{19}F_o,^{19}F_m)$ = 19 Hz, *o*-F), −149.5 (m, 2F, $^4J(^{19}F_o,^{19}F_p)$ = 7 Hz, *p*-F), −161.8 (m, 4F, $^3J(^{19}F_p,^{19}F_m)$ = 19 Hz, *m*-F) ppm.

^{31}P{^{1}H} NMR (104 MHz, CD_2Cl_2, 22 °C): δ = 73.5 (s) ppm.

Synthesis of K[FAl(OTe^R)$_3$] (**5**)

K[FAlEt$_3$] (32 mg, 0.19 mmol, 1eq) was dissolved in DCM (1 mL), cooled to –40 °C and added to a solution of HOTe^R (318 mg, 0.59 mmol, 3.1 eq) in DCM (8 mL) at the same temperature. The formed suspension was allowed to reach room temperature and afterwards all volatiles were removed under reduced pressure. The white crude product was washed with DCM (3 × 5 mL), dried *in vacuo* and identified as **5** (300 mg, 0.178 mmol, 93%).

Single crystals suitable for X-ray diffraction were obtained by slow evaporation of a diethyl ether solution of **5** in air.

^{13}C{^{19}F} NMR (100 MHz, CD_3CN, 22 °C): δ = 147.0 (s, *o*-C), 145.0 (s, *p*-C), 138.7 (s, *m*-C) ppm.

^{19}F NMR (377 MHz, CD_3CN, 22 °C): δ = 12.0 (tquint, 3F, $^4J(^{19}F,^{19}F_o)$ = 20 Hz, $^2J(^{19}F,^{19}F)$ = 80 Hz, $^1J(^{125}Te,^{19}F)$ = 2880 Hz), −22.3 (dquint, 6F, $^4J(^{19}F,^{19}F_o)$ = 20 Hz, $^1J(^{125}Te,^{19}F)$ = 2800 Hz), −130.2 (m, 12F, $^3J(^{19}F_o,^{19}F_m)$ = 19 Hz, *o*-F), −149.3 (m, 6F, $^4J(^{19}F_o,^{19}F_p)$ = 6 Hz, *p*-F), −161.7 (m, 12F, $^3J(^{19}F_p,^{19}F_m)$ = 19 Hz, *m*-F), −178.6 (s, 1F, Al–F) ppm.

^{27}Al NMR (128 MHz, CD_3CN, 22 °C): δ = 47.1 (br s) ppm.

^{125}Te NMR (126 MHz, CD_2Cl_2, 22 °C): δ = 711 (dtm, $^1J(^{19}F, ^{125}Te)$ = 2850 Hz, $^1J(^{19}F, ^{125}Te)$ = 2730 Hz) ppm.

MS (ESI+): *m/z*: 129.9 Te^+, 1728.5 $\{K_2[FAl(OTeR)_3]\}^+$.

MS (ESI−): *m/z*: 1070.7 $[H(OTe^R)_2]^-$, 536.9 $[OTe^R]^-$.

IR (ATR, 25°C): $\tilde{\nu}$ = 1640 (m), 1520 (s), 1490 (s, C_6F_5-Ring), 1393 (m), 1290 (m), 1090 (s, C–F), 981 (s, C–F), 921 (m), 870 (Al–O), 811 (m, C_6F_5-Ring), 751 (m), 631 (s, Te–F), 524 (m), 496 (m) cm^{-1}.

Elemental analysis calcd (%): C 25.6; found: C 25.7

Synthesis of $Cs[FAl(OTe^R)_3]$ (**6**)

$Cs[FAlEt_3]$ (40 mg, 0.15 mmol, 1eq) was dissolved in DCM (1 mL), cooled to −40 °C and added to a solution of $HOTe^R$ (251 mg, 0.47 mmol, 3.1 eq) in DCM (8 mL) at the same temperature. The formed suspension was allowed to reach room temperature and afterwards all volatiles were removed under reduced pressure. The white crude product was washed with DCM (3 × 5 mL), dried *in vacuo* and identified as **6** (250 mg, 0.140 mg, 94%).

Single crystals suitable for X-ray diffraction were grown from a saturated diethyl ether solution of **6** at room temperature.

^{19}F NMR (377 MHz, CD_3CN, 22 °C): δ = 11.7 (tquint, 3F, $^4J(^{19}F,^{19}F_o)$ = 20 Hz, $^2J(^{19}F,^{19}F)$ = 78 Hz, $^1J(^{125}Te,^{19}F)$ = 2860 Hz), −22.5 (dquint, 6F, $^4J(^{19}F,^{19}F_o)$ = 20 Hz, $^1J(^{125}Te,^{19}F)$ = 2780 Hz), −130.5 (m, 12F, $^3J(^{19}F_o,^{19}F_m)$ = 19 Hz, *o*-F), −149.7 (m, 6F, $^4J(^{19}F_o,^{19}F_p)$ = 6 Hz, *p*-F), −162.1 (m, 12F, $^3J(^{19}F_p,^{19}F_m)$ = 19 Hz, *m*-F), −178.7 (s, 1F, Al–F) ppm.

^{27}Al NMR (128 MHz, CD_3CN, 22 °C): δ = 46.7 (br s) ppm.

MS (ESI+): *m/z*: 132.9 Cs^+.

MS (ESI−): *m/z*: 1650.6 $[FAl(OTe^R)_3]^-$, 1070.7 $[H(OTe^R)_2]^-$, 536.9 $[OTe^R]^-$.

Synthesis of $[NEt_3Me][FAl(OTe^R)_3]$ (**7**)

$K[FAl(OTe^R)_3]$ (70 mg, 0.04 mmol, 1eq) was dissolved in MeCN (2 mL) and a solution of $[NEt_3Me]Cl$ (6 mg, 0.04 mmol, 1eq) in MeCN (0.5 mL) was added dropwise. The formed suspension was stirred for 1 h at room temperaute and filtered through a syringe

filter. The solvent was removed under reduced pressure and the obtained colorless solid was identified as **7** (60 mg, 0.03 mmol, 85%).

Single crystals suitable for X-ray diffraction were obtained by slow diffusion of *n*-pentane into a CH_2Cl_2 solution of **7**.

^{1}H NMR (400 MHz, CD_2Cl_2, 22 °C): δ = 3.27 (quart, 6H, $^3J(^1H,^1H)$ = 7.5 Hz, CH_2), 2.92 (s, 3H, NCH_3), 1.34 (t, 9H, CH_3) ppm.

^{19}F NMR (377 MHz, CD_2Cl_2, 22 °C): δ = 10.4 (tquint, 3F, $^4J(^{19}F,^{19}F_o)$ = 19 Hz, $^2J(^{19}F,^{19}F)$ = 81 Hz, $^1J(^{125}Te,^{19}F)$ = 2850 Hz), −23.5 (dquint, 6F, $^4J(^{19}F,^{19}F_o)$ = 19 Hz, $^1J(^{125}Te,^{19}F)$ = 2810 Hz), −130.5 (m, 12F, $^3J(^{19}F_o,^{19}F_m)$ = 19 Hz, *o*-F), −149.4 (m, 6F, $^4J(^{19}F_o,^{19}F_p)$ = 6 Hz, *p*-F), −161.8 (m, 12F, $^3J(^{19}F_p,^{19}F_m)$ = 19 Hz, *m*-F), −178.8 (s, 1F, Al–F) ppm.

^{27}Al NMR (128 MHz, CD_2Cl_2, 22 °C): δ = 48.1 (br s) ppm.

MS (ESI+): *m/z*: 116.1 $[NEt_3Me]^+$.

MS (ESI−): *m/z*: 1650.6 $[FAl(OTe^R)_3]^-$, 1070.7 $[H(OTe^R)_2]^-$, 536.9 $[OTe^R]^-$.

Synthesis of $[NEt_4][(F_5TeO)Al(OTe^R)_3]$ (**8**)

Method 1:

THF (90 µL, 1.10 mmol, 1 eq) was added to a solution of $AlEt_3$ (122 mg, 1.07 mmol, 1 eq) in toluene (5 mL) to prepare a stock solution. A portion of this solution (0.50 mL) was added dropwise to a suspension of $HOTe^R$ (170 mg, 0.32 mmol, 3 eq) in toluene (5 mL) at –45 °C. The reaction mixture was allowed to reach room temperature overnight. After cooling to –40 °C, a solution of $[NEt_4][OTeF_5]$ (39.1 mg, 0.11 mmol, 1 eq) in DCM (1 mL) was added. All volatiles were removed *in vacuo* to obtain a colorless solid which was washed with *n*-pentane (3 × 3 mL) and identified as **8** (200 mg, 0.10 mmol, 95%).

Method 2:

$HOTe^R$ (113 mg, 0.21 mmol, 3 eq) and $[NEt_4][OTeF_5]$ (26.0 mg, 0.07 mmol, 1 eq) were dissolved in DCM (5 mL) and cooled to –70 °C. A solution of $AlEt_3$ in DCM (0.5 mL, 80 mg $AlEt_3$ in 5 mL DCM, 1 eq) was cooled to the same temperature and added dropwise. The reaction mixture was allowed to reach room temperature overnight. All

volatiles were removed under reduced pressure and the residue washed with *n*-pentane (3 × 3 mL) to obtain compound **8** as a white solid (130 mg, 0.06 mmol, 93%).
Single crystals suitable for X-ray diffraction were grown from slow diffusion of *n*-pentane into a solution of compound **8** in DCM.

^{1}H NMR (400 MHz, CD_2Cl_2, 22 °C): δ = 3.17 (quart, 8H, $^{3}J(^{1}H,^{1}H)$ = 7.3 Hz, CH_2), 1.33 (t, 12H, CH_3) ppm.
^{19}F NMR (377 MHz, CD_2Cl_2, 22 °C): δ = 8.91 (tquint, 3F, $^{4}J(^{19}F,^{19}F_o)$ = 18 Hz, $^{2}J(^{19}F,^{19}F)$ = 77 Hz, $^{1}J(^{125}Te,^{19}F)$ = 2780 Hz), −22.7 (dquint, 6F, $^{4}J(^{19}F,^{19}F_o)$ = 21 Hz, $^{1}J(^{125}Te,^{19}F)$ = 2810 Hz), −38.0 (m, 1F, $^{2}J(^{19}F,^{19}F)$ = 186 Hz, $^{1}J(^{125}Te,^{19}F)$ = 3280 Hz, Te-F_{ax}), −48.5 (m, 4F, $^{1}J(^{125}Te,^{19}F)$ = 3476 Hz, Te-F_{eq}), −130.5 (m, 12F, $^{3}J(^{19}F_o,^{19}F_m)$ = 20 Hz, *o*-F), −149.4 (m, 6F, $^{4}J(^{19}F_o,^{19}F_p)$ = 6 Hz, *p*-F), −161.9 (m, 12F, $^{3}J(^{19}F_p,^{19}F_m)$ = 19 Hz, *m*-F) ppm.

^{27}Al NMR (128 MHz, CD_2Cl_2, 22 °C): δ = 43.4 (br s) ppm.

IR (ATR, 25°C): $\tilde{\nu}$ = 1638 (m), 1518 (s), 1488 (s, C_6F_5-Ring), 1391 (m), 1380 (m), 1290 (m), 1090 (s, C–F), 979 (s, C–F), 921 (m), 889 (Al–O), 811 (m, C_6F_5-Ring), 758 (m), 726 (m), 638 (s, Te–F), 599 (m), 504 (m) cm^{-1}.

Synthesis of Ag[(F_5TeO)Al(OTe^R)$_3$] (**9**)

THF (90 µL, 1.10 mmol, 1 eq) was added to a solution of $AlEt_3$ (122 mg, 1.07 mmol, 1 eq) in toluene (5 mL) to prepare a stock solution. A portion of this solution (0.50 mL) was added dropwise to a suspension of $HOTe^R$ (170 mg, 0.32 mmol, 3 eq) in toluene (5 mL) at –45 °C. The reaction mixture was allowed to reach room temperature overnight and then, after cooling to –40 °C, a solution of $AgOTeF_5$ (36.5 mg, 0.11 mmol, 1 eq) in toluene (1 mL) was added. All volatiles were removed *in vacuo* to obtain a colorless solid which was washed with *n*-pentane (3 × 3 mL) and identified as **9** (195 mg, 0.01 mmol, 93 %).
Single crystals suitable for X-ray diffraction were grown by slow cooling of a solution of **9** in toluene to –40 °C.

^{13}C{^{19}F} NMR (100 MHz, CD_3CN, 22 °C): δ = 146.1 (s, *o*-C), 144.1 (s, *p*-C), 138.0 (s, *m*-C) ppm.

^{19}F NMR (377 MHz, CD_2Cl_2, 22 °C): δ = 10.7 (tquint, 3F, $^4J(^{19}F,^{19}F_o)$ = 18 Hz, $^2J(^{19}F,^{19}F)$ = 76 Hz, $^1J(^{125}Te,^{19}F)$ = 2850 Hz), −21.4 (dquint, 6F, $^4J(^{19}F,^{19}F_o)$ = 19 Hz, $^1J(^{125}Te,^{19}F)$ = 2810 Hz), −36.8 (m, 1F, $^2J(^{19}F,^{19}F)$ = 186 Hz, $^1J(^{125}Te,^{19}F)$ = 3250 Hz, Te-F_{ax}), −47.6 (m, 4F, $^1J(^{125}Te,^{19}F)$ = 3450 Hz, Te-F_{eq}), −130.2 (m, 12F, $^3J(^{19}F_o,^{19}F_m)$ = 18 Hz, *o*-F), −149.1 (m, 6F, $^4J(^{19}F_o,^{19}F_p)$ = 6 Hz, *p*-F), −161.4 (m, 12F, $^3J(^{19}F_p,^{19}F_m)$ = 19 Hz, *m*-F) ppm.

^{27}Al NMR (128 MHz, CD_2Cl_2, 22 °C): δ = 43.1 (br s) ppm.

^{125}Te NMR (126 MHz, CD_2Cl_2, 22 °C): δ = 707 (dtm, $^1J(^{19}F, ^{125}Te)$ = 2850 Hz, $^1J(^{19}F, ^{125}Te)$ = 2770 Hz, OTeR), 556 (m, $^1J(^{19}F, ^{125}Te)$ = 3250 Hz, $^1J(^{19}F, ^{125}Te)$ = 3450 Hz, O*Te*F_5) ppm.

MS (ESI+): *m/z*: 106.9 Ag^+.

MS (ESI−): *m/z*: 1650.6 $[FAl(OTe^R)_3]^-$, 1070.7 $[H(OTe^R)_2]^-$, 536.9 $[OTe^R]^-$, 240.9 $[OTeF_5]^-$.

IR (ATR, 25°C): $\tilde{\nu}$ = 1640 (m), 1518 (s), 1488 (s, C_6F_5-Ring), 1393 (m), 1380 (s), 1290 (m), 1090 (s, C–F), 981 (s, C–F), 895 (Al–O), 807 (m, C_6F_5-Ring), 756 (m), 706 (m), 638 (s, Te–F), 605 (m), 513 (m) cm^{-1}.

Elemental analysis calcd for **9**·2 C_7H_8 (%): C 27.8 H 0.75; found: C 27.8 H 0.68

Reactivity of $Ag[(F_5TeO)Al(OTe^R)_3]$ with HCl in toluene or mesitylene

$Ag[(F_5TeO)Al(OTe^R)_3]$ (150 mg, 0.07 mmol, 1 eq) was dissolved in toluene or mesitylene (3 mL), cooled to –40 °C and mixed with HCl (1atm). After stirring for 10 min, a colorless solid was directly precipitating and the suspension turned red. Et_2O (0.5 mL) was added and a direct decolorization was observed. The reaction mixture was filtered and all volatiles where removed. The residue was washed with *n*-pentane (3 × 2 mL) and analyzed by NMR spectroscopy.

^{1}H NMR (400 MHz, CD_2Cl_2, 22 °C): δ = 16.5 (s, 1H), 4.10 (quart, $^3J(^1H,^1H)$ = 7. Hz, CH_2 t), 1.45 (t, CH_3) ppm.

^{19}F NMR (377 MHz, CD_2Cl_2, 22 °C): δ = 5.25 (tquint, 3F, $^4J(^{19}F,^{19}F_o)$ = 19 Hz, $^2J(^{19}F,^{19}F)$ = 71 Hz, $^1J(^{125}Te,^{19}F)$ = 2880 Hz), −26.1 (dquint, 6F, $^4J(^{19}F,^{19}F_o)$ = 19 Hz, $^1J(^{125}Te,^{19}F)$ = 2870 Hz), −38.9 (m, 1F, $^2J(^{19}F,^{19}F)$ = 180 Hz, $^1J(^{125}Te,^{19}F)$ = 3230 Hz, Te-F_{ax}), −47.0 (m, 4F, $^1J(^{125}Te,^{19}F)$ = 3450 Hz, Te-F_{eq}), −130.1 (m, 12F, $^3J(^{19}F_o,^{19}F_m)$ = 18 Hz, *o*-F),

−149.4 (m, 6F, $^4J(^{19}F_o,^{19}F_p)$ = 6 Hz, *p*-F), −162.4 (m, 12F, $^3J(^{19}F_p,^{19}F_m)$ = 19 Hz, *m*-F) ppm.

^{27}Al NMR (128 MHz, CD_2Cl_2, 22 °C): δ = 45.0 (br s) ppm.

Protonation of mesityl oxide

Mesityl oxide (~8 mg) was placed in a Young NMR tube. 0.7 mL of a 0.15 M solution of Ag[(F_5TeO)Al(OTe^R)$_3$] in *o*-DFB was added in the tube. The solution was mixed with HCl (1atm) leading to a precipitation of colorless solid. $^{13}C\{^1H\}$ NMR spectra were measured from the reaction mixture and the Δδ difference between the C_α and C_β carbon atom of mesityl oxide was obtained to be 81.0 ppm.

Synthesis of [Ph_3C][(F_5TeO)Al(OTe^R)$_3$] (**10**)

Ag[(F_5TeO)Al(OTe^R)$_3$] (100 mg, 0.05 mmol, 1 eq) was dissolved in DCM (3 mL), and a solution of $ClCPh_3$ (14 mg, 0.05 mmol, 1eq) in DCM (1 mL) was added. After stirring for 10 min, a colorless solid was precipitating and the suspension turned deep yellow. The suspension was filtered and all volatiles removed under reduced pressure. The deep yellow solid was identified as **10** (95 mg, 0.04 mmol, 90 %). Single crystals suitable for X-ray diffraction were grown by slow cooling of a solution of **10** in toluene to −24 °C.

^{1}H NMR (400 MHz, CD_2Cl_2, 22 °C): δ = 8.29 (m, *p*-CH, 1H), 7.90 (m, $^3J(^1H,^1H)$ = 7 Hz, *m*-H, 2H), 7.69 (m, 2H, *o*-H), ppm.

$^{13}C\{^1H\}$ NMR (100 MHz, CD_3CN, 22 °C): δ = 210.5 (s, Ph_3C^+), 147.1 (s, *o*-C), 144.1 (s, *p*-C), 139.6 (s, *m*-C), 136.2 (s), 130.4 (s), 128.7 (s), 126.0 (s) ppm.

^{19}F NMR (377 MHz, CD_2Cl_2, 22 °C): δ = 9.0 (tquint, 3F, $^4J(^{19}F,^{19}F_o)$ = 19 Hz, $^2J(^{19}F,^{19}F)$ = 77 Hz, $^1J(^{125}Te,^{19}F)$ = 2887 Hz), −22.6 (dquint, 6F, $^4J(^{19}F,^{19}F_o)$ = 19 Hz, $^1J(^{125}Te,^{19}F)$ = 2810 Hz), −37.9 (m, 1F, $^2J(^{19}F,^{19}F)$ = 180 Hz, $^1J(^{125}Te,^{19}F)$ = 3220 Hz, Te-F_{ax}), −48.4 (m, 4F, $^1J(^{125}Te,^{19}F)$ = 3450 Hz, Te-F_{eq}), −130.3 (m, 12F, $^3J(^{19}F_o,^{19}F_m)$ = 18 Hz, *o*-F), −149.4 (m, 6F, $^4J(^{19}F_o,^{19}F_p)$ = 6 Hz, *p*-F), −161.8 (m, 12F, $^3J(^{19}F_p,^{19}F_m)$ = 19 Hz, *m*-F) ppm.

^{27}Al NMR (128 MHz, CD_2Cl_2, 22 °C): δ = 42.0 (br s) ppm.

MS (ESI+): *m/z*: 243,1 [Ph_3C]$^+$, 129.9 Te^+.

MS (ESI−): *m/z*: 1650.6 [FAl(OTe^R)$_3$]$^-$, 1070.7 [H(OTe^R)$_2$]$^-$, 240.9 [$OTeF_5$]$^-$.

Elemental analysis calcd (%): C 31.3, H 0.72; found: C 31.5 H 0.78.

2 NMR Spectra

Al(OTe^R)$_3$·THF (**1**·THF)

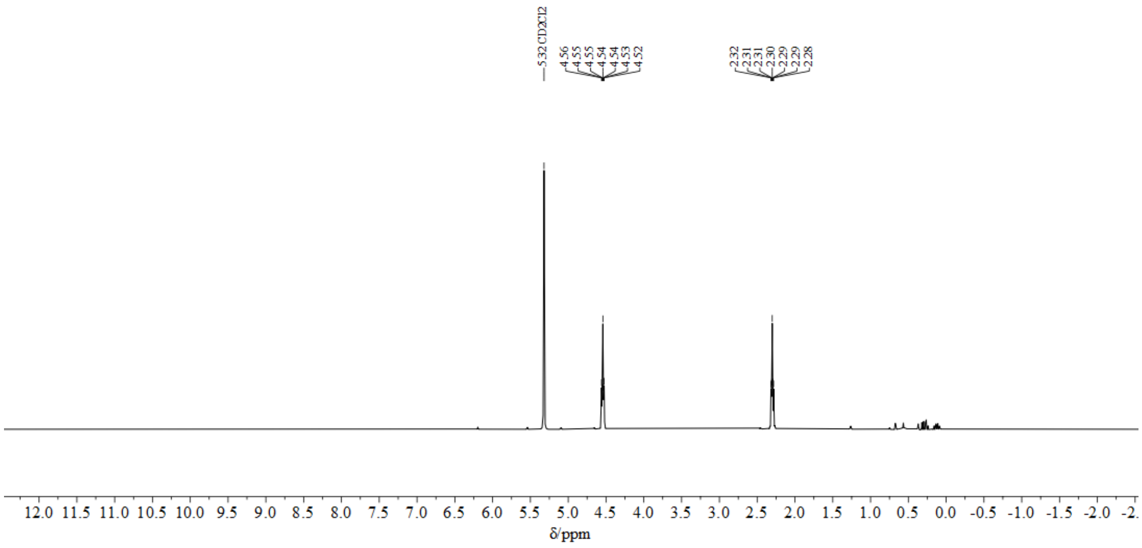

Figure S1. ^{1}H NMR spectrum (400 MHz, CD_2Cl_2, 22 °C) of Al(OTe^R)$_3$·THF (**1**·THF).

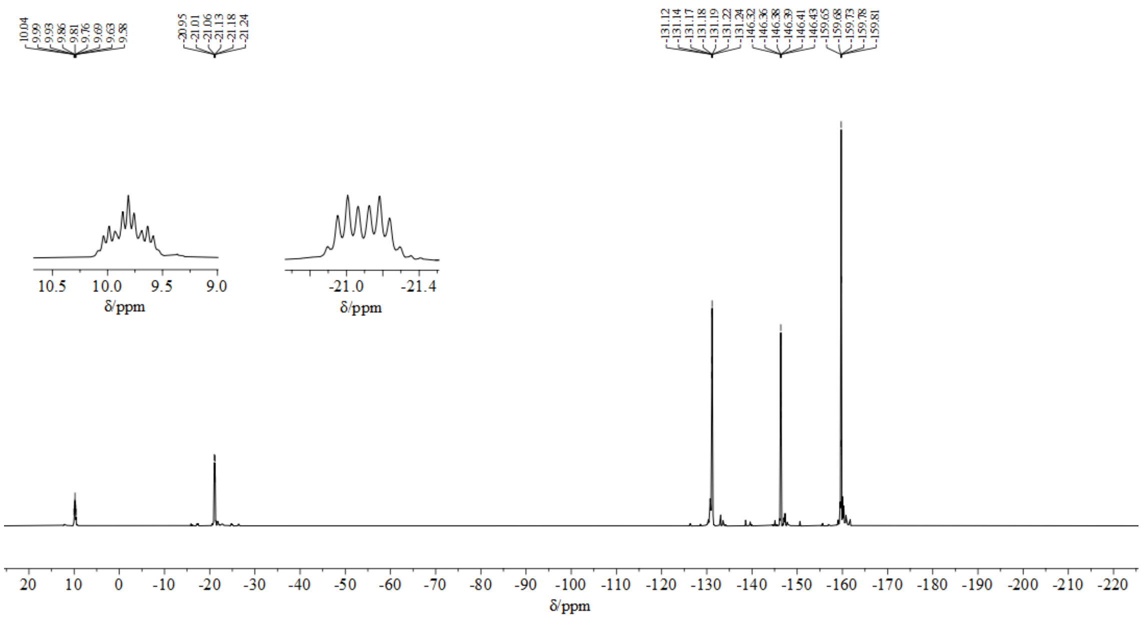

Figure S2. ^{19}F NMR spectrum (377 MHz, CD_2Cl_2, 22 °C) of Al(OTe^R)$_3$·THF (**1**·THF).

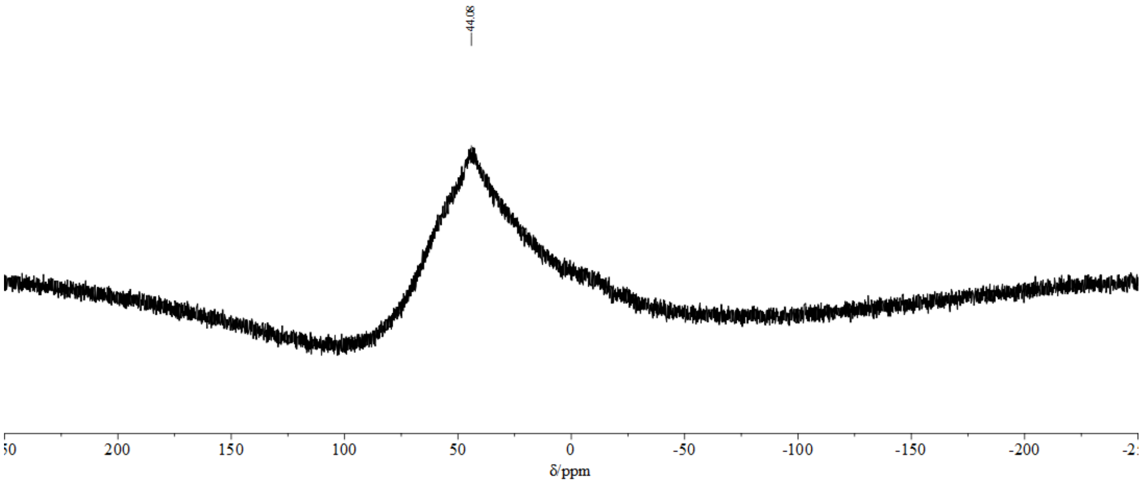

Figure S3. ^{27}Al NMR spectrum (128 MHz, CD_2Cl_2, 22 °C) of Al(OTe^R)$_3$·THF (**1**·THF).

Al(OTe^R)$_3$·DMC (**1**·DMC)

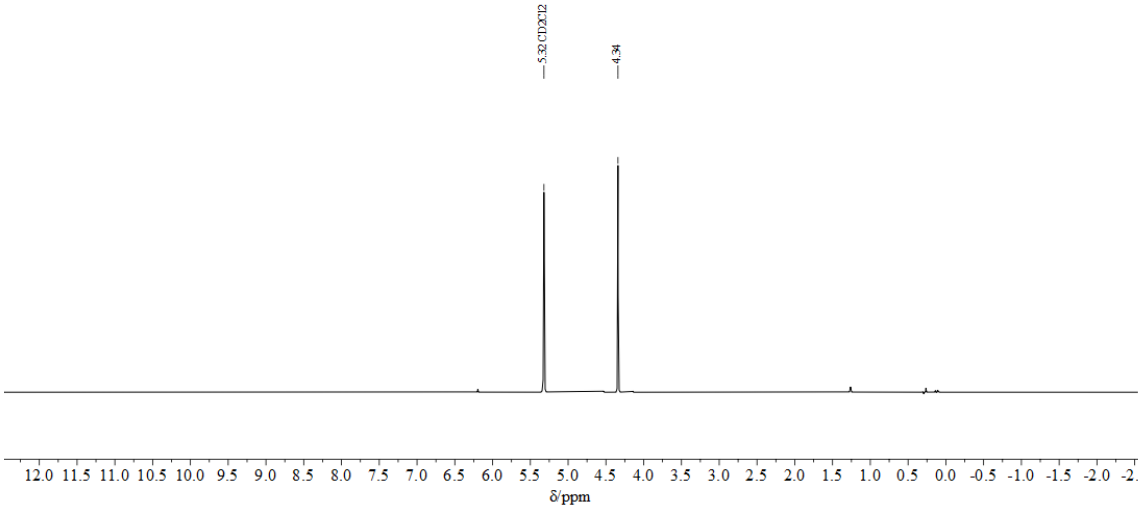

Figure S4. ^{1}H NMR spectrum (400 MHz, CD_2Cl_2, 22 °C) of Al(OTe^R)$_3$·DMC (**1**·DMC).

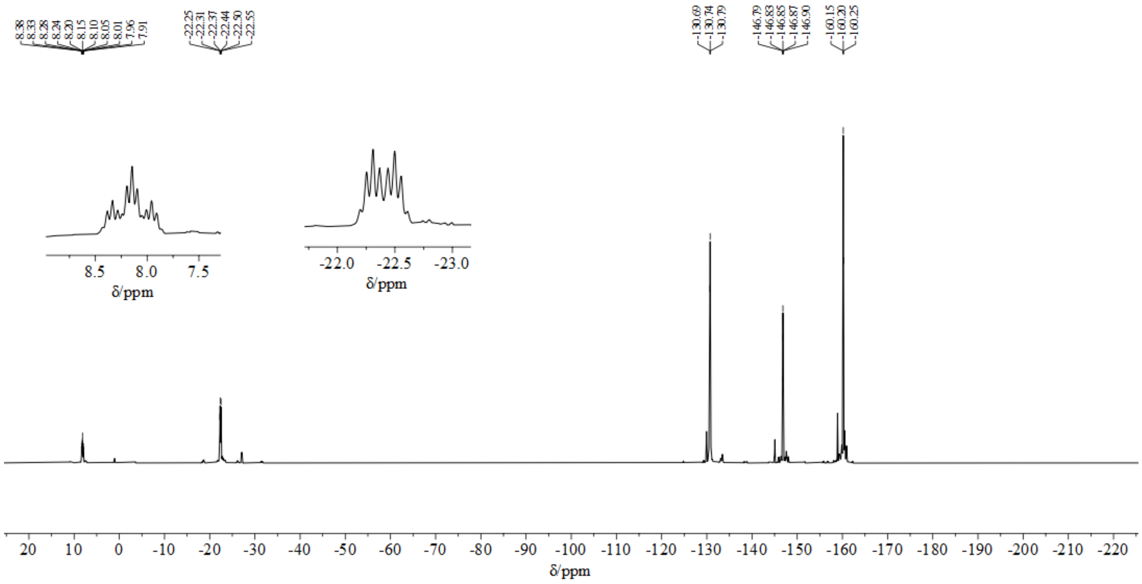

Figure S5. ^{19}F NMR spectrum (377 MHz, CD_2Cl_2, 22 °C) of $Al(OTe^R)_3$·DMC (**1**·DMC).

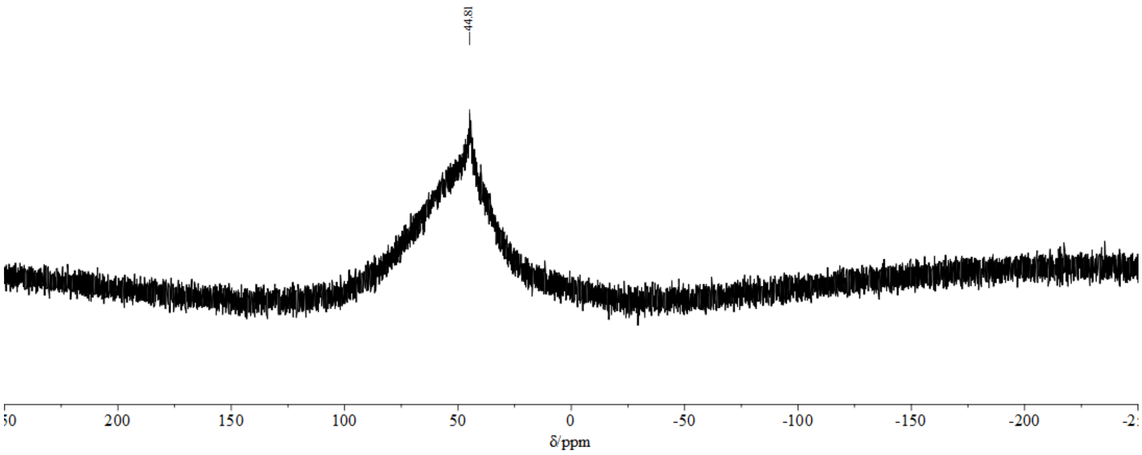

Figure S6. ^{27}Al NMR spectrum (128 MHz, CD_2Cl_2, 22 °C) of $Al(OTe^R)_3$·DMC (**1**·DMC).

$Al(OTe^R)_3 \cdot OPEt_3$ (**1**·$OPEt_3$)

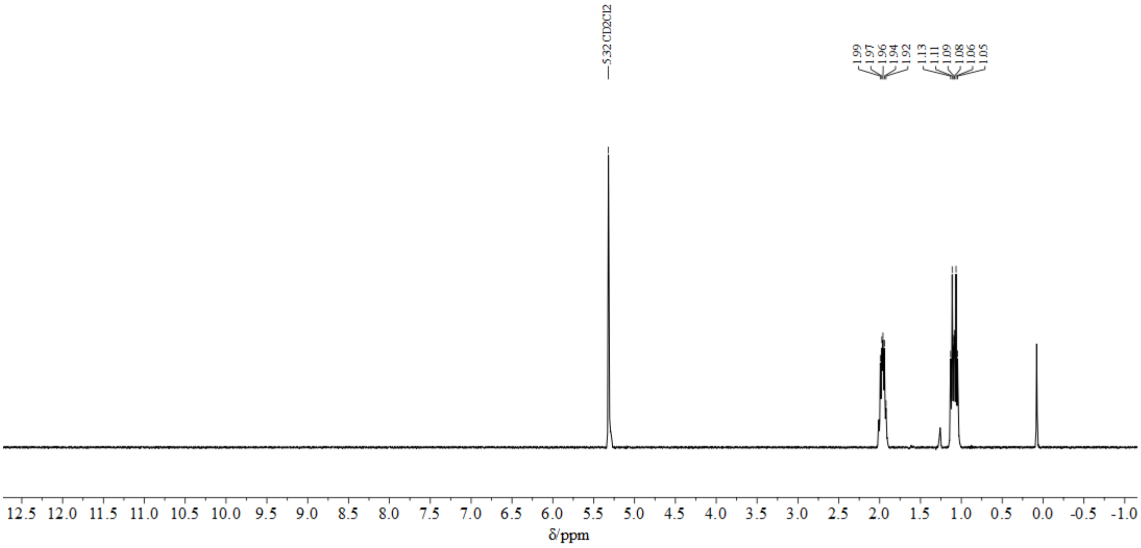

Figure S7. ^1H NMR spectrum (400 MHz, CD_2Cl_2, 22 °C) of $Al(OTe^R)_3 \cdot OPEt_3$ (**1**·$OPEt_3$).

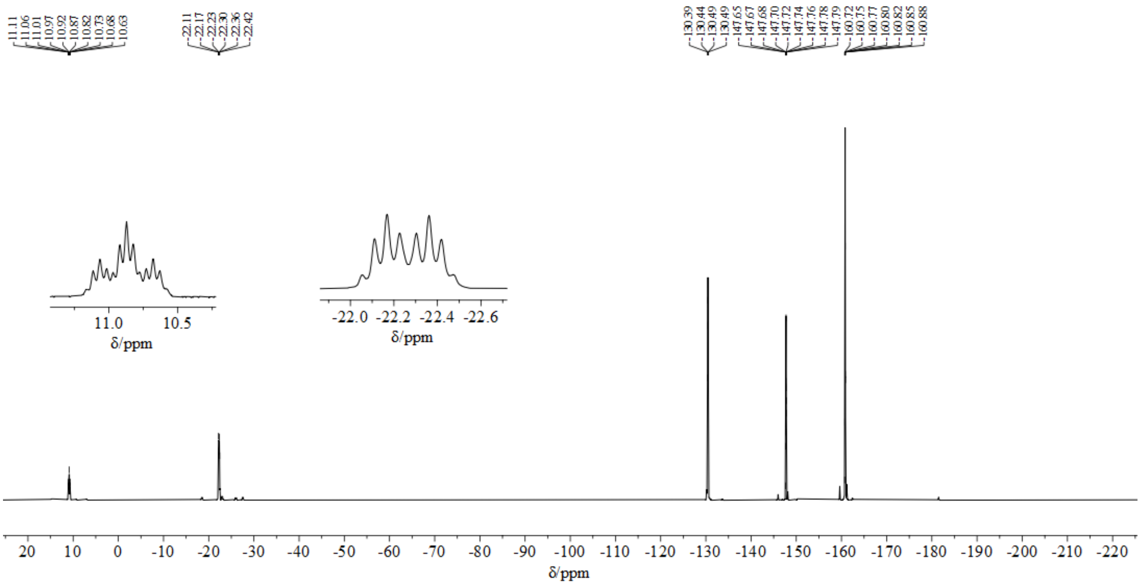

Figure S8. ^{19}F NMR spectrum (377 MHz, CD_2Cl_2, 22 °C) of $Al(OTe^R)_3 \cdot OPEt_3$ (**1**·$OPEt_3$).

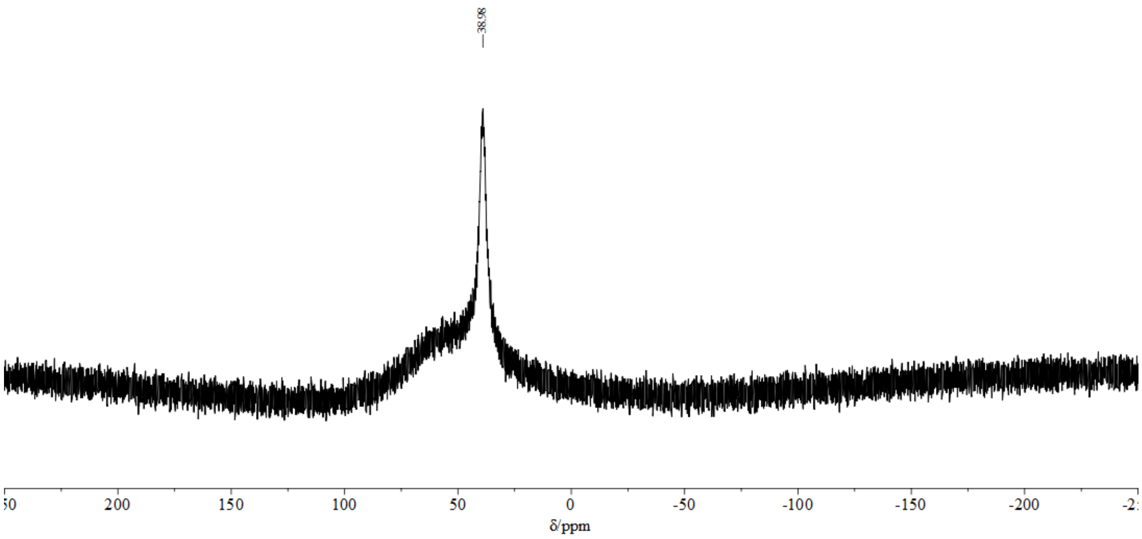

Figure S9. ^{27}Al NMR spectrum (128 MHz, CD_2Cl_2, 22 °C) of $Al(OTe^R)_3{\cdot}OPEt_3$ (**1**$\cdot OPEt_3$).

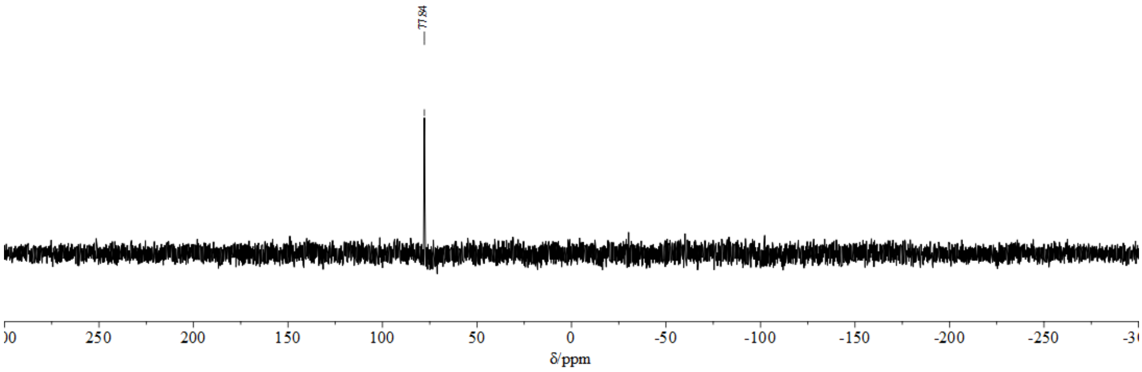

Figure S10. $^{31}P\{^1H\}$ NMR spectrum (104 MHz, CD_2Cl_2, 22 °C) of $Al(OTe^R)_3{\cdot}OPEt_3$ (**1**$\cdot OPEt_3$).

AlEt(OTe^R)$_2$·OPPh$_3$ (**3**)

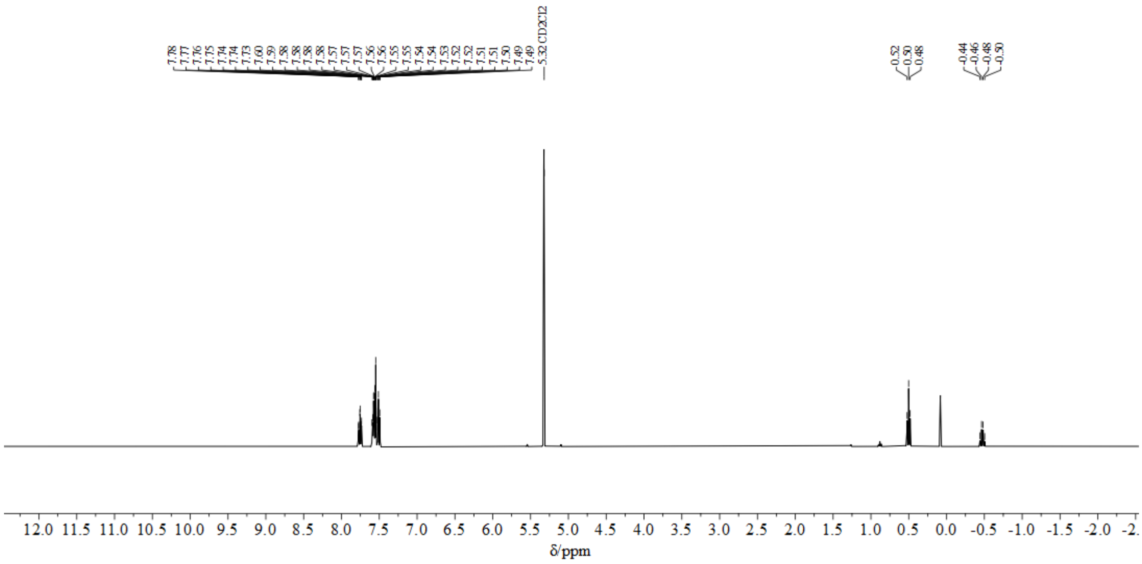

Figure S11. ^{1}H NMR spectrum (400 MHz, CD_2Cl_2, 22 °C) of AlEt(OTe^R)$_2$·OPPh$_3$ (**3**).

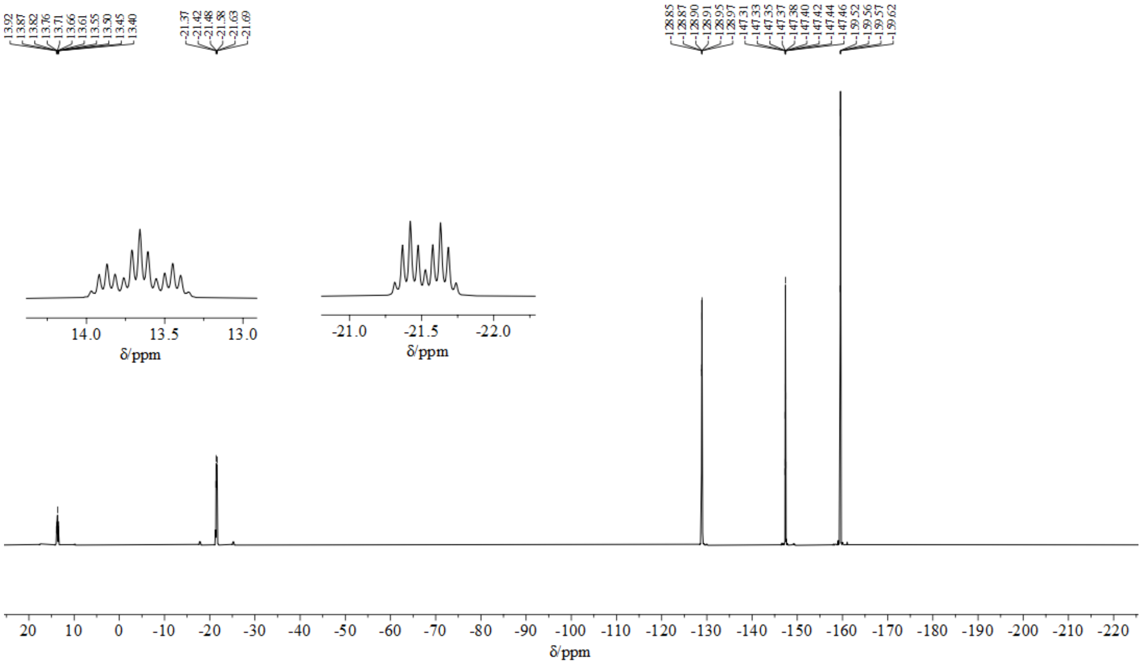

Figure S12. ^{19}F NMR spectrum (377 MHz, CD_2Cl_2, 22 °C) of AlEt(OTe^R)$_2$·OPPh$_3$ (**3**).

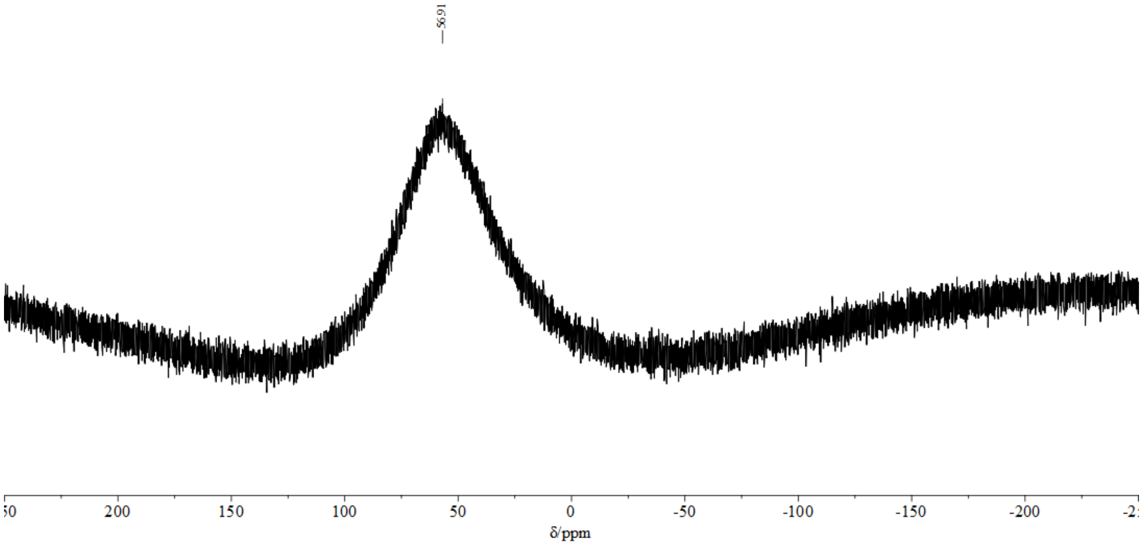

Figure S13. ^{27}Al NMR spectrum (128 MHz, CD_2Cl_2, 22 °C) of AlEt(OTe^R)$_2$·$OPPh_3$ (**3**).

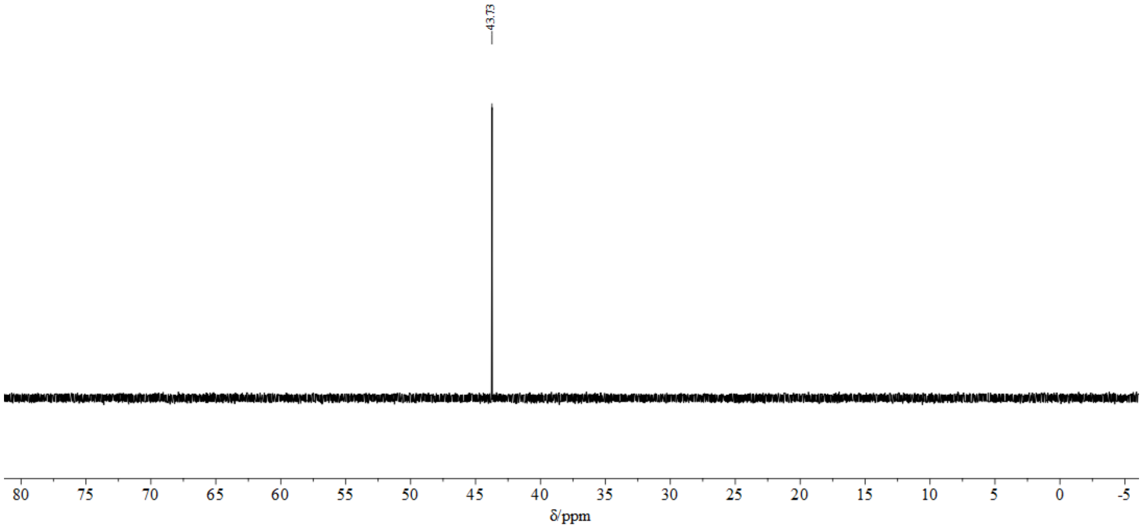

Figure S14. ^{31}P{^{1}H} NMR spectrum (104 MHz, CD_2Cl_2, 22 °C) of AlEt(OTe^R)$_2$·$OPPh_3$ (**3**).

$GaEt(OTe^{R})_2 \cdot OPEt_3$ (**4**)

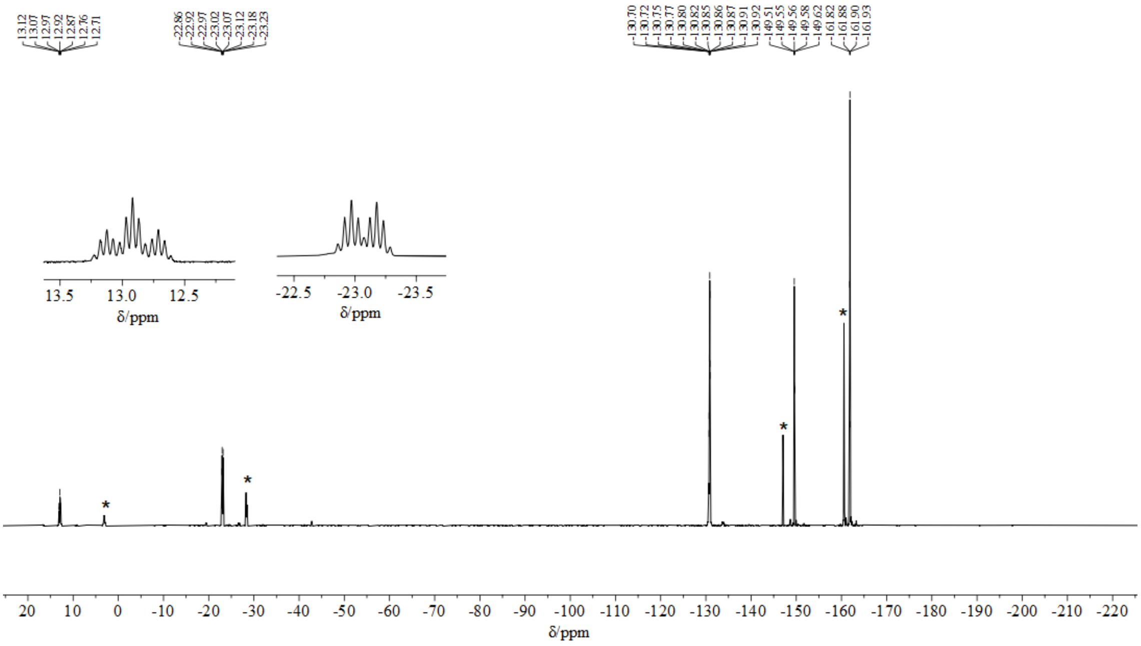

Figure S15. ^{19}F NMR spectrum (377 MHz, CD_2Cl_2, 22 °C) of $GaEt(OTe^{R})_2 \cdot OPPh_3$ (**4**).*=$HOTe^{R}$

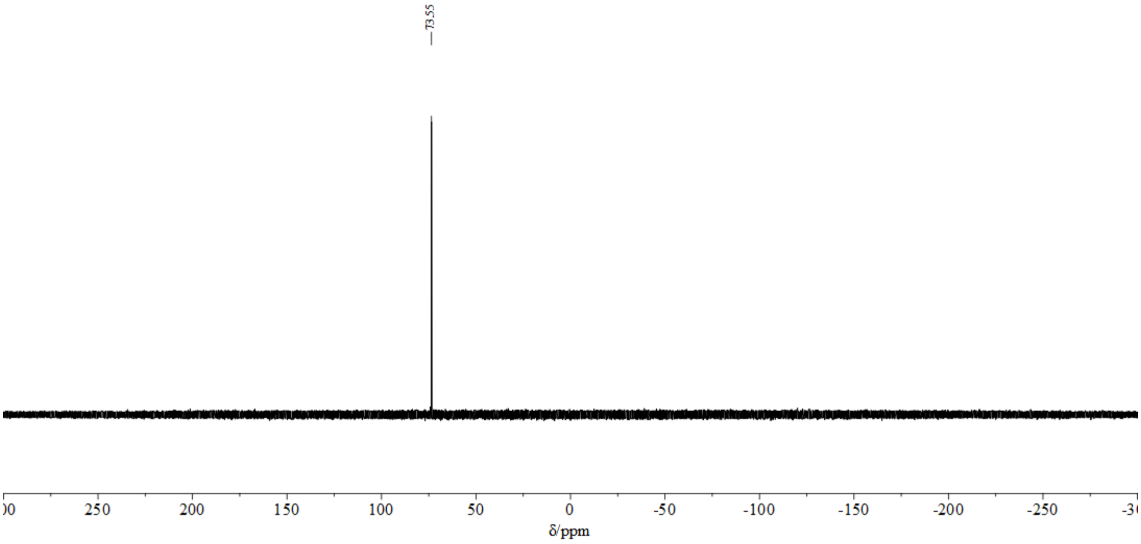

Figure S16. $^{31}P\{^1H\}$ NMR spectrum (104 MHz, CD_2Cl_2, 22 °C) of $GaEt(OTe^{R})_2 \cdot OPPh_3$ (**4**).

K[FAl(OTe^R)$_3$] (**5**)

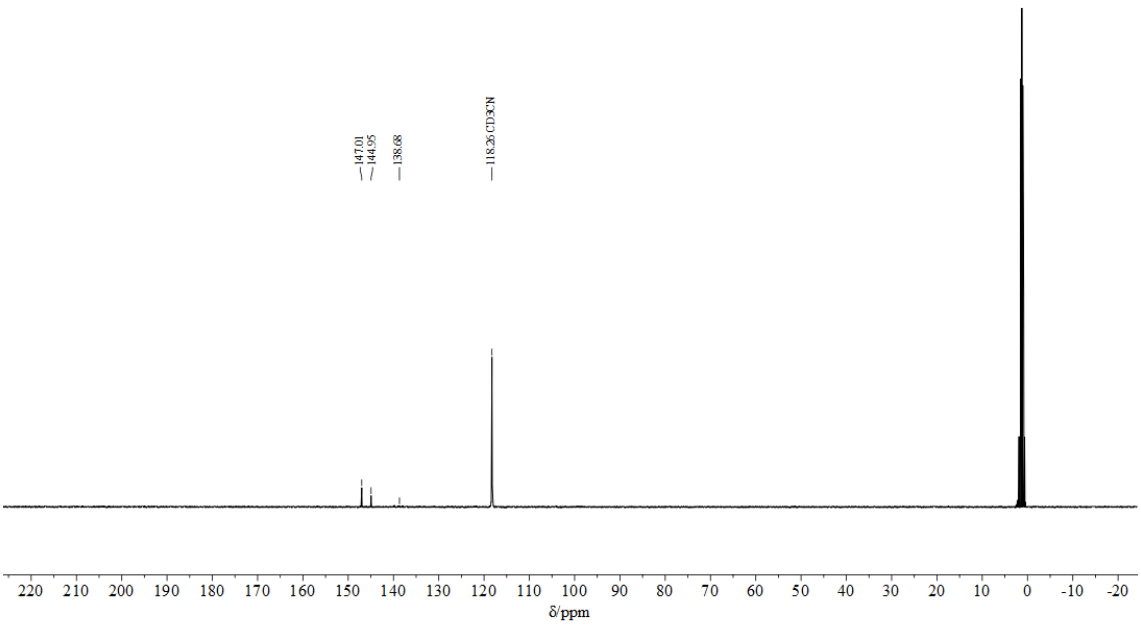

Figure S17. ^{13}C{^{19}F} NMR spectrum (100 MHz, CD_3CN, 22 °C) of K[FAl(OTe^R)$_3$] (**5**).

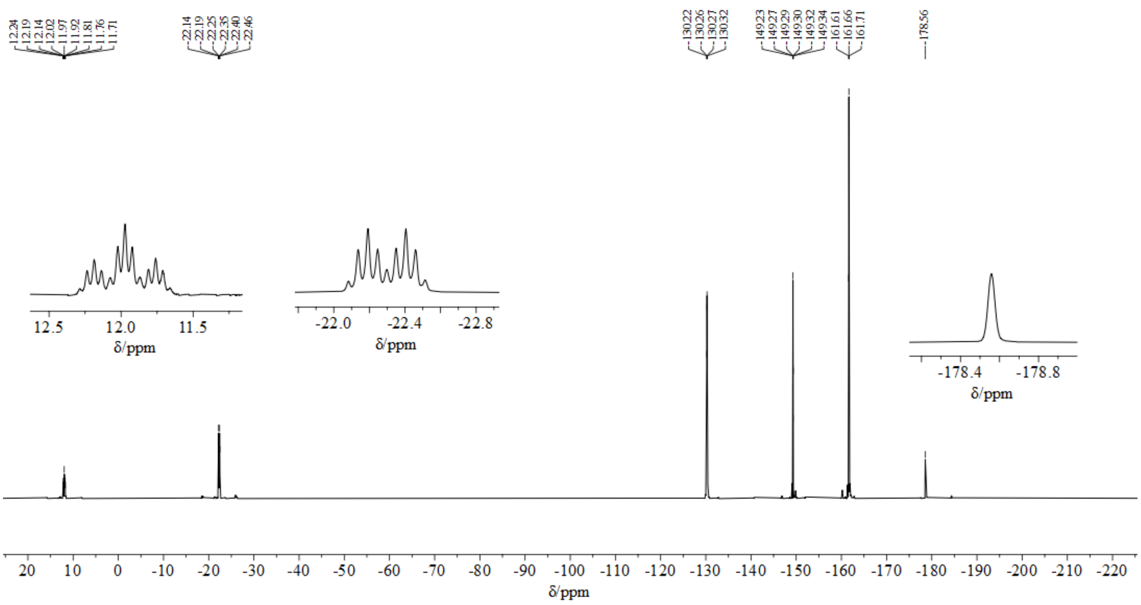

Figure S18. ^{19}F NMR spectrum (377 MHz, CD_3CN, 22 °C) of K[FAl(OTe^R)$_3$] (**5**).

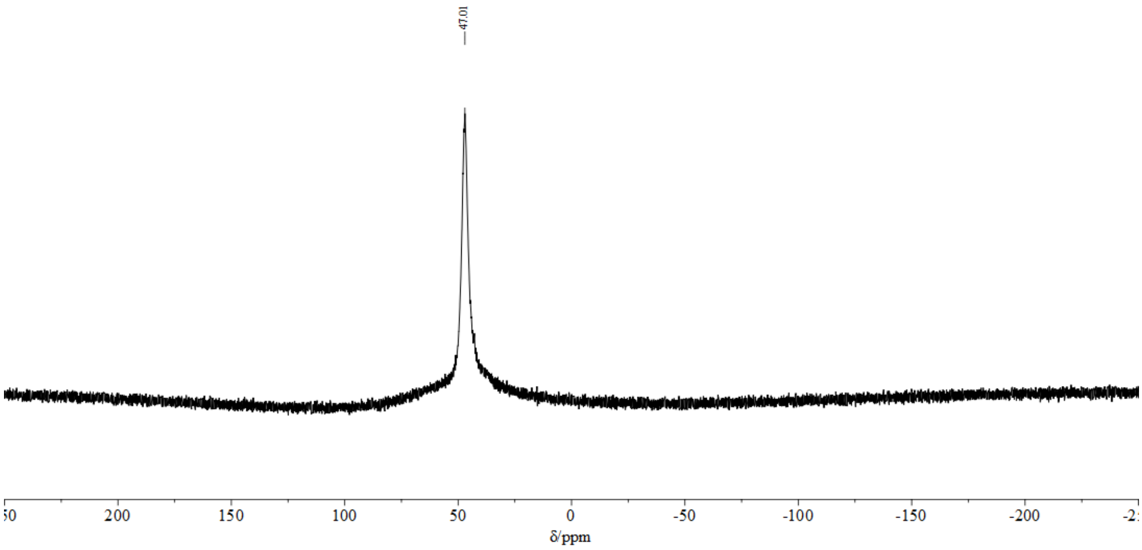

Figure S19. ^{27}Al NMR spectrum (128 MHz, CD_3CN, 22 °C) of K[FAl(OTe^R)$_3$] (**5**).

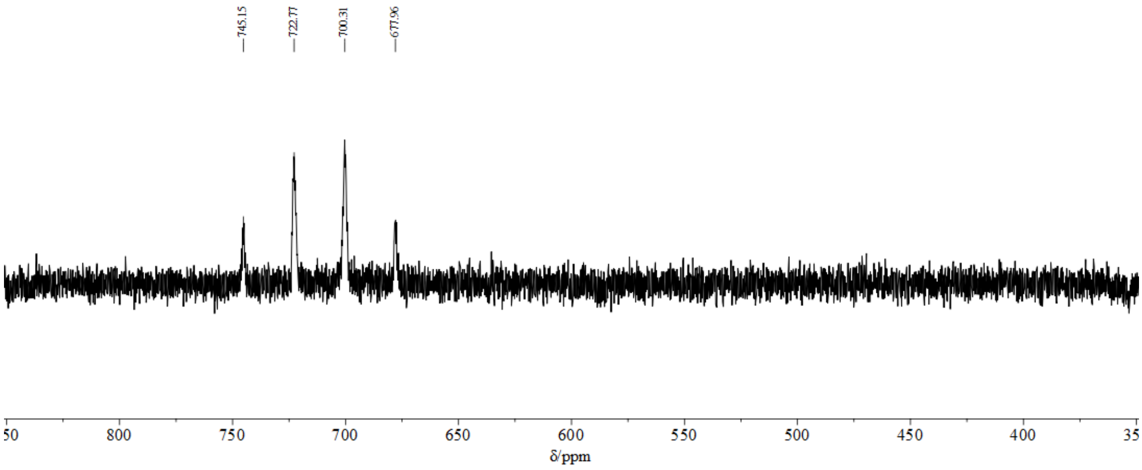

Figure S20. ^{125}Te NMR spectrum (126 MHz, CD_3CN, 22 °C) of K[FAl(OTe^R)$_3$] (**5**).

Cs[FAl(OTe^{R})$_3$] (**6**)

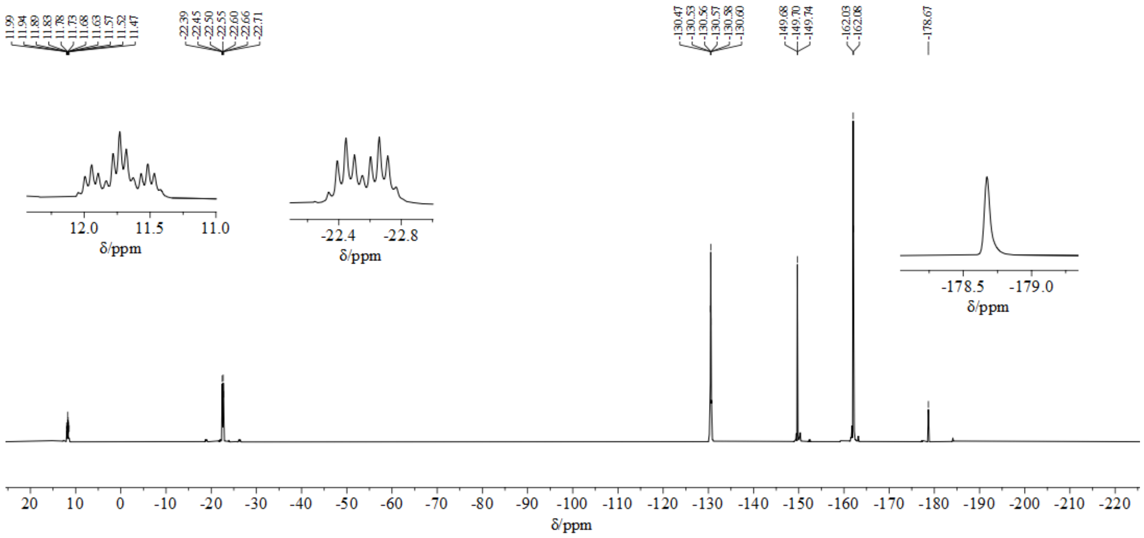

Figure S21. ^{19}F NMR spectrum (377 MHz, CD_3CN, 22 °C) of Cs[FAl(OTe^{R})$_3$] (**6**).

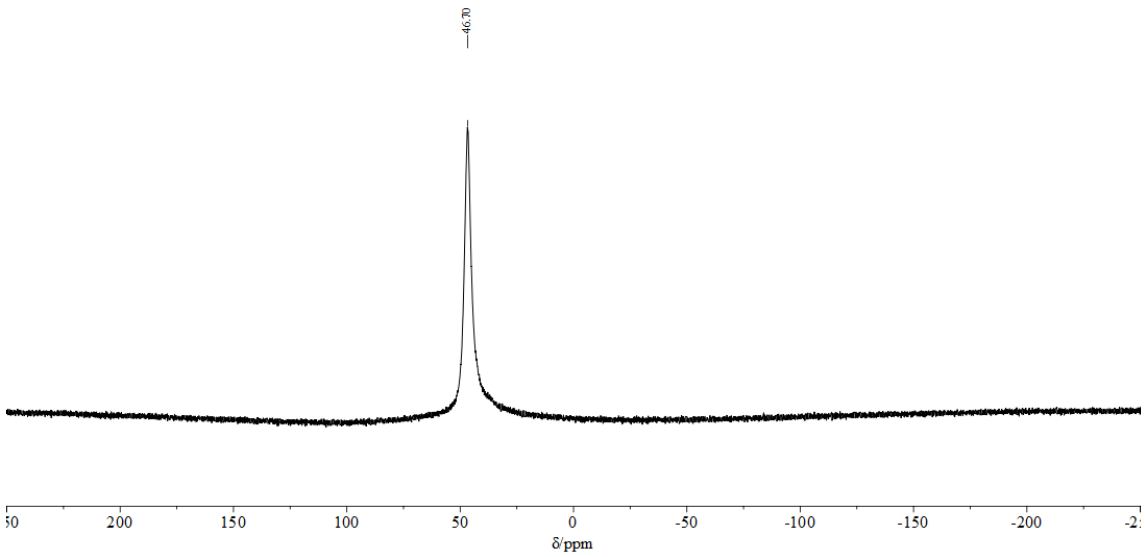

Figure S22. ^{27}Al NMR spectrum (128 MHz, CD_3CN, 22 °C) of Cs[FAl(OTe^{R})$_3$] (**6**).

[NEt_3Me][$FAl(OTe^R)_3$] (**7**)

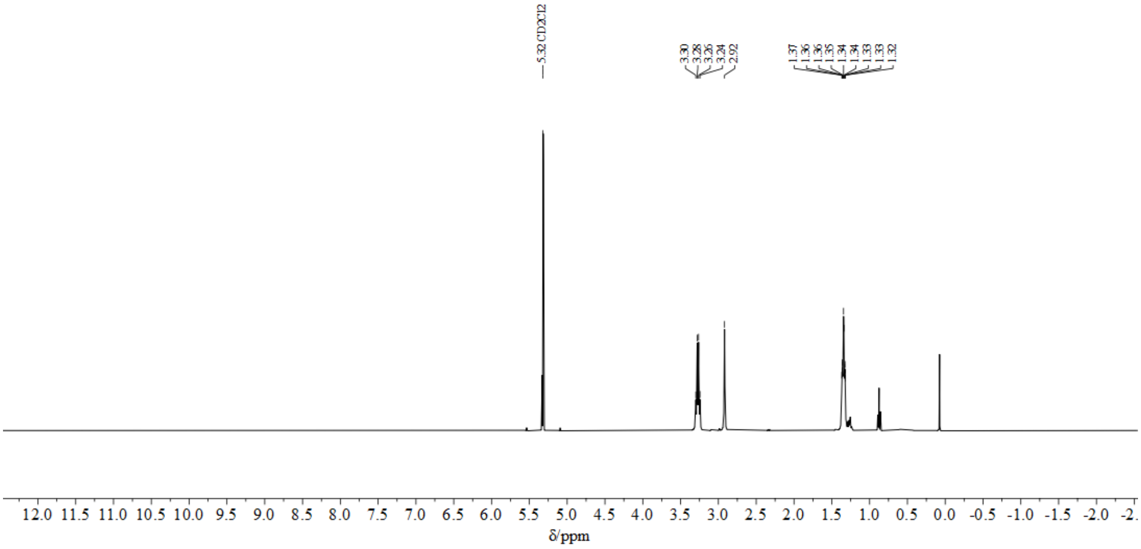

Figure S23. ^1H NMR spectrum (400 MHz, CD_2Cl_2, 22 °C) of [NEt_3Me][$FAl(OTe^R)_3$] (**7**).

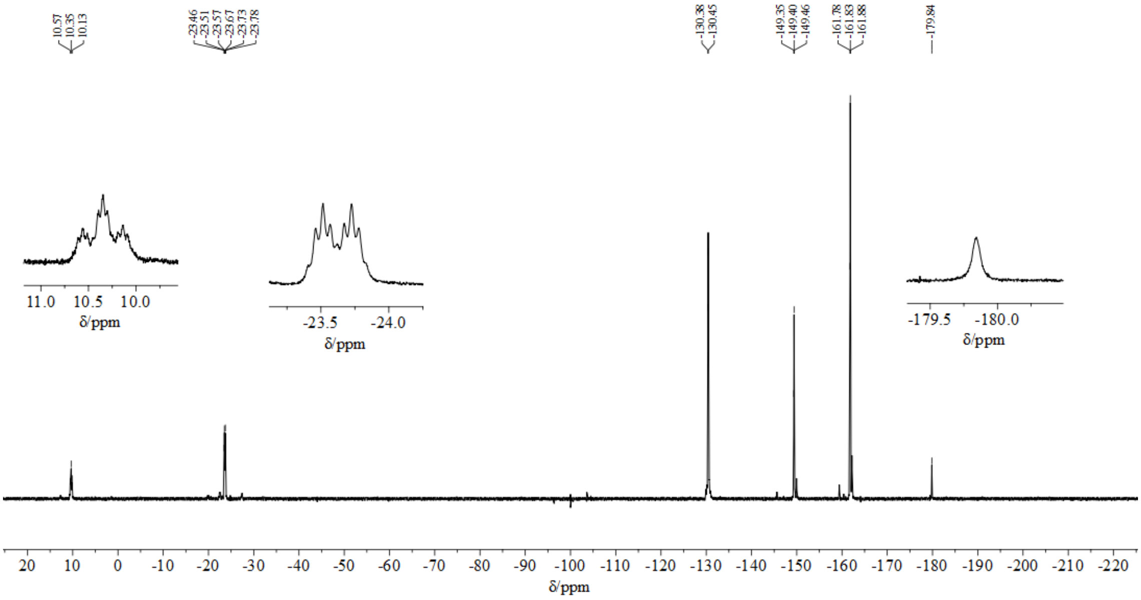

Figure S24. ^{19}F NMR spectrum (377 MHz, CD_2Cl_2, 22 °C) of [NEt_3Me][$FAl(OTe^R)_3$] (**7**).

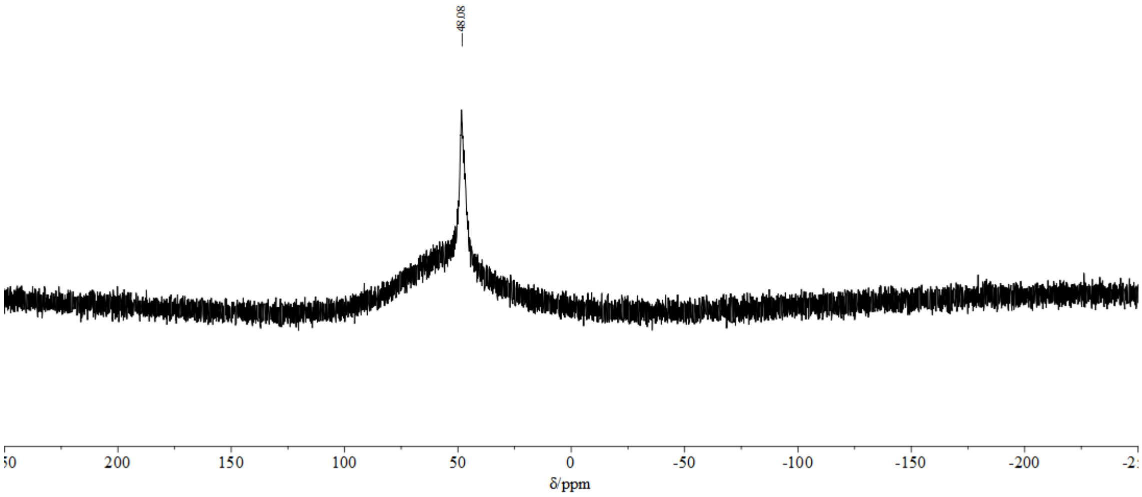

Figure S25. ^{27}Al NMR spectrum (128 MHz, CD_2Cl_2, 22 °C) of [NEt_3Me][FAl(OTe^R)$_3$] (**7**).

[NEt_4][(F_5TeO)Al(OTe^R)$_3$] (**8**)

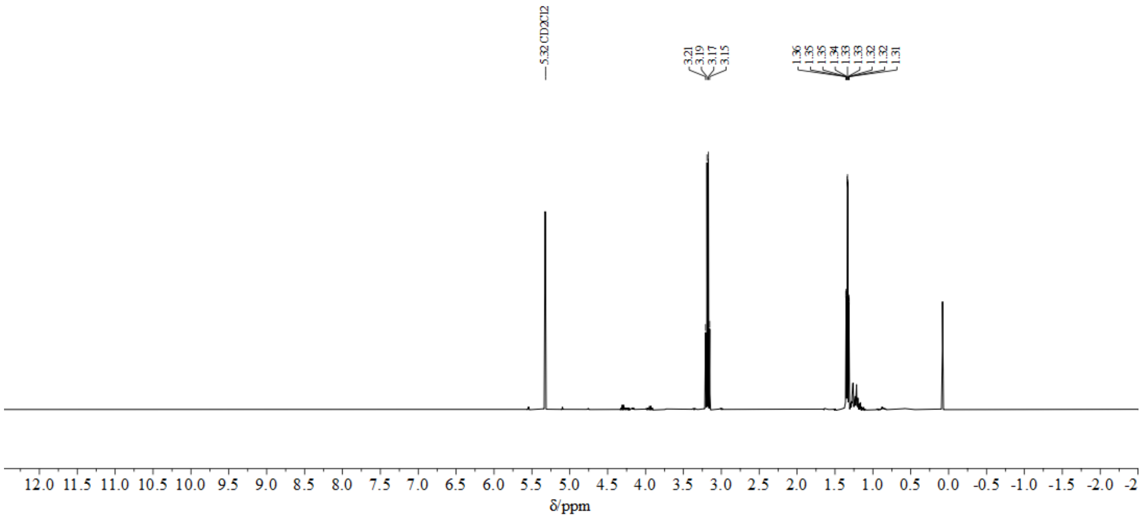

Figure S26. ^{1}H NMR spectrum (400 MHz, CD_2Cl_2, 22 °C) of [NEt_4][(F_5TeO)Al(OTe^R)$_3$] (**8**).

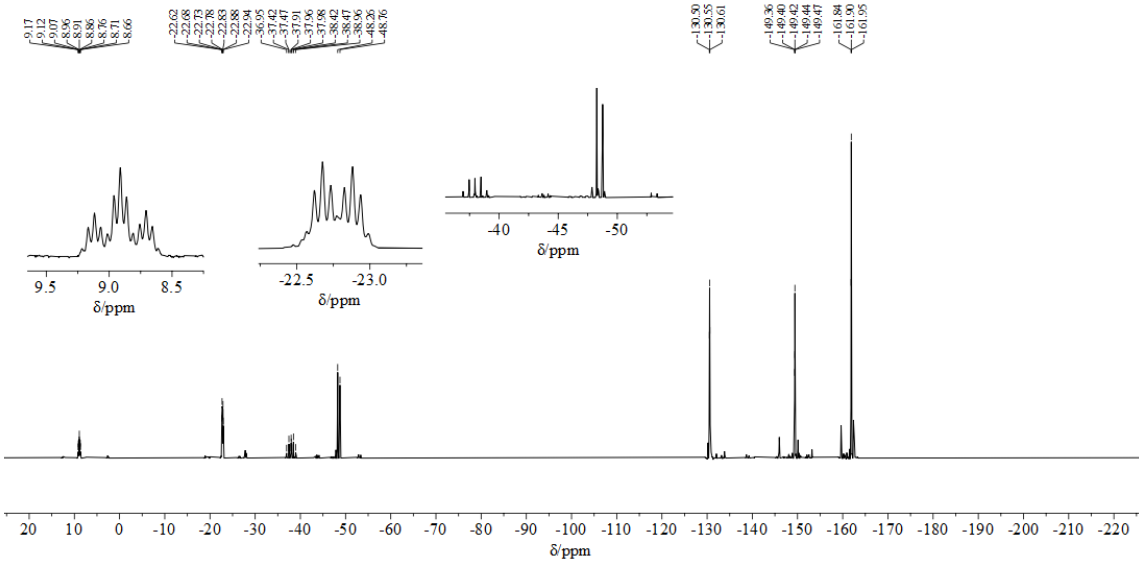

Figure S27. ^{19}F NMR spectrum (377 MHz, CD_2Cl_2, 22 °C) of $[NEt_4][(F_5TeO)Al(OTe^R)_3]$ (**8**).

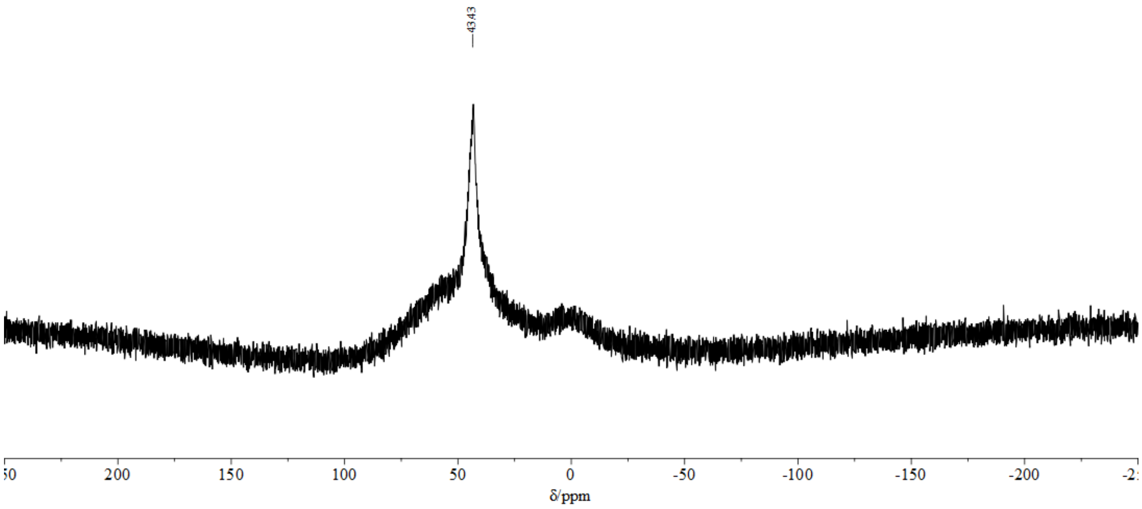

Figure S28. ^{27}Al NMR spectrum (128 MHz, CD_2Cl_2, 22 °C) of $[NEt_4][(F_5TeO)Al(OTe^R)_3]$ (**8**).

Ag[(F$_5$TeO)Al(OTe^R)$_3$] (**9**)

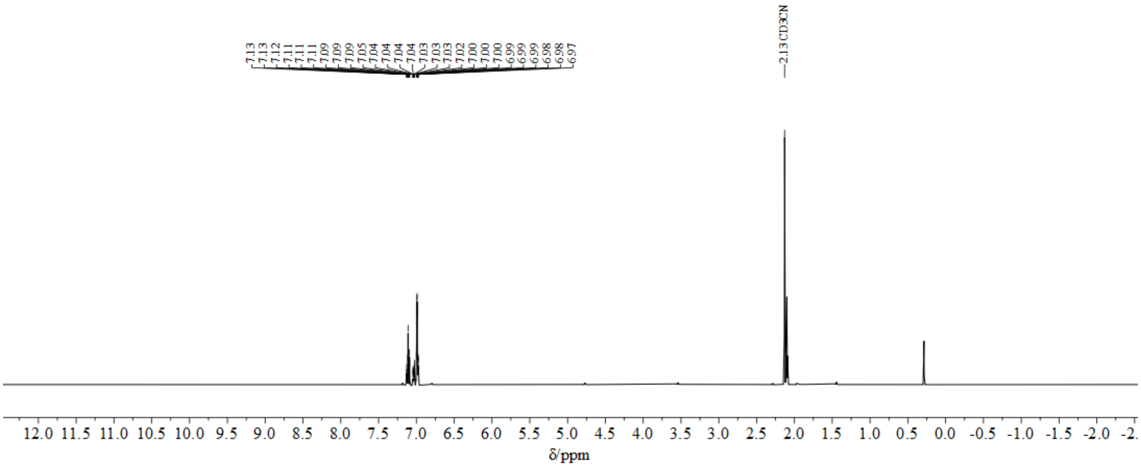

Figure S29. ^{1}H NMR spectrum (400 MHz, CD$_3$CN, 22 °C) of Ag[(F$_5$TeO)Al(OTe^R)$_3$] (**9**).

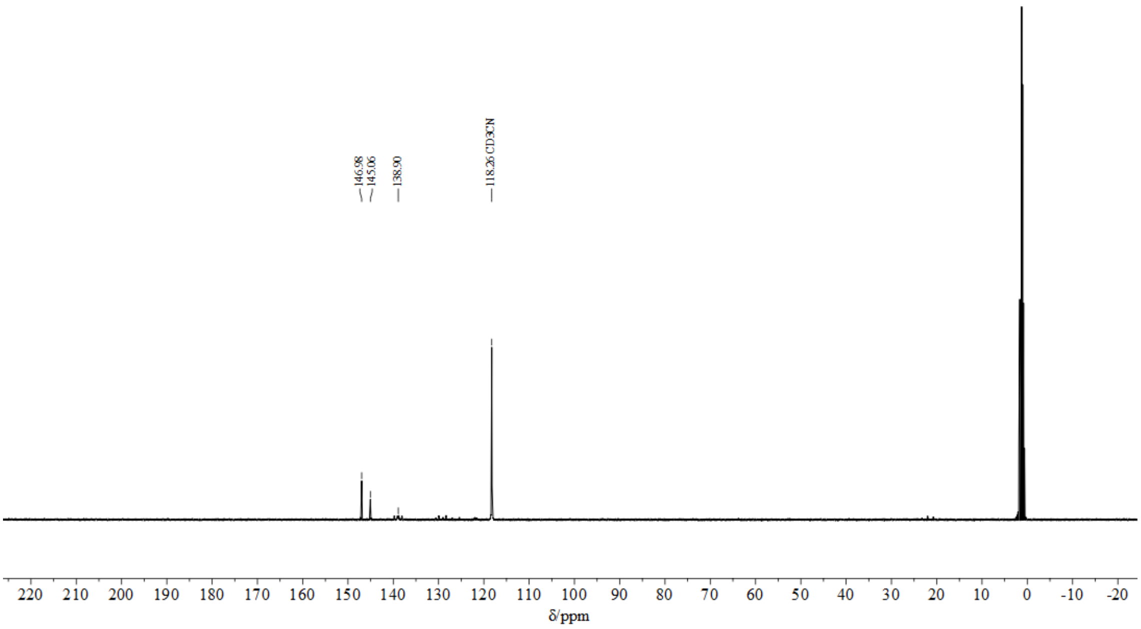

Figure S30. ^{13}C{^{19}F} NMR spectrum (100 MHz, CD$_3$CN, 22 °C) of Ag[(F$_5$TeO)Al(OTe^R)$_3$] (**9**).

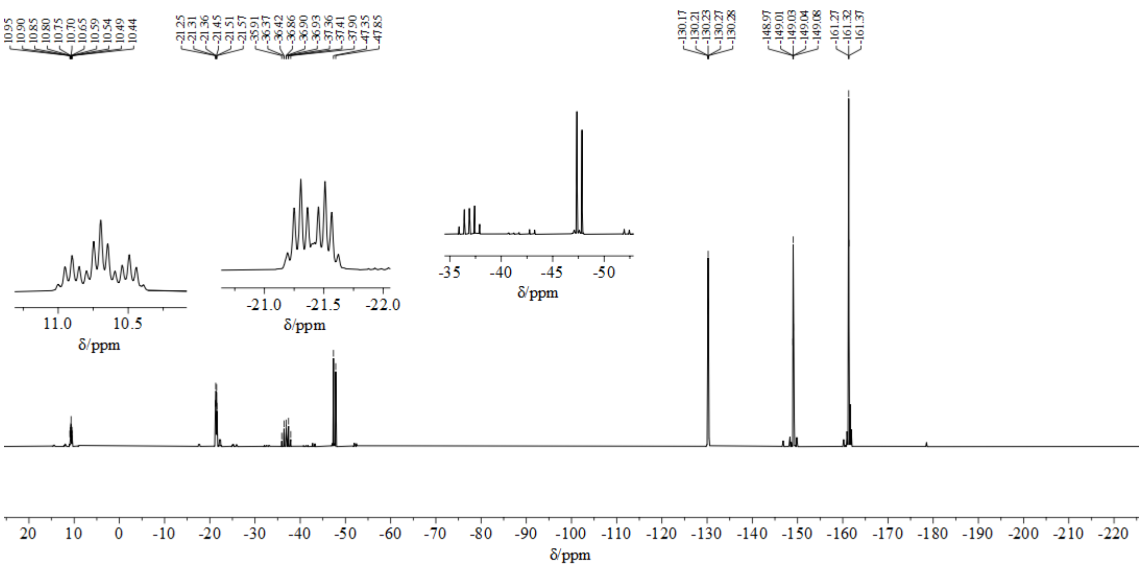

Figure S31. ^{19}F NMR spectrum (377 MHz, CD_3CN, 22 °C) of Ag[(F_5TeO)Al(OTe^R)$_3$] (**9**).

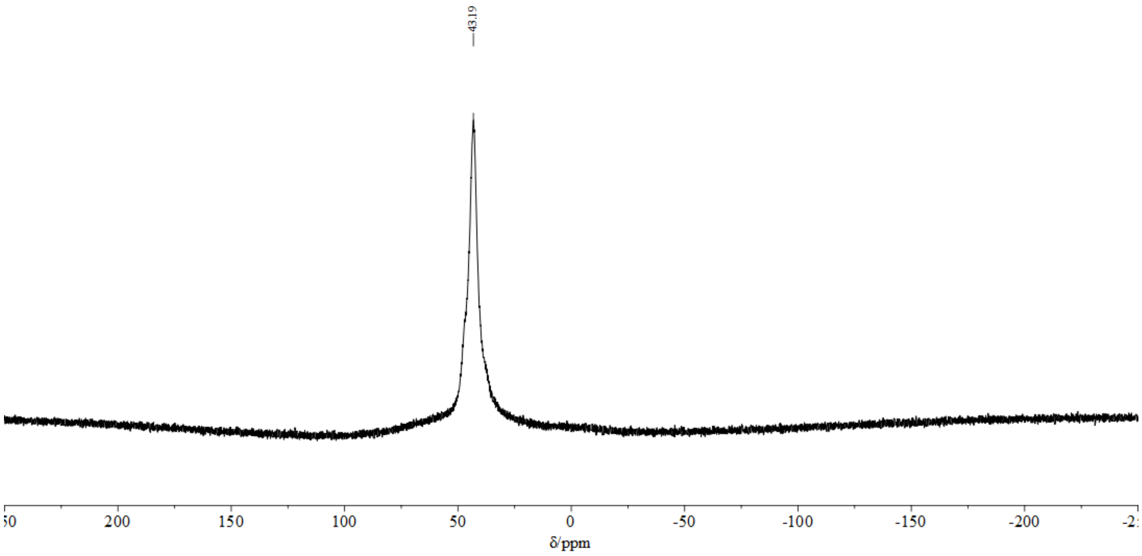

Figure S32. ^{27}Al NMR spectrum (128 MHz, CD_3CN, 22 °C) of Ag[(F_5TeO)Al(OTe^R)$_3$] (**9**).

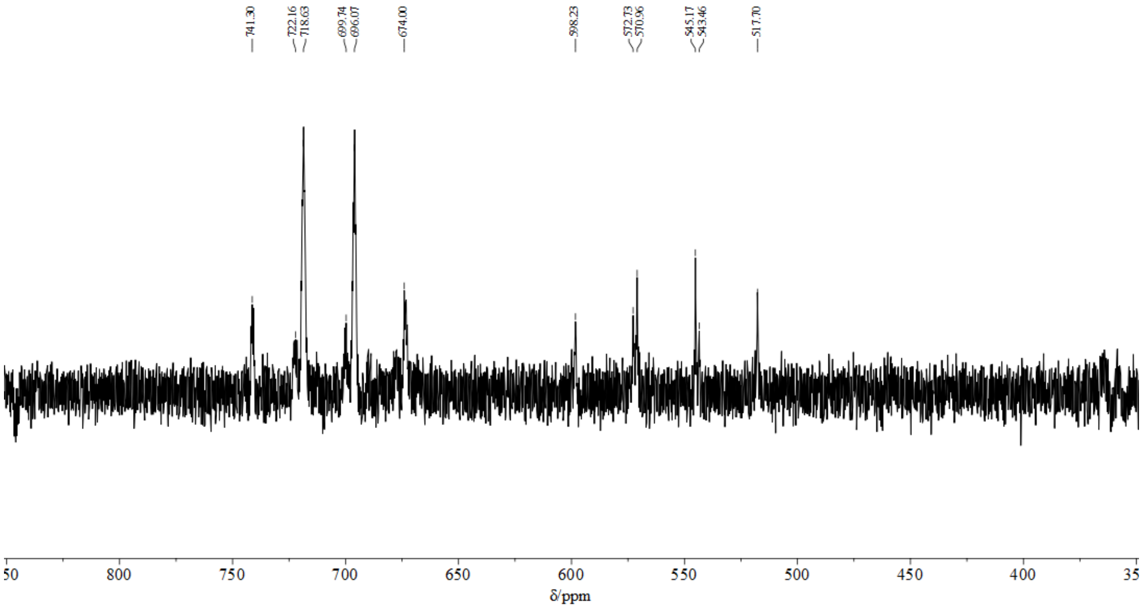

Figure S33. ^{125}Te NMR spectrum (126 MHz, CD_3CN, 22 °C) of Ag[(F_5TeO)Al(OTe^R)$_3$] (**9**).

Reactivity of Ag[(F_5TeO)Al(OTe^R)$_3$] with HCl in toluene or mesitylene

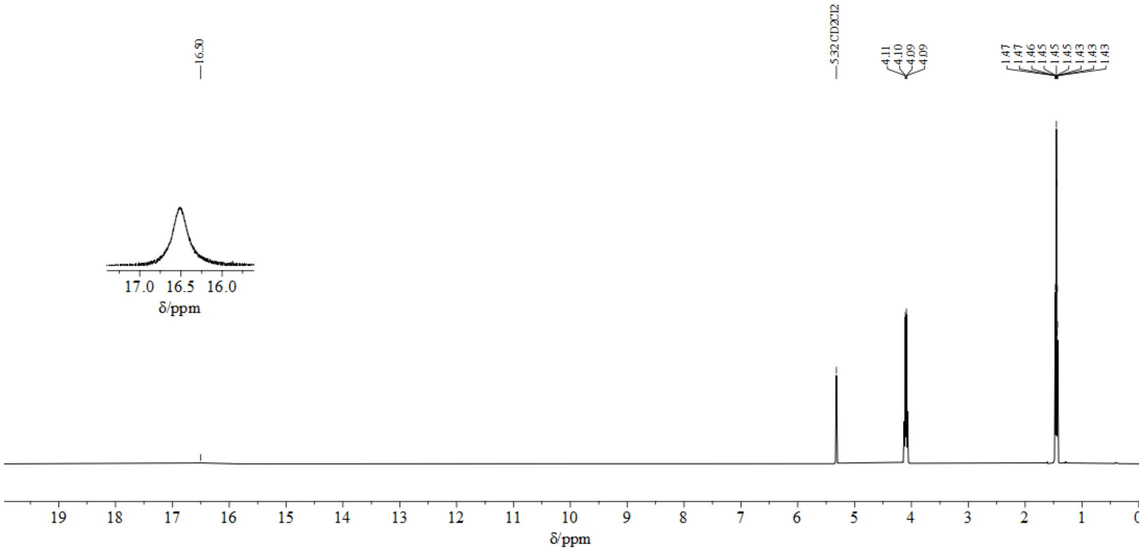

Figure S34. ^{1}H NMR spectrum (400 MHz, CD_2Cl_2, 22 °C) of [H(Et_2O)$_2$][(F_5TeO)Al(OTe^R)$_3$].

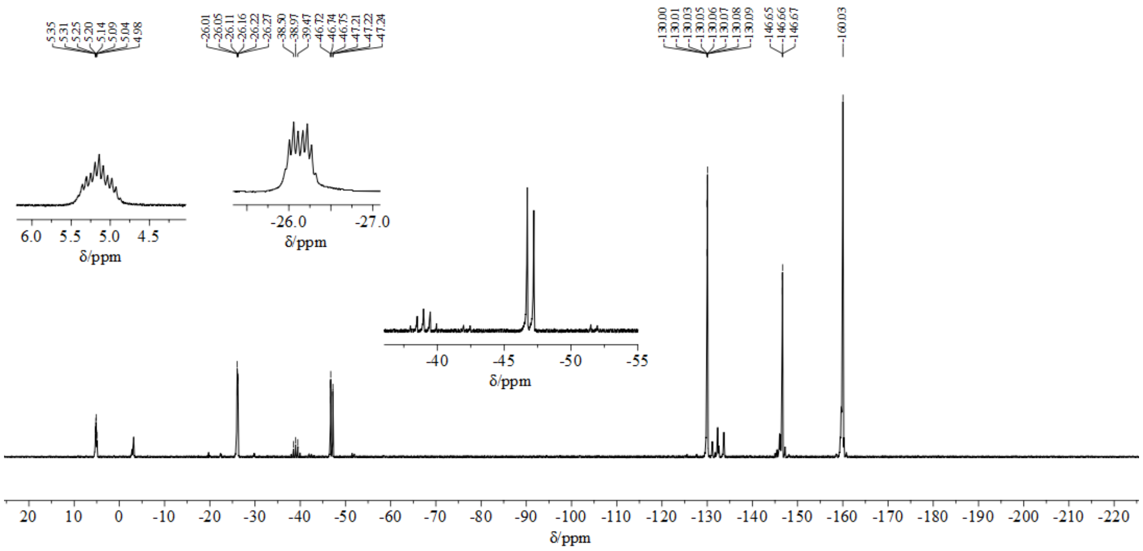

Figure S35. ^{19}F NMR spectrum (377 MHz, CD_2Cl_2, 22 °C) of $[H(Et_2O)_2][(F_5TeO)Al(OTe^R)_3]$.

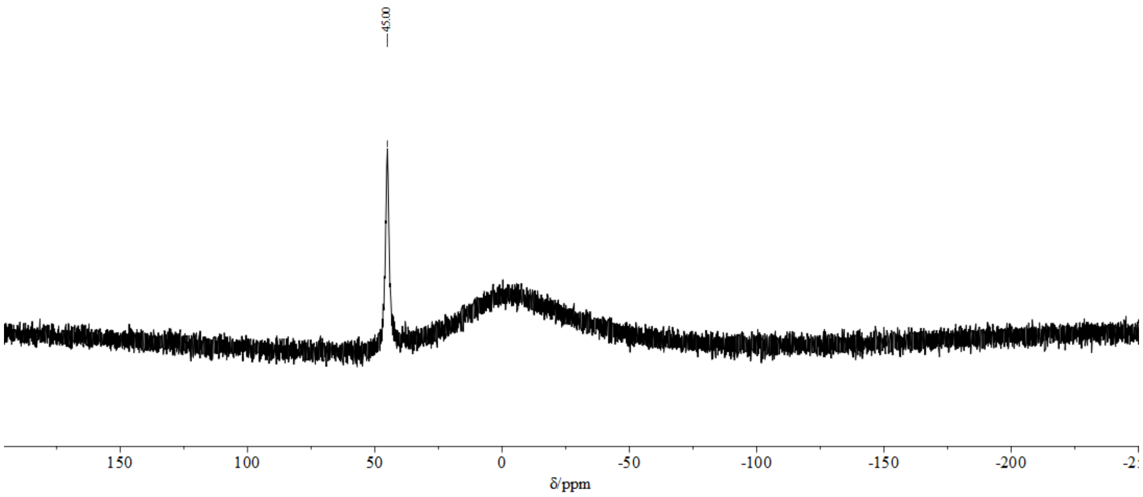

Figure S36. ^{27}Al NMR spectrum (128 MHz, CD_2Cl_2, 22 °C) of $[H(Et_2O)_2][(F_5TeO)Al(OTe^R)_3]$.

[Ph_3C][(F_5TeO)Al(OTe^R)$_3$] (**10**)

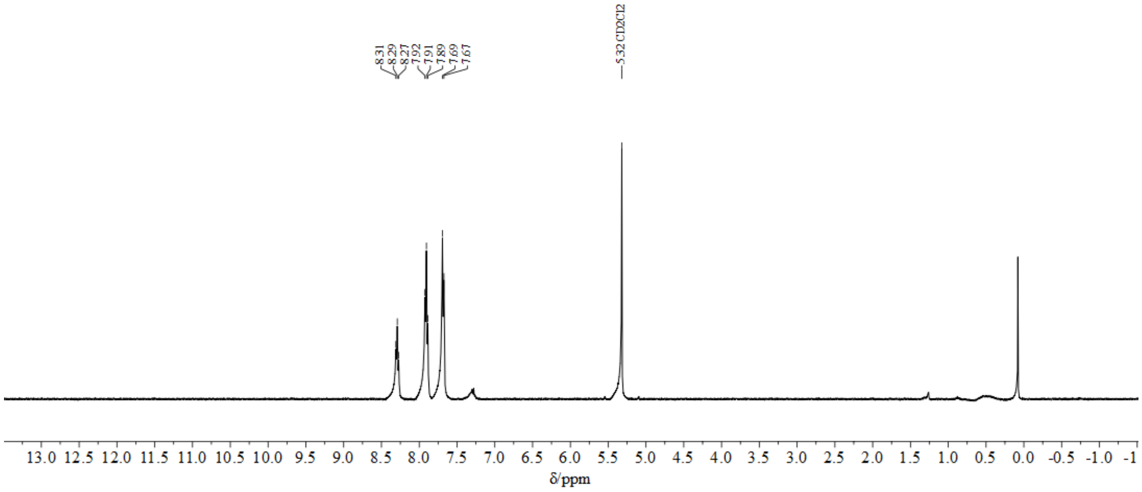

Figure S37. ^{1}H NMR spectrum (400 MHz, CD_2Cl_2, 22 °C) of [Ph_3C][(F_5TeO)Al(OTe^R)$_3$] (**10**).

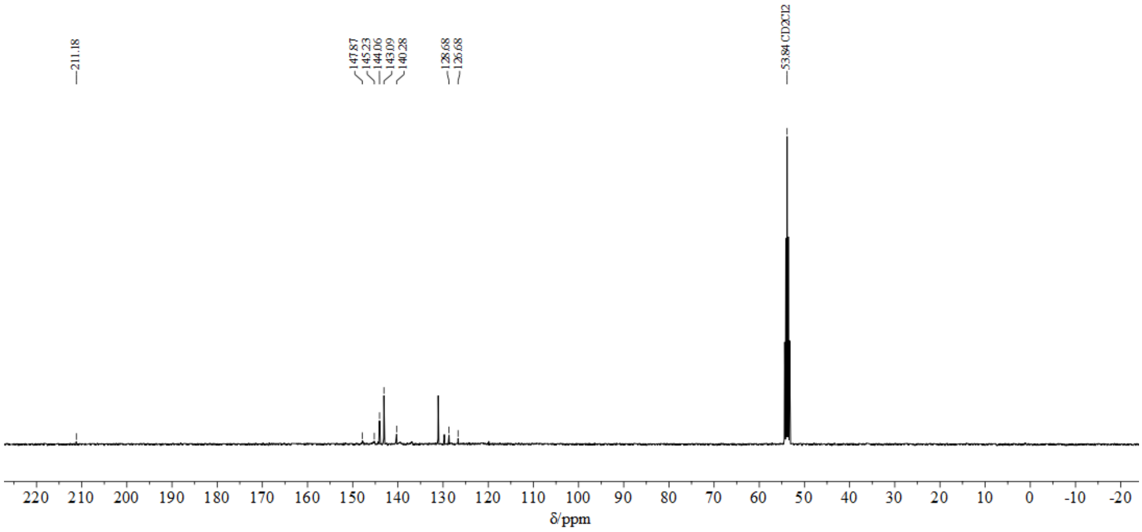

Figure S38. ^{13}C{^{1}H} NMR spectrum (100 MHz, CD_2Cl_2, 22 °C) of [Ph_3C][(F_5TeO)Al(OTe^R)$_3$] (**10**).

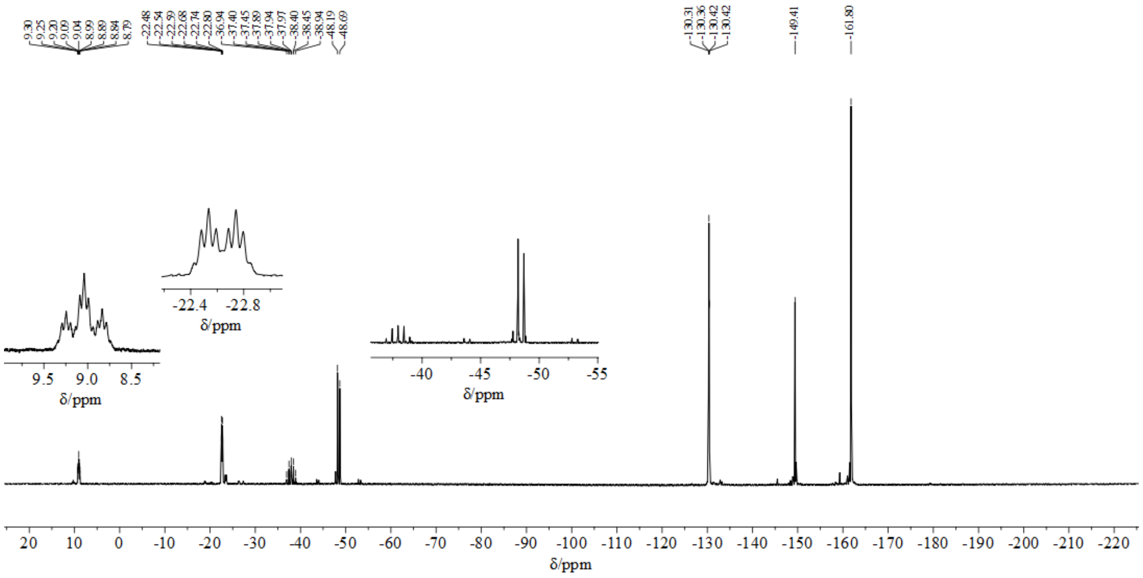

Figure S39. ^{19}F NMR spectrum (377 MHz, CD_2Cl_2, 22 °C) of $[Ph_3C][(F_5TeO)Al(OTe^R)_3]$ (**10**).

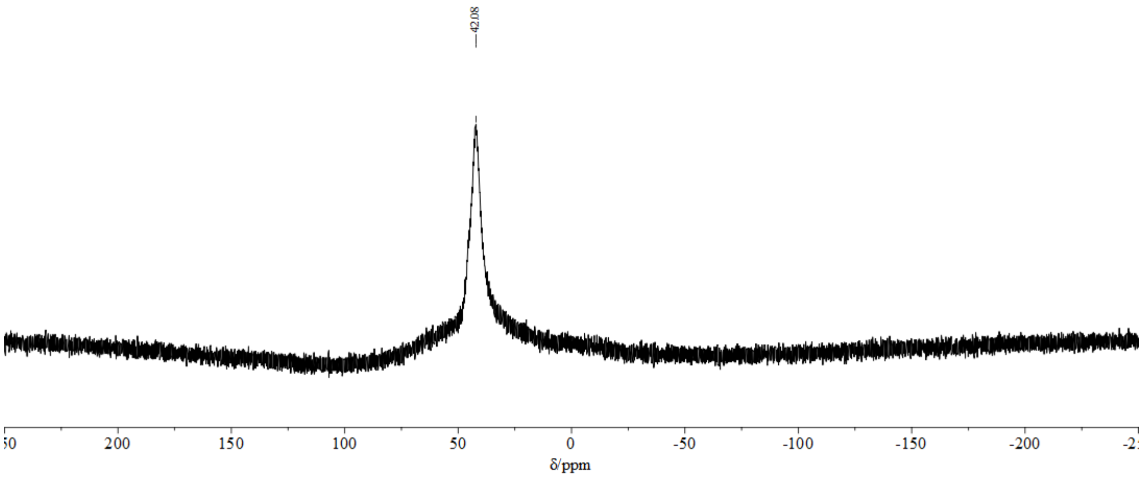

Figure S40. ^{27}Al NMR spectrum (128 MHz, CD_2Cl_2, 22 °C) of $[Ph_3C][(F_5TeO)Al(OTe^R)_3]$ (**10**).

3 Thermogravimetric analysis

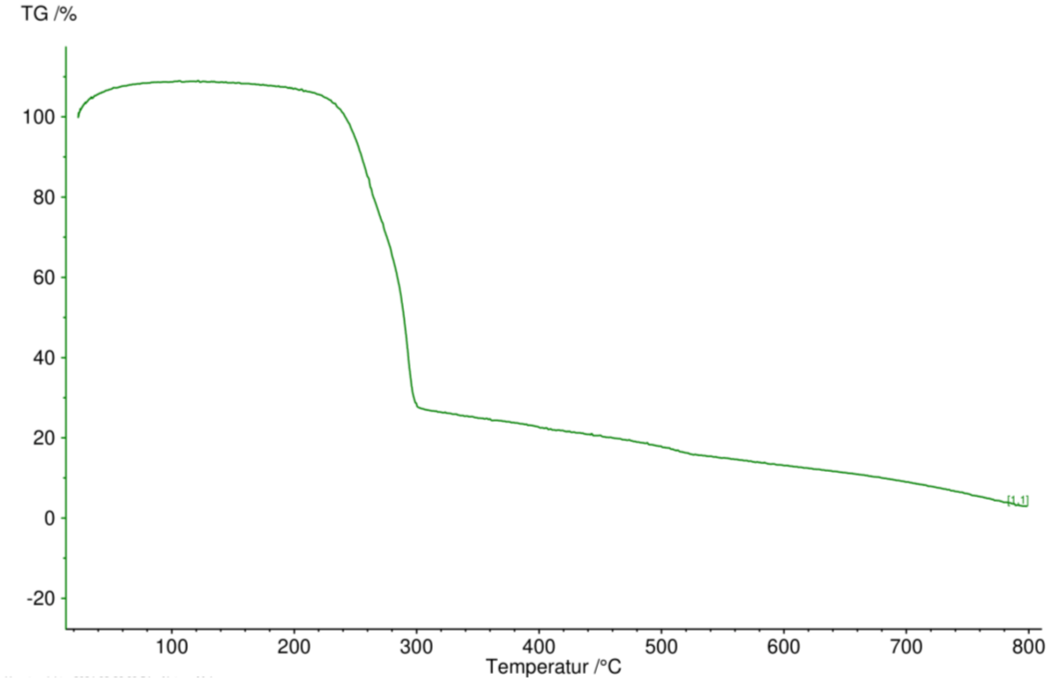

Figure S41. TGA of compound K[FAl(OTe^R)$_3$] (**5**).

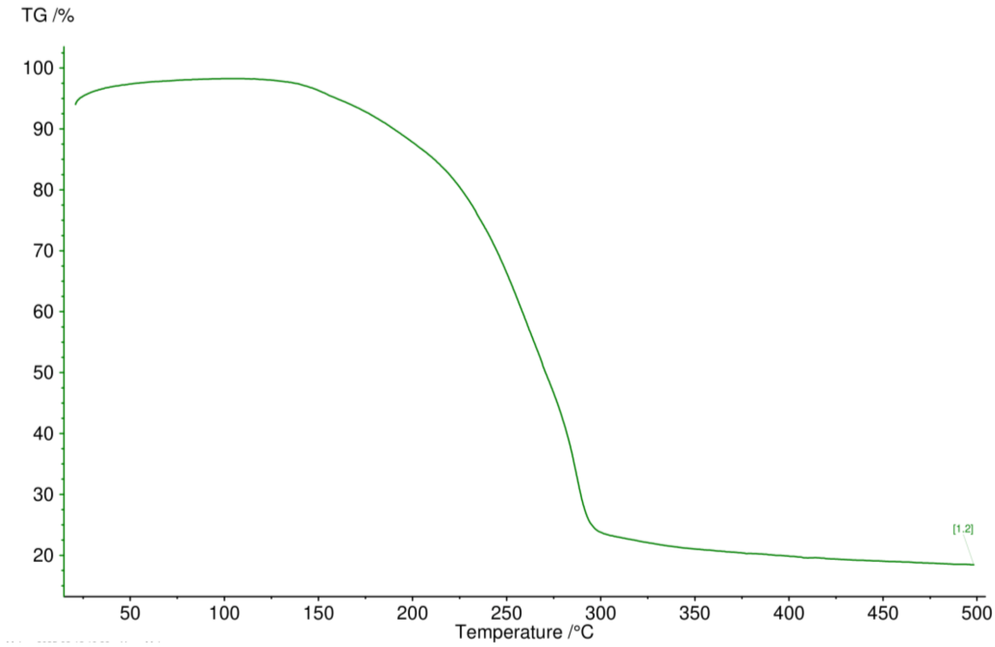

Figure S42. TGA of compound Ag[(F_5TeO)Al(OTe^R)$_3$] (**9**).

4 Crystal data

Table S1. Crystal data and structure refinement.

Empirical formula	$C_{38}H_5AlF_{40}KO_{3.50}Te_3$	$C_{43}H_{18}AlF_{40}NO_3Te_3$	$C_{40}H_{10}AlCsF_{40}O_4Te_3$
Formula weight	1726.30	1766.36	1857.17
Temperature/K	100.00	100.00	100.00
Crystal system	monoclinic	orthorhombic	monoclinic
Space group	$P2_1/c$	$Pna2_1$	$P2_1$
a/Å	21.3565(12)	21.697(2)	10.3516(5)
b/Å	13.3196(8)	11.9961(11)	18.6311(9)
c/Å	36.9245(17)	20.0956(19)	13.3324(5)
α/°	90	90	90
β/°	93.076(2)	90	96.832(2)
γ/°	90	90	90
Volume/Å^3	10488.4(10)	5230.6(8)	2553.1(2)
Z	8	4	2
ρ_{calc}g/cm^3	2.186	2.243	2.416
μ/mm^{-1}	1.935	1.865	2.612
F(000)	6472	3344	1732
Crystal size/mm^3	0.138 × 0.184 × 0.207	0.128 × 0.237 × 0.446	0.247 × 0.269 × 0.322
Radiation	MoKα (λ = 0.71073)	MoKα (λ = 0.71073)	MoKα (λ = 0.71073)
2Θ range for data collection/°	4.28 to 50.75	3.88 to 50.77	4.53 to 56.62
Index ranges	$-25 \le h \le 25$, $-16 \le k \le 13$, $-39 \le l \le 44$	$-26 \le h \le 26$, $-14 \le k \le 14$, $-24 \le l \le 23$	$-13 \le h \le 13$, $-24 \le k \le 24$, $-17 \le l \le 16$
Reflections collected	152256	86038	49024
Independent reflections	18992 [R_{int} = 0.0732, R_{sigma} = 0.0367]	9504 [R_{int} = 0.0494, R_{sigma} = 0.0288]	11418 [R_{int} = 0.0442, R_{sigma} = 0.0446]
Data/restraints/parameters	18992/2590/2074	9604/620/738	11418/1/778
Goodness-of-fit on F^2	1.050	1.154	1.032
Final R indexes [I>=2σ (I)]	R_1 = 0.0869, wR_2 = 0.2117	R_1 = 0.0455, wR_2 = 0.1007	R_1 = 0.0261, wR_2 = 0.0439
Final R indexes [all data]	R_1 = 0.1144, wR_2 = 0.2345	R_1 = 0.0515, wR_2 = 0.1066	R_1 = 0.0331, wR_2 = 0.0464
Largest diff. peak/hole / e Å^{-3}	2.70/−1.59	1.37/−1.78	0.93/−0.71
CCDC number	2448090	2448091	2448092

Table S1. Crystal data and structure refinement.

Empirical formula	$C_{42}H_{15}AlF_{39}O_4PTe_3$	$C_{45}H_{22}AlCl_2F_{26}O_3PTe_2$	$C_{32}H_{20}F_{26}GaO_3PTe_2$
Formula weight	1765.29	1488.67	1302.37
Temperature/K	100.00	150.00	100.00
Crystal system	monoclinic	triclinic	triclinic
Space group	$P2_1/n$	$P\bar{1}$	$P\bar{1}$
a/Å	11.1820(6)	13.558(3)	11.2102(5)
b/Å	18.9627(10)	13.978(2)	11.7163(5)
c/Å	24.1286(13)	14.678(3)	16.7637(7)
α/°	90	103.825(4)	90.850(2)
β/°	94.769(2)	92.778(5)	103.082(2)
γ/°	90	105.521(6)	113.986(2)
Volume/Å^3	5098.5(5)	2583.7(9)	1945.26(15)
Z	4	2	2
ρ_{calc}g/cm^3	2.300	1.914	2.223
μ/mm^{-1}	1.942	1.415	2.380
F(000)	3336	1432	1240
Crystal size/mm^3	0.108 × 0.236 × 0.531	0.11 × 0.128 × 0.425	0.148 × 0.201 × 0.424
Radiation	MoKα (λ = 0.71073)	MoKα (λ = 0.71073)	MoKα (λ = 0.71073)
2Θ range for data collection/°	4.30 to 50.70	4.00 to 50.81	4.11 to 52.78
Index ranges	$-11 \leq h \leq 13, -20 \leq k \leq 22, -28 \leq l \leq 29$	$-16 \leq h \leq 16, -16 \leq k \leq 15, -17 \leq l \leq 17$	$-14 \leq h \leq 14, -14 \leq k \leq 14, -20 \leq l \leq 20$
Reflections collected	52640	56957	69401
Independent reflections	9320 [R_{int} = 0.0249, R_{sigma} = 0.0183]	9514 [R_{int} = 0.0608, R_{sigma} = 0.0364]	7950 [R_{int} = 0.0328, R_{sigma} = 0.0166]
Data/restraints/parameters	9320/0/814	9514/13/706	7950/352/758
Goodness-of-fit on F^2	1.264	1.090	1.130
Final R indexes [I>=2σ (I)]	R_1 = 0.0312, wR_2 = 0.0625	R_1 = 0.0428, wR_2 = 0.0857	R_1 = 0.0258, wR_2 = 0.0604
Final R indexes [all data]	R_1 = 0.0384, wR_2 = 0.0683	R_1 = 0.0595, wR_2 = 0.0989	R_1 = 0.0294, wR_2 = 0.0634
Largest diff. peak/hole / e Å^{-3}	0.83/−0.81	1.65/−1.15	2.00/−1.31
CCDC number	2448093	2448094	2448095

Table S1. Crystal data and structure refinement.

Empirical formula	$C_{44}H_{20}AlF_{44}NO_4Te_4$	$C_{43.75}H_{17}AlCl_2F_{39}O_6Te_3$	$C_{76.25}H_{46}AgAlF_{44}O_4Te_4$
Formula weight	1999.99	1860.25	2507.38
Temperature/K	100.00	100.00	100.00
Crystal system	monoclinic	monoclinic	monoclinic
Space group	$C2/c$	$P2_1/n$	$P2_1/n$
a/Å	18.9759(8)	27.9447(15)	15.0417(7)
b/Å	15.1917(6)	10.2052(5)	31.4261(15)
c/Å	39.7873(15)	37.969(2)	19.9122(9)
α/°	90	90	90
β/°	92.363(2)	90.872(2)	105.079(2)
γ/°	90	90	90
Volume/Å^3	11460.0(8)	10826.7(10)	9088.4(7)
Z	8	8	4
ρ_{calc}g/cm^3	2.318	2.283	1.832
μ/mm^{-1}	2.220	1.905	1.625
F(000)	7520	7052	4798
Crystal size/mm^3	0.108 × 0.129 × 0.187	0.035 × 0.056 × 0.183	0.172 × 0.208 × 0.589
Radiation	MoKα (λ = 0.71073)	MoKα (λ = 0.71073)	MoKα (λ = 0.71073)
2Θ range for data collection/°	4.05 to 52.79	4.13 to 50.86	4.00 to 52.80
Index ranges	$-23 \leq h \leq 23$, $-18 \leq k \leq 18$, $-49 \leq l \leq 49$	$-31 \leq h \leq 33$, $-12 \leq k \leq 11$, $-45 \leq l \leq 45$	$-18 \leq h \leq 18$, $-39 \leq k \leq 39$, $-24 \leq l \leq 24$
Reflections collected	405382	319143	167918
Independent reflections	11728 [R_{int} = 0.0475, R_{sigma} = 0.0108]	19068 [R_{int} = 0.1197, R_{sigma} = 0.0771]	18604 [R_{int} = 0.0474, R_{sigma} = 0.0264]
Data/restraints/parameters	11728/0/887	19068/18/1235	18604/900/1344
Goodness-of-fit on F^2	1.246	1.131	1.124
Final R indexes [I>=2σ (I)]	R_1 = 0.0270, wR_2 = 0.0540	R_1 = 0.0722, wR_2 = 0.1249	R_1 = 0.0602, wR_2 = 0.1537
Final R indexes [all data]	R_1 = 0.0366, wR_2 = 0.0635	R_1 = 0.1046, wR_2 = 0.1344	R_1 = 0.705, wR_2 = 0.1625
Largest diff. peak/hole / e Å^{-3}	1.01/−0.91	1.32/−1.58	1.74/−1.82
CCDC number	2448096	2448097	2448098

Table S1. Crystal data and structure refinement.

Empirical formula	$C_{80.62}H_{44.44}AlF_{44}O_4Te_4$
Formula weight	2450.42
Temperature/K	100.00
Crystal system	triclinic
Space group	$P\overline{1}$ (2)
a/Å	14.4624(5)
b/Å	15.0180(6)
c/Å	21.2258(8)
α/°	75.5510(10)
β/°	70.5070(10)
γ/°	78.3460(10)
Volume/Å^3	4172.7(3)
Z	2
ρ_{calc}g/cm^3	1.950
μ/mm^{-1}	1.544
F(000)	2354
Crystal size/mm^3	0.068 × 0.087 × 0.403
Radiation	MoKα (λ = 0.71073)
2Θ range for data collection/°	4.15 to 50.70
Index ranges	$-16 \leq h \leq 17$, $-18 \leq k \leq 18$, $-25 \leq l \leq 25$
Reflections collected	122253
Independent reflections	15276 [R_{int} = 0.058, R_{sigma} = 0.0297]
Data/restraints/parameters	15276/62/1174
Goodness-of-fit on F^2	1.130
Final R indexes [I>=2σ (I)]	R_1 = 0.0787, wR_2 = 0.1899
Final R indexes [all data]	R_1 = 0.1091, wR_2 = 0.2327
Largest diff. peak/hole / e Å^{-3}	2.69/−2.95
CCDC number	2454862

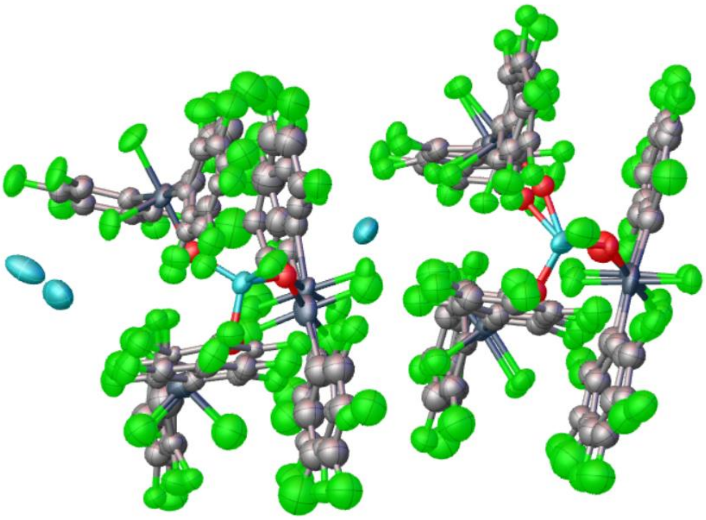

Figure S43. Representation of the solid-state structure of K[FAl(OTe^R)$_3$] (**5**). The unit cell contains two [FAl(OTe^R)$_3$]$^-$ anions and two potassium cations. One K$^+$ cation is disordered (occupation 0.25% and 0.75%) and all OTe^R groups are disordered. Displacement ellipsoids set at 50% probability. The summary of crystal data and structure refinement appears in Table S1.

5 Powder diffractometry

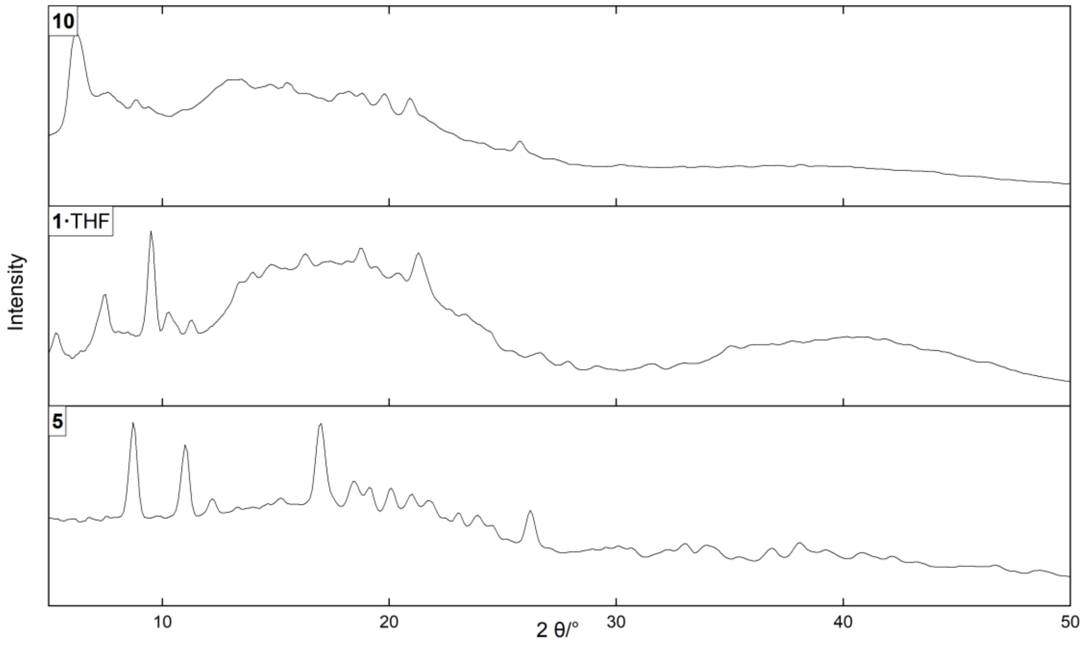

Figure S44. Powder diffraction pattern of compounds **10, 1**·THF and **5**.

6 Quantum-chemical calculations

Quantum chemical calculations were performed using the *Turbomole V7.6* program,[9] with the BP86[10–12] or PBEh-3c[13] functional (with RI[14]) in conjunction with basis sets def-SV(P) or def2-mSVP and D3BJ dispersion correction.[13,15,16] Minima on potential energy surfaces were characterized by normal mode analysis. Thermochemical data are provided without counterpoise correction but including zero-point energy correction as obtained from harmonic vibrational frequencies. Single point calculations where performed on the PW6B95[17]-(D4)/def2-QZVPP[15,18] level of theory based on the PBEh-3c structures. Buried volume calculations were performed using the SambVca 2.1 routine on .xyz coordinates from the BP86-D3BJ/def2-SVP geometry optimizations according to Radius et al.[19] The F atom was selected as the centre of the sphere, Z axis was defined by Al, the xy plane was defined by an oxygen atom bound to aluminum.

% V_{bur} determination

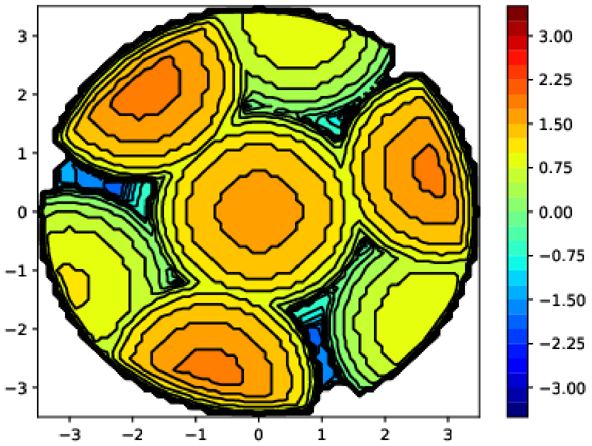

Figure S45. %V_{bur} of $[FAl(OTe^R)_3]^-$ (69.1%).

Table S2. Details on FIA calculations

Level	Compound	SCF-Energy/kJmol^{-1}	ZPE/kJmol^{-1}
BP86/def-SV(P)	Al(OTeR)3	-15106529	846,814613
	FAl(OTeR)3	-15368959,5	851,090664
PBEh-3c/def2-mSVP	Al(OTeR)3	-17135123,1	920,764281
	FAl(OTeR)3	-17397289,7	925,879684
PW6b95-D4/def2-QZVPP	Al(OTeR)3	-17189743	
	FAl(OTeR)3	-17452827,9	

Structures optimized at the BP86-D3(BJ)/def-SV(P) level of theory

Al(OTe^{R})$_3$

82			
O	-1.7202760	0.3211967	0.8654435
Al	-0.2731685	1.2286353	0.5402827
Te	-3.5747348	0.2841808	1.4202457
F	-5.4488510	0.1234802	1.9655967
F	-3.2933470	1.6391368	2.7977908
F	-3.9200256	-1.0439979	0.0170484
O	-0.0741293	2.7964082	1.2478165
Te	1.1481755	4.0066974	2.1298070
O	1.1820484	0.1594657	0.3554883
Te	1.4015801	-0.0428663	-1.5657836
F	2.7891796	-1.3791213	-1.4935472
F	2.2906741	5.3276922	3.0074724
F	0.3502269	3.5997957	3.8597840
F	2.0684936	4.3928242	0.4298540
F	-0.2191064	1.4484366	-1.3108609
F	1.4460091	0.0041629	-3.5240799
F	-2.6064797	-4.1633681	5.7574113
C	-2.7936795	-3.2361212	4.8217756
C	-3.7965788	-3.4118962	3.8527420
F	-4.5565922	-4.5074180	3.8728947
C	-3.9999676	-2.4286899	2.8666771
F	-4.9690055	-2.6348024	1.9806257
C	-3.1927327	-1.2758260	2.8407871
C	-2.1888500	-1.1096176	3.8112290
F	-1.4033665	-0.0350800	3.8306315
C	-1.9848890	-2.0867810	4.8021799
F	-1.0342451	-1.9232358	5.7224661
F	-3.0729696	0.9086098	-1.7654950
C	-3.9181485	1.8165691	-1.2697083
C	-4.2690327	1.8283105	0.0904508
C	-5.1675471	2.8052667	0.5621619
F	-5.5519047	2.8638386	1.8319079
C	-5.6936491	3.7672908	-0.3207914
F	-6.5424405	4.6924103	0.1286523
C	-5.3330192	3.7468189	-1.6793646
F	-5.8337723	4.6530808	-2.5143668
C	-4.4363794	2.7755282	-2.1571492
F	-4.0897999	2.7612388	-3.4442359
F	-0.4981119	5.0328115	-0.5143449
C	-0.8147490	5.7973006	0.5312962
C	-0.2387925	5.6055050	1.7998273
C	-0.6021512	6.4528301	2.8634040

F	-0.0818920	6.3235814	4.0788628
C	-1.5547195	7.4704592	2.6660572
F	-1.9061583	8.2639015	3.6781161
C	-2.1346314	7.6486631	1.3985997
F	-3.0376494	8.6075406	1.2096777
C	-1.7665318	6.8129030	0.3296735
F	-2.3187832	6.9864345	-0.8735582
F	1.0509828	0.9069719	3.1120767
C	2.3342074	1.2818026	3.0187386
C	3.3144640	0.3425524	3.3770661
F	2.9643645	-0.8732757	3.7943497
C	4.6716812	0.6993959	3.2949858
F	5.6110518	-0.1776344	3.6396260
C	5.0411307	1.9766632	2.8386701
C	4.0494491	2.9028619	2.4656006
F	4.4530494	4.0911283	2.0216748
C	2.6873097	2.5598972	2.5578731
F	4.1982503	0.6499861	-0.1188318
C	3.8659505	1.6771068	-0.9041273
C	2.7110887	1.6471116	-1.7036019
C	2.3755180	2.7474130	-2.5117784
F	1.2808273	2.7586138	-3.2648506
C	3.2034019	3.8837639	-2.5166510
F	2.8829710	4.9389252	-3.2613247
C	4.3657655	3.9088554	-1.7265051
F	5.1456621	4.9820312	-1.7344445
C	4.7072277	2.8006454	-0.9318389
F	5.8172316	2.8192473	-0.1945124
F	-1.6580199	-4.1900266	0.1272627
C	-1.5089200	-3.2458948	-0.8016353
C	-2.5001085	-3.0604483	-1.7805343
F	-3.5775795	-3.8329524	-1.7815155
C	-2.3521818	-2.0651752	-2.7609773
F	-3.3014100	-1.8876688	-3.6766966
C	-1.2203938	-1.2315511	-2.7518983
F	-1.1533770	-0.2603676	-3.6551800
C	-0.2213132	-1.4261395	-1.7790856
C	-0.3701222	-2.4251988	-0.7980197
F	0.5532334	-2.6230536	0.1405044
F	6.3302181	2.3054403	2.7604898

$[FAl(OTe^R)_3]^-$

83

Te	1.0176911	7.5590005	8.4057589
F	2.8014438	6.7619823	8.5337619
F	0.9606166	6.8529142	6.5706401

F	-0.7744141	8.2969805	8.0933974
O	1.0767152	8.3256117	10.1644018
C	1.8232893	9.3604211	7.5339691
C	0.0899708	5.7008770	8.9852421
Al	1.8567423	7.7949372	11.7003696
F	3.5357537	7.8906370	11.6505376
C	2.9439608	9.9735072	8.1230254
C	1.2097522	9.9684593	6.4234676
C	-1.0347020	5.7208506	9.8275452
C	0.5924724	4.4698344	8.5324621
O	1.2366310	6.1299452	11.9640274
O	1.1397444	8.7908295	13.0193083
F	3.5777533	9.4308191	9.1558447
C	3.4293692	11.2008952	7.6320056
F	0.1623236	9.4278819	5.8038951
C	1.6886290	11.1958160	5.9289697
F	-1.5370394	6.8585506	10.2988501
C	-1.6681210	4.5239066	10.2052129
F	1.6551800	4.3830694	7.7310269
C	-0.0272629	3.2689070	8.9210029
Te	1.3098478	4.8622654	13.3977751
F	-0.4714234	4.1803858	12.9347422
F	3.0967401	5.4039707	13.9876996
Te	1.0511363	10.6813083	13.3389216
F	2.7911161	11.0401444	12.5188356
F	-0.6822666	10.5109594	14.2395796
F	3.7160511	9.1052912	14.3741944
F	4.4904513	11.7839898	8.1961108
C	2.7986429	11.8075523	6.5338758
F	1.0945530	11.7814831	4.8822316
F	-2.7476318	4.5471508	10.9863958
C	-1.1545911	3.2973563	9.7574334
F	0.4541034	2.0911059	8.4924903
F	1.3644135	3.5003191	14.8132164
C	0.4096429	6.1670922	14.8578321
C	2.1635429	3.3671579	12.0986281
F	0.9542785	12.6105747	13.7056761
C	1.9781876	10.4785411	15.2789025
C	0.0338723	11.1013624	11.4870905
C	3.1377940	9.6931374	15.4150680
F	3.2555888	12.9702499	6.0623249
F	-1.7387741	2.1538064	10.1219347
C	-0.7571677	6.8785608	14.5288858
C	1.0155069	6.3730232	16.1090131
C	3.2675473	3.7039470	11.2942822
C	1.6131899	2.0764212	12.0046760
C	1.4261523	11.0884096	16.4205111

C	0.5400980	12.0482852	10.5812200
C	-1.0934888	10.3406354	11.1320807
C	3.7290918	9.4985710	16.6784296
F	-1.3657068	6.7227635	13.3568231
C	-1.3189856	7.7950377	15.4359646
F	2.1048667	5.7073306	16.4926732
C	0.4778320	7.3115925	17.0067236
F	3.8436292	4.8983680	11.3555633
C	3.8025049	2.7698707	10.3864682
F	0.5789943	1.6888498	12.7506938
C	2.1397371	1.1397520	11.0969094
F	0.3433546	11.8613033	16.3647103
C	2.0082856	10.8932258	17.6867584
F	1.5903320	12.8183502	10.8668577
C	-0.0578836	12.2059554	9.3187380
F	-1.6036865	9.4206954	11.9466288
C	-1.7140995	10.5157699	9.8830621
F	4.8318536	8.7544818	16.8003422
C	3.1605193	10.1005554	17.8121979
F	-2.4412492	8.4453361	15.1331473
C	-0.6859006	8.0207754	16.6677371
F	1.0665195	7.5277841	18.1936183
F	4.8534532	3.0904441	9.6279165
C	3.2325754	1.4906766	10.2882707
F	1.6057464	-0.0833689	10.9991859
F	1.4731930	11.4668225	18.7714630
F	0.4364278	13.0946149	8.4420814
C	-1.1818953	11.4396724	8.9715615
F	-2.7947662	9.8061265	9.5617892
F	3.7174157	9.9233116	19.0127266
F	-1.1895160	8.9094122	17.5282054
F	3.7336355	0.6020629	9.4267375
F	-1.7429108	11.5983659	7.7710054

$Al(OC(CF_3)_3)_3$

43

Al	0.1354822	-0.0216357	0.1311648
O	1.7941116	0.0869497	0.7117025
O	-0.8059695	1.4092695	0.1314912
O	-0.4567934	-1.6240105	-0.2855508
C	-0.9789166	-2.5342969	0.5984311
C	2.8923702	0.2596482	-0.0907100
C	-1.7788476	2.2219140	-0.3769725
C	-0.3506852	-3.9476082	0.3030398
F	0.9793748	-3.8399741	0.1652294
F	-0.8497233	-4.4566515	-0.8334199

F	-0.6101587	-4.8110033	1.3092552
C	-0.6250567	-2.1073158	2.0816353
F	-1.5498552	-2.3673247	2.9914103
F	-0.4850576	-0.6846339	2.0515132
F	0.5497952	-2.5635910	2.5000513
C	-2.5443808	-2.5791240	0.4202015
F	-2.8590667	-2.6557034	-0.8759352
F	-3.0791471	-1.4409793	0.9158591
F	-3.0987252	-3.6230143	1.0651209
C	3.3212097	1.7760644	-0.0670467
F	2.4169869	2.5061928	-0.7610748
F	4.5323047	1.9664619	-0.6233859
F	3.3440340	2.2306008	1.1887043
C	4.0632475	-0.6515286	0.4384205
F	5.0418727	-0.7605566	-0.4876665
F	3.6021537	-1.8792692	0.7163916
F	4.5933503	-0.1376160	1.5586508
C	2.5359513	-0.1668102	-1.5692860
F	1.1382198	0.1210077	-1.7244207
F	2.6447802	-1.4724782	-1.7832863
F	3.1567342	0.4950774	-2.5304491
C	-2.8137225	2.5483547	0.7684361
F	-3.9538562	3.0642561	0.2711699
F	-2.2945464	3.4330415	1.6404277
F	-3.1142903	1.4311262	1.4518048
C	-1.1043849	3.5554268	-0.8816551
F	-2.0136091	4.5243633	-1.0939699
F	-0.4470355	3.3292055	-2.0410957
F	-0.2124379	3.9958754	0.0151950
C	-2.5203905	1.5179983	-1.5771379
F	-1.6166404	0.8543704	-2.3344993
F	-3.1726167	2.3886197	-2.3628264
F	-3.4060653	0.6093008	-1.1249176

$[FAl(OC(CF_3)_3)_3]^-$

44

Al	0.1201370	0.2883225	0.7995408
O	1.5807297	-0.2223075	-0.0767400
O	-0.5627333	1.6494368	-0.1107035
O	-1.0536110	-1.0405625	0.6862461
C	-1.2406686	-2.3680741	0.8310758
C	2.8657027	0.0872132	-0.3463555
C	-1.6581124	2.3269478	-0.5011980
C	-0.6711248	-3.1480531	-0.4215521
F	0.6688905	-3.2801361	-0.3410690
F	-0.9507083	-2.4800624	-1.5506793

F	-1.1941929	-4.3964688	-0.5390071
C	-0.5463605	-2.9172857	2.1379874
F	-0.4412344	-4.2694674	2.1440653
F	-1.2403671	-2.5614197	3.2406548
F	0.6916000	-2.4084408	2.2568136
C	-2.7939143	-2.6284939	0.9349886
F	-3.3851966	-2.4871210	-0.2708147
F	-3.3638797	-1.7508269	1.7740877
F	-3.0830099	-3.8771396	1.3837255
C	3.0649034	1.6419819	-0.5354262
F	2.5680946	2.0558056	-1.7192950
F	4.3698546	2.0096177	-0.4926683
F	2.4188833	2.3106580	0.4353027
C	3.8116797	-0.4110126	0.8194515
F	5.1135647	-0.4815546	0.4382688
F	3.4395932	-1.6314834	1.2334876
F	3.7488022	0.4187050	1.8826730
C	3.2724380	-0.6461948	-1.6833456
F	2.3140921	-0.5028531	-2.6099553
F	3.4449283	-1.9698178	-1.4738502
F	4.4294770	-0.1654559	-2.2048596
C	-2.4880209	2.8396304	0.7392931
F	-3.7277768	3.2689126	0.3940173
F	-1.8585953	3.8678326	1.3486246
F	-2.6303056	1.8593547	1.6475257
C	-1.1722887	3.5699037	-1.3461919
F	-2.1486113	4.4947060	-1.5278894
F	-0.7527181	3.1775460	-2.5689219
F	-0.1430733	4.1800006	-0.7398489
C	-2.5955409	1.4311165	-1.4089667
F	-1.8567505	0.6661951	-2.2250030
F	-3.4304009	2.1756729	-2.1802970
F	-3.3665023	0.6166369	-0.6596365
F	0.4323274	0.7080355	2.4064447

$Al(OC(C_6F_5)_3)_3$

106

C	-0.0071794	1.3475670	-3.3061095
C	-1.0464747	0.2127641	-3.0782850
C	-1.8591729	-0.1534295	-4.1720248
F	-1.7731141	0.4931470	-5.3472275
C	-2.7958967	-1.1943209	-4.0943107
F	-3.5223016	-1.5251429	-5.1654401
C	-2.9817442	-1.8729645	-2.8805350
F	-3.8711843	-2.8612102	-2.7898155
C	-2.2107755	-1.5116309	-1.7663024

F	-2.3756497	-2.1500480	-0.6048493
C	-1.2733891	-0.4699127	-1.8687366
F	-0.5826817	-0.1840681	-0.7543038
F	3.6197828	-0.9563492	-0.9683452
C	2.9720381	0.1914726	-1.1710437
C	1.8390616	0.2286785	-1.9886079
F	1.4671167	-0.9288008	-2.5607817
C	1.1013414	1.4056353	-2.2298404
C	1.6030036	2.5832507	-1.6486553
F	0.9978632	3.7708815	-1.8112670
C	2.7603677	2.5859989	-0.8470357
F	3.2153006	3.7276403	-0.3274853
C	3.4565500	1.3909866	-0.6221673
F	4.5718183	1.3949533	0.1091028
F	-0.3629553	3.1841824	-5.6846689
C	-1.0246972	3.4213507	-4.5419491
C	-1.9055705	4.5194816	-4.5758233
F	-2.0558957	5.2113398	-5.7097296
C	-2.6200855	4.8946735	-3.4313908
F	-3.4351292	5.9470811	-3.4574109
C	-2.4703250	4.1395805	-2.2553096
F	-3.1450616	4.4758412	-1.1535802
C	-1.6135995	3.0324931	-2.2562953
F	-1.4912205	2.3432554	-1.1096056
C	-0.8598003	2.6430743	-3.3825477
O	0.6431360	1.0625908	-4.4930296
F	6.4418403	1.8773747	-9.0128944
F	4.9243937	1.6975698	-6.8167822
C	5.8829254	2.9831041	-8.5133667
C	5.0764975	2.9119927	-7.3696868
F	5.5507627	0.4351679	-2.5507825
F	7.4102515	2.2598583	-1.6831703
F	6.9031776	4.3174751	-10.1894730
C	5.5192241	1.6930998	-2.9943362
C	6.4748580	2.6204290	-2.5568976
C	6.1324783	4.2322095	-9.1053492
C	4.5252975	2.1039344	-3.8954563
C	6.4374220	3.9342101	-3.0602339
F	3.6011101	1.1289560	-4.1986496
O	2.6428731	2.8011231	-5.9707818
F	7.3334281	4.8287304	-2.6446147
C	4.4720286	3.3878123	-4.4489201
C	5.4609087	4.2872658	-4.0015147
C	4.4589948	4.0494610	-6.8051837
C	3.5122217	3.8060144	-5.5954109
F	5.4681509	5.5405148	-4.4743376
C	5.5627524	5.3866481	-8.5455349

F	1.8658156	5.0475482	-7.5038147
C	4.7583049	5.2893170	-7.3950702
C	2.5864533	4.9882743	-5.2299546
F	5.7964897	6.5800774	-9.0980529
C	1.7138395	5.4547512	-6.2370109
F	4.2698587	6.4372067	-6.9060841
F	3.0862317	5.0477420	-2.8780806
C	2.3273904	5.4294225	-3.9215565
C	0.6659232	6.3445071	-5.9729006
F	-0.1155666	6.7797201	-6.9614449
C	1.2573615	6.2919385	-3.6209814
C	0.4097116	6.7312095	-4.6460797
F	1.0276701	6.6623651	-2.3587988
F	-0.6269123	7.5218960	-4.3633487
F	4.1001034	-3.6851867	-3.1474935
F	4.2626166	-2.2341703	-5.4073858
C	2.9858163	-3.3770201	-3.8152566
C	3.0490570	-2.6239793	-4.9940903
F	4.9018289	0.1167220	-10.6564446
F	5.9911829	-2.3869313	-10.9413249
F	1.6620505	-4.4957607	-2.1931199
C	4.5243635	-0.8765101	-9.8471087
C	5.0701432	-2.1608529	-10.0044297
C	1.7371550	-3.7905423	-3.3214506
C	3.5558975	-0.6544556	-8.8583191
C	4.6338121	-3.2028757	-9.1717953
F	3.0335877	0.5822352	-8.7993806
O	2.5928378	-0.1325110	-6.2923365
F	5.1424151	-4.4293792	-9.3194495
C	3.1188919	-1.6755946	-7.9850270
C	3.6560663	-2.9576626	-8.1898496
C	1.9003827	-2.2821337	-5.7415246
C	2.0869029	-1.2699300	-6.8975563
F	3.2800691	-4.0056669	-7.4432755
C	0.5762202	-3.4472941	-4.0242865
F	0.6966677	1.3744081	-7.2302297
C	0.6664576	-2.6870496	-5.2035771
C	0.7766096	-1.0130491	-7.6872511
F	-0.6243738	-3.8132267	-3.5555160
C	0.1821973	0.2436149	-7.8336097
F	-0.5099123	-2.3643842	-5.7774356
F	0.6633925	-3.3033311	-8.2957006
C	0.1544244	-2.0664525	-8.3856896
C	-0.9869916	0.4718705	-8.5729805
F	-1.5109294	1.6932071	-8.6560835
C	-1.0146202	-1.8882551	-9.1376512
C	-1.5873159	-0.6070753	-9.2388398

F	-1.5869384	-2.9207158	-9.7569139
F	-2.6980284	-0.4220535	-9.9491502
Al	1.9461766	1.2929427	-5.5795129

$[FAl(OC(C_6F_5)_3)_3]^-$

107

C	0.0977189	1.1852480	-2.7908533
C	-1.0983225	0.2046414	-2.5675184
C	-2.2789605	0.4817606	-3.2887993
F	-2.3764720	1.5785255	-4.0508724
C	-3.4107451	-0.3440381	-3.2187130
F	-4.5265750	-0.0348683	-3.8891324
C	-3.3756494	-1.5002685	-2.4251611
F	-4.4438688	-2.3056920	-2.3613344
C	-2.2236425	-1.7920205	-1.6828209
F	-2.1935865	-2.8810489	-0.8999375
C	-1.1119723	-0.9331730	-1.7461659
F	-0.0709050	-1.2563532	-0.9603662
F	3.7739604	-2.0618421	-2.7886324
C	3.2940977	-0.8998145	-2.3312919
C	2.0013357	-0.4789996	-2.6606411
F	1.2810041	-1.2809885	-3.4576383
C	1.4330800	0.7091568	-2.1491290
C	2.2243595	1.4303813	-1.2379156
F	1.7838867	2.5252402	-0.5960056
C	3.5469447	1.0559193	-0.9370334
F	4.2847434	1.8138478	-0.1132966
C	4.0895693	-0.1086271	-1.4877040
F	5.3375403	-0.4831183	-1.1886071
F	0.0971943	3.8602920	-4.1761035
C	-0.3735782	3.7522359	-2.9300493
C	-0.8846912	4.9442570	-2.3808816
F	-0.8719297	6.0698792	-3.1056747
C	-1.3985455	4.9614318	-1.0782192
F	-1.8794318	6.0964814	-0.5554126
C	-1.4097926	3.7750888	-0.3266643
F	-1.8797704	3.7797560	0.9300818
C	-0.8985308	2.6030106	-0.8958633
F	-0.8755247	1.5027110	-0.1207190
C	-0.3815979	2.5473933	-2.2038960
O	0.2851518	1.2441527	-4.1403521
F	7.0550141	0.4991098	-4.4333228
F	4.8486589	1.8801378	-3.8254742
C	6.5552617	1.3626130	-5.3269934
C	5.3880120	2.0836109	-5.0378337

F	3.6207481	5.4810342	-0.8790394
F	5.4652132	7.3741720	-1.6243801
F	8.3171696	0.8735620	-6.8465364
C	4.0385444	5.5439885	-2.1494698
C	4.9747479	6.5156007	-2.5270328
C	7.2085978	1.5621958	-6.5511732
C	3.5182986	4.6507160	-3.1065935
C	5.3920536	6.5825585	-3.8658361
F	2.5943289	3.8001657	-2.6560723
O	2.6462073	2.6902865	-4.9761110
F	6.2721445	7.5188289	-4.2504527
C	3.9367187	4.6759394	-4.4466826
C	4.8638613	5.6770664	-4.7944424
C	4.8024559	2.9769816	-5.9609019
C	3.4623393	3.6519890	-5.5208435
F	5.2638967	5.8103763	-6.0719450
C	6.6781923	2.4767106	-7.4693023
F	2.9479348	2.4737297	-8.0789473
C	5.5072351	3.1872432	-7.1586228
C	2.6746403	4.3643440	-6.6506430
F	7.2953748	2.6788083	-8.6418400
C	2.3950778	3.6768321	-7.8477220
F	5.0883436	4.0613128	-8.0890187
F	2.0533962	6.2722538	-5.3094252
C	1.9396655	5.5465295	-6.4377394
C	1.5125813	4.1588492	-8.8196006
F	1.2783636	3.4593976	-9.9348875
C	1.0444778	6.0548675	-7.3940372
C	0.8163644	5.3517945	-8.5831802
F	0.3877257	7.1999488	-7.1566633
F	-0.0495083	5.8222795	-9.4882164
F	4.0613722	-4.2201033	-4.4134778
F	3.8072944	-1.7789015	-5.5012923
C	2.9539722	-3.9101611	-5.0978871
C	2.7947617	-2.6370262	-5.6666120
F	6.1545334	-0.1282221	-9.0157363
F	5.5212996	-1.6634850	-11.1968692
F	2.0995780	-6.1012753	-4.7466373
C	4.9285594	-0.6627089	-9.1233137
C	4.6150899	-1.4538305	-10.2343365
C	1.9609732	-4.8823898	-5.2835568
C	3.9749565	-0.4396820	-8.1108040
C	3.3344830	-2.0224891	-10.3263784
F	4.3892394	0.3128147	-7.0925469
O	2.0132456	0.0927020	-6.0984671
F	3.0106201	-2.7782031	-11.3859241
C	2.6809563	-0.9866020	-8.1815895

C	2.4078504	-1.7967804	-9.3009311
C	1.6522687	-2.2762805	-6.4140874
C	1.6272075	-0.8374414	-7.0356466
F	1.1982607	-2.3700840	-9.4350443
C	0.8284795	-4.5674607	-6.0446356
F	-0.8298138	-1.2081515	-5.5701521
C	0.7000942	-3.2919826	-6.6228936
C	0.2385191	-0.4046961	-7.5614099
F	-0.1208584	-5.4943968	-6.2404510
C	-0.8868546	-0.5100235	-6.7167611
F	-0.3979974	-3.1074731	-7.3741843
F	1.1055286	0.6524222	-9.5373049
C	0.0831814	0.4045408	-8.7010143
C	-2.1157617	0.0814936	-7.0293053
F	-3.1764116	-0.0949709	-6.2298984
C	-1.1325108	1.0256851	-9.0239889
C	-2.2329710	0.8849592	-8.1721212
F	-1.2218813	1.7981420	-10.1148162
F	-3.3955033	1.4825077	-8.4596587
Al	1.3313259	1.6281340	-5.4946981
F	0.3496210	2.3801778	-6.6787321

$Al(OTeF_5)_3$

22

Al	0.1835974	-0.1146592	0.3569691
O	-1.2372156	-1.2623448	0.1166019
Te	-1.9162899	-1.5995101	1.8655923
F	-2.2162052	-1.6232492	3.7539926
F	-3.1163645	-0.1001313	1.8403755
F	-0.7203899	-3.0700708	2.1886576
F	-0.3390383	-0.2886924	2.2360610
F	-3.3318629	-2.8019518	1.4439497
O	0.0832764	1.6445917	0.3409463
Te	-0.7004843	2.7839116	-0.9845874
F	-1.5085901	3.8987505	-2.3188691
F	-1.5442915	1.2560563	-1.8271586
F	0.1037759	4.3334153	-0.1982043
F	0.8074916	2.5939061	-2.1663531
F	-2.2718239	3.0474974	0.0866152
O	1.8241460	-0.8525663	0.7797769
Te	2.6224138	-1.2772519	-0.8975055
F	2.9663359	-1.4968948	-2.7646936
F	3.4553147	0.4421835	-1.0902285
F	1.8044166	-3.0148182	-0.9847164
F	0.7875978	-0.4490305	-1.4579436
F	4.2641903	-2.0491410	-0.3192780

$[FAl(OTeF_5)_3]^-$

23			
O	-1.5990124	-0.2112111	0.4992413
Te	-2.5093300	-1.1620655	1.8566082
F	-2.7638825	-2.7190949	0.7476628
Al	0.1682523	0.0940330	0.2956530
F	2.3243986	-1.6728086	-2.6296335
Te	2.6290393	-1.8817652	-0.7362149
F	3.1886206	-2.1760194	1.0872243
F	-2.4057251	0.2661795	3.1499833
O	0.2574017	1.1212041	-1.1853757
Te	-0.2807880	2.9199922	-1.4081540
F	-1.1518844	3.0274829	0.3183867
O	0.8382188	-1.4860318	-0.2704012
F	-3.4840571	-2.1389983	3.2065015
F	-4.2103812	-0.4818236	1.2671500
F	-0.9041254	-1.9515456	2.6004135
F	-0.8214846	4.7525849	-1.6822523
F	-1.9615090	2.4467521	-2.2275389
F	1.3289164	3.6577025	-0.6391482
F	0.5294139	2.9951853	-3.1520931
F	4.4439144	-2.3261404	-1.2203380
F	3.2269549	-0.0433026	-0.6233762
F	2.2280214	-3.7569081	-0.9037168
F	0.9290274	0.7265986	1.6494183

$[Al(OTeF_5)_4]^-$

29			
Te	11.4208635	0.4117861	14.9209615
F	10.5953687	-0.2200294	13.2890505
F	12.1522926	0.9012485	16.6290383
F	10.6394949	-1.1213592	15.7925228
F	9.7981994	1.3453737	15.3722369
F	12.9509986	-0.7146239	14.5995586
O	12.1907075	1.9361358	14.0989239
Al	12.5138086	2.3670908	12.3971413
O	11.0580945	2.8459716	11.4751998
O	13.1865640	0.9361126	11.5585438
O	13.6087064	3.7814039	12.4269925
Te	9.9625217	4.3820619	11.6823788
Te	13.5904433	0.7170685	9.7154164
Te	15.1623238	4.0599469	13.4814877
F	10.7571243	4.8891723	13.3725259
F	9.0541674	4.0241160	10.0266208

F	8.8211148	5.9235166	11.8790645
F	11.1758468	5.5530215	10.7492051
F	8.5999407	3.3981823	12.6239893
F	13.7832679	-1.1824581	9.9303519
F	11.7544378	0.4566220	9.1910425
F	14.0312782	0.4673862	7.8547254
F	15.4834588	0.9220898	10.0050362
F	13.4417124	2.6058112	9.3208911
F	14.1849770	4.3543536	15.1160256
F	16.7560809	4.3549501	14.5253955
F	16.3356405	3.8244393	11.9723854
F	15.1326821	5.9506013	13.1345226
F	15.3551632	2.1839182	13.9192256

$[(F_5TeO)Al(OTe^R)_3]^-$

89

Te	5.3182376	3.3760276	27.7657427
Te	5.9397162	3.1583667	22.0598840
Te	3.2838666	7.5885041	25.1184162
Te	8.8360324	6.8558228	24.5980514
Al	5.7412089	5.2666932	24.8393175
F	5.2859025	1.9545457	29.1189263
F	3.2163615	3.0556899	25.3103104
F	5.1409144	4.6530459	29.2453570
F	5.4789626	1.9667484	26.4247675
F	2.3974278	5.8259168	25.1390038
F	5.9179829	4.8861092	21.1318603
F	4.0203450	9.3970248	25.1181559
F	3.2496211	5.0281110	22.0827542
F	7.4173306	5.8011726	28.3144209
F	1.5232009	6.4391822	27.6742218
F	2.9323588	3.3174245	30.0797092
F	1.5708065	8.3958933	25.6509732
F	7.6684714	1.0495627	27.7967050
F	5.9941844	1.3738149	22.8534578
F	0.5292960	2.9166992	25.1732596
F	6.3731464	2.3413477	20.3261908
F	4.3342149	0.5255167	20.8735068
F	0.4246014	7.0481165	23.5748854
F	0.6502654	4.5166340	21.5676881
F	4.3649720	7.3410394	31.3227119
F	0.2464261	3.1824591	29.9015629
F	4.7485945	8.7597168	22.4410289
F	-0.9721732	2.9901016	27.4638815
F	5.8271386	8.4084063	26.9681996
F	2.0134334	6.3644698	30.3191500

F	7.8646024	2.4569193	24.5880840
F	10.1815586	5.6155777	25.2395318
F	10.3546602	1.1937621	27.9919132
F	6.2638144	8.3586003	29.6369522
F	8.5376399	7.4542642	26.4068211
O	5.3271736	4.8257480	26.5044018
F	3.9138957	9.1291445	19.9031751
F	8.4476917	4.1462263	20.1487984
F	1.7350295	0.0626415	20.3499069
F	-0.1203426	2.0401578	20.6916556
O	4.9508101	6.8237161	24.5319856
F	7.6990011	8.2460027	23.9061404
F	11.5886645	3.6082615	28.3216991
F	10.1101643	5.9009464	28.5042112
F	10.2631691	8.1528794	24.5429222
F	-0.3761023	7.4431126	21.0335527
F	1.3538889	8.4764034	19.1826048
F	9.3576986	6.3970925	22.7922374
F	10.5436650	2.4773366	24.9273554
O	5.3894827	3.9523610	23.7125750
F	12.1788387	3.3512541	22.9117761
F	11.1144909	4.1716854	20.5310264
O	7.5395856	5.4894765	24.6633356
C	8.6267803	2.8633304	23.5718792
C	7.4559937	3.4272166	28.0146193
C	3.1688071	3.2146212	27.6942970
C	2.6161889	7.8924687	23.0949890
C	2.9197289	3.7997510	21.6832822
C	8.0996113	4.6625033	28.2118576
C	3.6582539	7.4467362	27.2366656
C	3.8818969	2.7881876	21.5305324
C	2.3894472	3.2347360	28.8653276
C	8.2224906	2.2503392	27.9503795
C	2.5295661	3.1023504	26.4469990
C	9.6241781	2.3125175	28.0492549
C	2.6900222	6.9222237	28.1091487
C	1.5622105	3.5502237	21.4135411
C	10.2594498	3.5522635	28.2237407
C	1.1281488	3.0288885	26.3621415
C	9.5009512	4.7314126	28.3074193
C	0.9868748	3.1637755	28.7877559
C	2.9303410	6.8834279	29.4927101
C	8.0693050	3.2836315	22.3526404
C	1.3040828	7.5585356	22.7164492
C	1.1650233	2.2824829	20.9599953
C	3.0747500	8.6210969	20.8112501
C	3.4760196	1.5241589	21.0646192

C	4.1373751	7.3792684	30.0087006
C	4.8702928	7.9260394	27.7586525
C	5.1116770	7.8987742	29.1431718
C	0.3592248	3.0628906	27.5353236
C	0.8739452	7.7577828	21.3920826
C	3.5003531	8.4218515	22.1384189
C	1.7617622	8.2867635	20.4412811
C	8.9148311	3.7268975	21.3217182
C	2.1198539	1.2678582	20.7867248
C	10.8567542	3.3249293	22.7316216
C	10.0171880	2.8786991	23.7645818
C	10.3088526	3.7450930	21.5097451

Me_3SiF

14

Si	-4.4332147	2.8445502	0.0136603
F	-3.0655265	2.0348462	0.4782942
C	-5.3343796	1.7451031	-1.2152561
C	-5.4548540	3.1427226	1.5621348
C	-3.8875008	4.4610192	-0.7745988
H	-5.7243854	2.1859521	2.0541852
H	-6.3973060	3.6758125	1.3177624
H	-4.8972364	3.7590742	2.2966816
H	-3.2451232	4.2789269	-1.6601821
H	-3.3126704	5.0820520	-0.0575447
H	-4.7653435	5.0529253	-1.1083022
H	-5.6010103	0.7697229	-0.7594242
H	-4.7041969	1.5446173	-2.1056851
H	-6.2724422	2.2250356	-1.5637554

$[Me_3Si]^+$

13

Si	-5.1455742	3.3166716	-0.2682431
C	-5.3974682	1.8145283	-1.3026295
C	-5.5126646	3.2600792	1.5350577
H	-5.3285072	2.2566613	1.9692315
H	-6.6020751	3.4726252	1.6610168
H	-4.9616901	4.0361158	2.1028594
H	-5.2545075	0.8779803	-0.7274253
H	-4.7580912	1.8162164	-2.2081297
H	-6.4579218	1.8248899	-1.6533284
C	-4.6826721	4.9115374	-1.0630159
H	-5.6301339	5.4225430	-1.3613682
H	-4.0892416	4.7651153	-1.9875734
H	-4.1535424	5.5913861	-0.3655318

SbF_5

6			
F	-0.0803553	0.7687903	-0.2359865
F	-1.5644659	-0.5997890	-2.8779445
F	1.7485244	-0.4479695	-2.6911780
F	0.0472954	-1.8102458	-1.0765029
F	-0.0080819	1.6200399	-2.8104591
Sb	0.0253134	-0.0936359	-1.9403790

$[SbF_6]^-$

7			
F	-0.0000000	-0.0000000	-0.0004107
F	-1.9513392	0.0000000	-1.9517493
F	-0.0000000	1.9513392	-1.9517493
F	1.9513392	0.0000000	-1.9517493
F	-0.0000000	-1.9513392	-1.9517493
F	-0.0000000	-0.0000000	-3.9030840
Sb	0.0000000	-0.0000000	-1.9517480

7 References

(1) Borys, A. M. An Illustrated Guide to Schlenk Line Techniques. *Organometallics* **2023**, *42* (3), 182–196.

(2) Bastide, J. P.; El Hajri, J.; Claudy, P.; El Hajbi, A. A New Route to Alkali Metal Aluminum Hydrides $MAlH_4$ with M = Na, K, Rb, Cs and Structural Features for the Whole Family with M = Li to Cs. *Synth. React. Inorg. Met.-Org. Chem.* **1995**, *25* (7), 1037–1047.

(3) Wegener, D.; Pérez-Bitrián, A.; Limberg, N.; Wiesner, A.; Hoffmann, K. F.; Riedel, S. A Highly Sterically Encumbered Boron Lewis Acid Enabled by an Organotellurium-Based Ligand. *Chem. Eur. J.* **2024**, *30* (36), e202401231.

(4) Harris, R. K.; Becker, E. D.; Cabral de Menezes, S. M.; Granger, P.; Hoffman, R. E.; Zilm, K. W. Further conventions for NMR shielding and chemical shifts (IUPAC Recommendations 2008). *Pure Appl. Chem.* **2008**, *80* (1), 59–84.

(5) Sheldrick, G. M. Structure determination revisited. *Acta Cryst. A* **2015**, *71* (a1), 9.

(6) Sheldrick, G. M. Crystal structure refinement with SHELXL. *Acta Cryst. C* **2015**, *71* (Pt 1), 3–8.

(7) Dolomanov, O. V.; Bourhis, L. J.; Gildea, R. J.; Howard, J. A. K.; Puschmann, H. OLEX2:a complete structure solution, refinement and analysis program. *J. Appl. Cryst.* **2009**, *42* (2), 339–341.

(8) K. Brandenburg, DIAMOND, Crystal Impact GbR, Bonn, 2014.

(9) TURBOMOLE GmbH. *TURBOMOLE V7.6*: *a development of University of Karlsruhe and Forschungszentrum Karlsruhe*, 2022.

(10) Becke, A. D. Density-functional exchange-energy approximation with correct asymptotic behavior. *Phys. Rev. A* **1988**, *38* (6), 3098–3100.

(11) Lee, C.; Yang, W.; Parr, R. G. Development of the Colle-Salvetti Correlation-energy Formula into a Functional of the Electron Density. *Phys. Rev. B* **1988**, *37* (2), 785–789.

(12) Vosko, S. H.; Wilk, L.; Nusair, M. Accurate Spin-dependent Electron Liquid Correlation Energies for Local Spin Density Calculations: a Critical Analysis. *Can. J. Phys.* **1980**, *58* (8), 1200–1211.

(13) Grimme, S.; Brandenburg, J. G.; Bannwarth, C.; Hansen, A. Consistent structures and interactions by density functional theory with small atomic orbital basis sets. *J. Chem. Phys.* **2015**, *143* (5), 54107.

(14) Sierka, M.; Hogekamp, A.; Ahlrichs, R. Fast evaluation of the Coulomb potential for electron densities using multipole accelerated resolution of identity approximation. *J. Chem. Phys.* **2003**, *118* (20), 9136–9148.

(15) Weigend, F.; Ahlrichs, R. Balanced Basis Sets of Split Valence, Triple Zeta Valence and Quadruple Zeta Valence Quality for H to Rn: Design and Assessment of Accuracy. *Phys. Chem. Chem. Phys.* **2005**, *7* (18), 3297–3305.

(16) Grimme, S.; Antony, J.; Ehrlich, S.; Krieg, H. A consistent and accurate ab initio parametrization of density functional dispersion correction (DFT-D) for the 94 elements H-Pu. *J. Chem. Phys.* **2010**, *132* (15), 154104.

(17) Zhao, Y.; Truhlar, D. G. Design of density functionals that are broadly accurate for thermochemistry, thermochemical kinetics, and nonbonded interactions. *J. Phys. Chem. A* **2005**, *109* (25), 5656–5667.

(18) Weigend, F.; Furche, F.; Ahlrichs, R. Gaussian basis sets of quadruple zeta valence quality for atoms H–Kr. *J. Chem. Phys.* **2003**, *119* (24), 12753–12762.

(19) Zapf, L.; Riethmann, M.; Föhrenbacher, S. A.; Finze, M.; Radius, U. An easy-to-perform evaluation of steric properties of Lewis acids. *Chem. Sci.* **2023**, *14* (9), 2275–2288.
